# Thymoquinone upregulates IL17RD in controlling the growth and metastasis of triple negative breast cancer cells in vitro

**DOI:** 10.1186/s12885-022-09782-z

**Published:** 2022-06-27

**Authors:** Md. Asaduzzaman Khan, Meiling Zheng, Jiewen Fu, Mousumi Tania, Jun Li, Junjiang Fu

**Affiliations:** 1 Key Laboratory of Epigenetics and Oncology, The Research Center for Preclinical Medicine, Southwest Medical University, Luzhou, 646000 Sichuan China; 2 Division of Molecular Cancer Biology, The Red-Green Research Center, Dhaka, 1205 Bangladesh; 3 Nature Study Society of Bangladesh, Dhaka, Bangladesh; 4 Department of Pathology, Baoji Central Hospital, Baoji, 721008 Shaanxi China

**Keywords:** Triple negative breast cancer, Epigenetics, Thymoquinone, ChIP-Seq, Mehtylation, IL17RD

## Abstract

**Background:**

Triple negative breast cancer (TNBC) is a molecular subtype of breast cancer, which is a major health burden of females worldwide. Thymoquinone (TQ), a natural compound, has been found to be effective against TNBC cells, and this study identified IL17RD as a novel target of TQ in TNBC cells.

**Methods:**

We have performed chromatin immunoprecipitation Sequence (ChIP-Seq) by MBD1 (methyl-CpG binding domain protein 1) antibody to identify genome-wide methylated sites affected by TQ. ChIP-seq identified 136 genes, including the tumor suppressor IL17RD, as a novel target of TQ, which is epigenetically upregulated by TQ in TNBC cell lines BT-549 and MDA-MB-231. The IL17RD expression and survival outcomes were studied by Kaplan–Meier analysis.

**Results:**

TQ treatment inhibited the growth, migration, and invasion of TNBC cells with or without IL17RD overexpression or knockdown, while the combination of IL17RD overexpression and TQ treatment were the most effective against TNBC cells. Moreover, higher expression of IL17RD is associated with longer survival in TNBC patients, indicating potential therapeutic roles of TQ and IL17RD against TNBC.

**Conclusions:**

Our data suggest that IL17RD might be epigenetically upregulated in TNBC cell lines by TQ, and this might be one of the mechanisms by which TQ exerts its anticancer and antimetastatic effects on TNBC cells.

**Supplementary Information:**

The online version contains supplementary material available at 10.1186/s12885-022-09782-z.

## Introduction

In the era of large development of biomedical science, cancer is still not yet curable fully and remains as one of the major threats of modern life. Cancer is the second leading cause of death after myocardial infarction. Because of the adverse effects of existing therapeutic strategies like chemotherapy or radiation, scientists are now focusing on ‘targeted’ therapies that can selectively kill cancer cells without affecting normal cells [1], and this approach has opened a new dimensions in cancer research. Breast cancer (BC) is the most common malignancy among women [2]. Triple negative breast cancer (TNBC) is a molecular subtype of breast cancer and is characterized by the absence of estrogen receptor (ER), progesterone receptor (PR), and human epidermal growth factor receptor 2 (HER2) [3]. Unlike ER + BC and HER2 + BC, TNBC patients are usually unresponsive to clinically approved therapies [4]. At this moment, the primary treatment options for TNBC were limited to conventional chemotherapies like anthracyclines (e.g., doxorubicin) and taxane based therapeutics [5], but recently more drugs are available for TNBC treatment. For example, capecitabine, gemcitabine, eribulin, cisplatin, carboplatin, olaparib, talazoparib are used too as chemotherapeutic drugs, and as immunotherapy, atezolizumab along with albumin-bound paclitaxel, or pembrolizumab plus chemotherapy [6]. Very recently, US food and drug administration (FDA) approved sacituzumab govitecan for TNBC [6, 7]. Still, TNBC management is poor compared to other BC subtypes. To develop more specific ‘targeted’ therapies for TNBC, identifying novel potential targets of chemotherapeutic agents and developing effective therapies for TNBC treatment are essential.

Thymoquinone (2-methyl-5-isopropyl-1,4-benzoquinone/ TQ) is a phytochemical compound found in black cumin (Nigella sativa) with a long history of medicinal use in South and South-eastern Asia, Arab, Africa and Mediterranean regions [8, 9]. In recent years, many scientific researchers have revealed the anticancer potential of TQ, which mainly exerts its effect through its antioxidant activity, interfering with DNA structure and synthesis, immunomodulatory activity and targeting numerous proteins involved in different signaling pathways [10–12]. Methylation of DNA is one of the major epigenetic mechanisms of gene regulation. In this process, methyl groups are added to the DNA molecule, especially at the promoter region, methylation typically acts to repress gene transcription. Studies indicated that TQ might modify the methylation status of a target gene affecting the expression of the corresponding proteins [12, 13]. However, the effects of TQ on the methylation of different genes are largely unexplored.

MBD1 (methyl-CpG binding domain protein 1) is a member nuclear protein family with a methyl-CpG binding domain (MBD), which can recognize and bind specifically to methylated DNA. This protein family contains a MBD at the N-terminus for binding to methylated CpGs and other protein interactions, CXXC-type zinc finger domains, among which the third CXXC domain (CXXC3) enables mediating non-methylated CpG dinucleotide binding, and a transcriptional repression domain (TRD) at the C-terminus for transcription repression and in other protein interactions. Through binding to CpG islands in promoters where the DNA is methylated, targeted gene transcription may be repressed [14, 15].

To identify new targets of TQ in TNBC, in this study, a genome-wide methylation analysis of TNBC was performed by MBD1 ChIP sequencing, and here our study revealed that TQ epigenetically upregulates interleukin-17 receptor type D (IL17RD) expression while reducing TNBC cell growth and metastasis. IL17RD is a transmembrane protein, which has a tumor suppressor role, and thus upregulating IL17RD by TQ may open a new door in cancer immunotherapeutic research and TQ based drug development.

## Materials and methods

### Cell culture and treatment

TNBC cell lines BT-549 (ATCC#HTB-122™) and MDA-MD-231 (ATCC#HTB-26™) were cultured in RPMI1640/ DMEM (Gibco, Thermo Fisher ScientificTM, Beijing, China) media supplemented with 10–15% fetal bovine serum (FBS) (Gibco, Life Technologies, NSW, Australia) in cell culture dishes or flasks. TQ was purchased from Sigma-Aldrich, China (Cat #274,666) and prepared in dimethyl sulfoxide (DMSO).

### DNA dot blot assay

BT-549 cells were treated with TQ (5 µM) for 6 h and DNA was extracted for dot blot analysis for whole genome methylation status, especially at CpG islands of the genome [16]. A grid was drawn on the nitrocellulose membrane by a pencil to indicate the region going to blot, and 2 µl DNA with different conc. (10–320 ng) were spotted on a grid with a pipette. The membrane was dried under UV-linker for 6 min. Membrane was then blocked with anti-5-methylcytosine (anti 5’MC) antibody (#ab10805, Abcam, USA) with dilution fold: 1:1000 in 5% BSA for 3 h at RT. Membrane was then washed with TBST/PBST (3 × 5 min), and incubated with secondary antibody [IRDye 800CW-conjugated goat (polyclonal) anti-mouse IgG (Li-Cor)] (Dilution fold: 1:2000 in 5% BSA) for 2 h at RT in dark. After washing with TBST/PBST (3 × 5 min) in dark, the membrane was scanned with an Odyssey infrared imaging system (Li-Cor) in the 800 nm channel.

### ChIP sequencing (ChIP-Seq) and verification

BT-549 cells were treated with TQ (5 µM) for 6 h and chromatin immune precipitation (ChIP) was performed following standard protocols [17–20], using a specific antibody (anti- MBD1) (#ab2846, Abcam, USA). Anti-MBD1 could precipitate a specific genomic regions, which are methylated. The ChIP products were sequenced by library construction and bioinformatics analysis. The gene ontology (GO) analysis for multiple pathways was performed. The genomic methylated regions affected by TQ treatment were identified. The specific regions were verified by using PCR reactions. For 14 K upstream of IL17RD (-1) [chr3: 57,088,981–57,166,375], the primer sequences are as follows: F: 5’-GTGGCTGCTCCTCCTGTATG-3’; R: 5’-TATGGCTCTCACAGGGGAAT-3’, and for immediate upstream region of IL17RD (-1), [chr3:57,164,775–57165375] the primer sequences are as follows: F: 5’-GATATTTGTGGTTGGAGGGTAAG-3’, R: 5’-AAAAAAATAAAACCCAAACC-3’.

### RT-PCR

TNBC cell lines (BT-549 and MDA-MD-231) were treated with TQ (5 µM) for 12 h, and total RNAs were collected by using RNAsimple Total RNA kit (#DP419, TIANGEN, Beijing, China), following the manufacturer’s guideline. RNA concentration was measured by using ND-2000 UV/Vis spectrophotometer (NanoDrop 2000, Thermo Scientific, DC, USA) and final concentration was made 150 ng/µL for cDNA synthesis. cDNA was synthesized by reverse transcription reaction by using ReverTra Ace®qPCR RT Master Mix (Toyobo, Osaka, Japan) using (reverse transcriptase/RT-PCR), following the manufacturer’s guideline. The RT reaction system was 10 μL, including 5 × RT Master Mix 2 μL, RNA template 500 ng, and ddH2O was added to make the final volume 10 μL, and the RT-PCR program was as follows: 37 °C for 15 min, 50 °C for 5 min, then at 98 °C for 5 min, and finally hold at 4 °C. Then RT-PCR product (cDNA) was then diluted fivefold and kept in a -20 °C refrigerator, or used it directly in the next experiment. The PCR reaction was performed using 2xTaq PCR Master Mix (#KT121221, TIANGEN, Beijing, China) in Applied Biosystem Veriti 96 thermal cycler (Life TechnologiesTM, Singapore). The reaction system used for PCR amplification was 10 μL, consisting primers 1 μL, 2 × Taq PCR Master mix 5 μL, cDNA 2 μL, ddH2O 2 μL. Reaction steps were as follows: pre-denaturation at 94 °C for 3 min, then 30 cycles of denaturation at 94 °C for 30 s, annealing at 55 °C for 30 s and extension at 72 °C for 1 min, and finally extension at 72 °C for 5 min and storage at 4 °C. PCR products were then run on 1.5% agarose gel electrophoresis, and ethidium bromide eluted PCR bands were visible in the imaging system (Universal Hood II, Bio-Rad Lab, USA). Images were quantified by using ImageJ software (National Institutes of Health, Rockville, MD, USA), and comparative analysis of mRNA expression was performed on the basis of housekeeping gene *GAPDH* expression. Analysis was performed in triplicate. For IL17RD amplification, the primer sequences were as follows: F: 5’-CAGGACTTCTGTGGCTGTGA-3’, R: 5’-GCCACCTCCTTTGTGTTTGT-3’. GAPDH was used as control, and its primer sequences were F: 5’-GAGTCAACGGATTTGGTCGT-3’, R: 5’-TTGATTTTGGAGGGATCTCG-3’.

### Western blotting

TNBC cells lines (BT-549 and MDA-MD-231) were treated with TQ (5 µM) for 36 h, and cellular proteins were collected using RIPA buffer (Beyotime, Jiangsu, China) and Phenylmethylsulfonyl fluoride (PMSF) (Thermo Fisher Scientific, China). Proteins were separated on PAGE and transferred to a nitrocellulose membrane. The membrane was incubated with anti-IL17RD antibody (#ab111553, Abcam, USA), for 8–12 h, and after washing with TBST, incubated for 2–3 h with anti-rabbit IgG, HRP-linked antibody (#7074, Cell Signaling Tech, Danvers, USA). After washing again with TBST, the protein bands were visualized by chemiluminescent reaction (Immobilin®Crescendo, Western HRP Substrate, Millipore, Billerica, USA) in digital imaging system (Universal Hood II, Bio-Rad Lab, Segrate, Italy). Images were quantified by using ImageJ software (National Institutes of Health, Rockville, MD, USA), and comparative analysis of protein expression was performed on the basis of housekeeping protein β-actin expression [anti- β-actin antibody, (#20,536–1-AP, Proteintech, China)]. Analysis was performed in triplicate.

### Real-time cell analysis

The real-time analysis (growth, migration, and invasion) of TNBC cells was performed by using real-time cell analyzer (xCELLigence RTCA DP, Roche, Penzberg, Germany) [10, 13, 17]. Based on the electric impulse generated in the gold particle of E-Plate and CIM-plates of the analyzer, the real-time data on growth, migration, and invasion were obtained.

### IL17RD overexpression

IL17RD constructed in the plasmid (pUNO1-IL17RD) was purchased from InvivoGen, San Diego, USA, and the plasmids were amplified in DH5α competent cells to blasticidin + LB medium. pUNO1-IL17RD was then transfected into BT-549 cells by using Lipofectamine®2000 reagent (Invitrogen, USA). Transfection efficiency was checked by fluorescence microscopy and Western blotting. IL17RD-overexpressed cells were grown with or without TQ (5 µM), and cell growth, migration, and invasion were monitored by real-time cell analysis.

### IL17RD knockdown

IL17RD was knocked down by shRNA technology. IL17RD-human 29mer shRNA constructs in the lentiviral GFP vector were purchased from OriGene tech (Rockville, USA), and shRNA specific to IL17RD was introduced into BT-549 cells via transduction following OriGene HuSHTM shRNA application guideline. IL17RD-human 29mer shRNA sequences are as follows: GCCGAAGGTCTTTCTCTGCTATTCCAGTA.

### Pyrosequencing assays

BT-549 and MDA-MB-231 cells were treated with TQ (5 µM) for 12 h, and DNA materials were extracted by using TIANamp genomic DNA kit (TianGen, Beijing, China). DNA samples were treated with bisulfite and site-specific methylation level was quantified by pyrosequence technology by using Qiagen bisulfite kit (HuaDe Biotechnology, Beijing, China), and the primers used were as follows: F: 5’-GATATTTGTGGTTGGAGGGTAA-3’; R: 5’-ACCCCACCTCATTAACAACACA-3’. This section was rich in CpG islands (chr3:57,164,775–57,165,375) and the upstream region of ‘- ‘strand of IL17RD. Each site was analyzed as a C/T-polymorphism and the percentage of methylation was displayed in a small colored box just above each CpG site, where 100% denotes a fully methylated C, 0% denotes unmethylated C, and intermediate C/T percentages denote partial methylation in cellular DNA.

### Immunohistochemistry and Kaplan–Meier survival analysis

Data for immunohistochemistry (IHC) with IR17RD (Cat # HPA043550, Sigma-Aldrich), which were from the Human Protein Atlas (HPA) project (https://www.proteinatlas.org/ENSG00000144730-IL17RD/pathology/breast+cancer#img) [21, 22], and Kaplan–Meier survival analysis were performed as described [23, 24].

### Statistical analysis

Data was analyzed by one-way ANOVA and then post hoc comparison by using the SPSS v. 20 software (IBM, NY, USA), and MS-Excel 2010 (Microsoft, Washington, DC, USA). Results were usually presented as mean ± SD. *p* < 0.05 was considered as significant difference.

## Results

### Thymoquinone interferes with the epigenetic system of TNBC cells

**Fig. 1:**
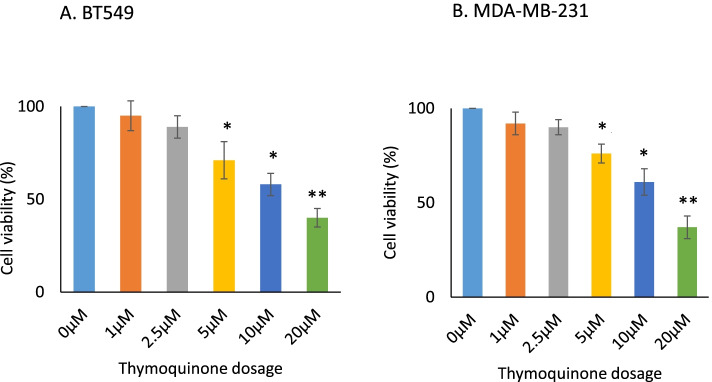
Effect of TQ on TNBC cell viability. TQ shows dose-dependent growth inhibitory effects on BT-549 (**A**) and MDA-MB-231 (**B**). TQ was treated for 24 h in different dosage. Bars are presented as mean ± SD. *, *p* < 0.05; ** *p* < 0.01 (*N* = 3)

**Fig. 2:**
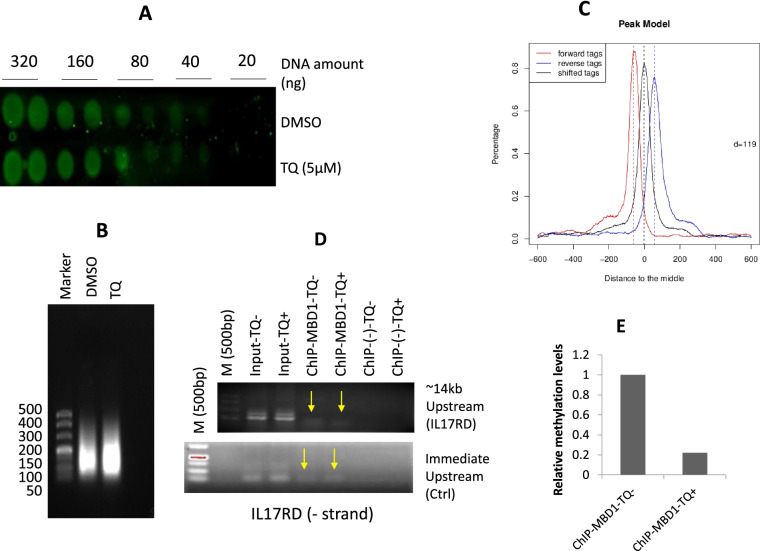
Effect of TQ on TNBC cell methylation status. **A **Dot blot by anti-5-methylcytosine (5-mC) antibody with and without TQ treatment indicating DNA amount. **B** Sonicated genomic DNA. Lane “Marker” indicates DNA molecular weight size (bp). **C** Dual peak model results. **D** For methylation status, ChIP-Seq was performed using anti-MBD1 antibody, which identified > 1000 segments of the genome, of which the methylation level was affected by TQ. These segments were spanned through 136 genes. We verified the sequence laid to 14 kb and immediately upstream of IL17RD by PCR. Immediate upstream region was set as a control (Ctrl) for ChIP verification. Agarose gel electrophoresis of PCR products indicated that the methylation level of these sequences was inhibited by TQ treatment. **E** Quantitative analysis results from Fig. 1D. The ChIP results of immediate upstream as a control were used to normalize to ChIP results of IL17RD promoter region

Firstly, we found that TQ dose-dependently inhibits the growth (cell viability) of BT-549 and MDA-MB-231 cells (Fig. 1), which is consistent with our previous reports [8, 10]. Here TQ was treated for 24 h in different dosage and cell viability or cytotoxicity was assayed by using Cell Counting Kit-8 (CCK-8) analysis kit [11]. To examine whether TQ effects the global methylation level in the genome, DNA dot blotting by anti-5-methylcytosine (5-mC) antibody was performed and our preliminary experiments showed that there are no significant changes in total methylation level (Fig. 2A). The ChIP sequencing by MBD1 antibody was further performed; the results are shown in Fig. 2B ~ D and Tables S1, S2 and S3. After sonication, the DNA fragments were ranged between 100 ~ 400 bps (Fig. 2B); and after ChIP-Seq by comparing to the reference genome, a total of 2,570,999 reads were revealed by TQ treatment and a total of 3,526,178 reads were revealed without TQ treatment, and from them, 2,074,277 (80.70%) and 2,847,148 (80.74%) of reads were mapped successfully to human genome with and without TQ treatment, respectively. By MACS analysis, 1611 peaks were revealed and the detail information are listed in Table S1. Using these peak data, a dual peak model was constructed indicating differences between the two groups with and without TQ treatment (Fig. 2C). Thus, we screened 136 peak-related genes which may have changed epigenetically (hypermethylated/hypomethylated) by TQ treatment (Table S2). The gene ontology (GO) analysis revealed that multiple pathways are involved, including cytokine mediated signaling pathway, positive regulation of response to_stimulus, and positive_regulation_of_cell_communication, etc. (Table S3).

### IL17RD is one of the TQ regulated genes by DNA methylation

**Fig. 3:**
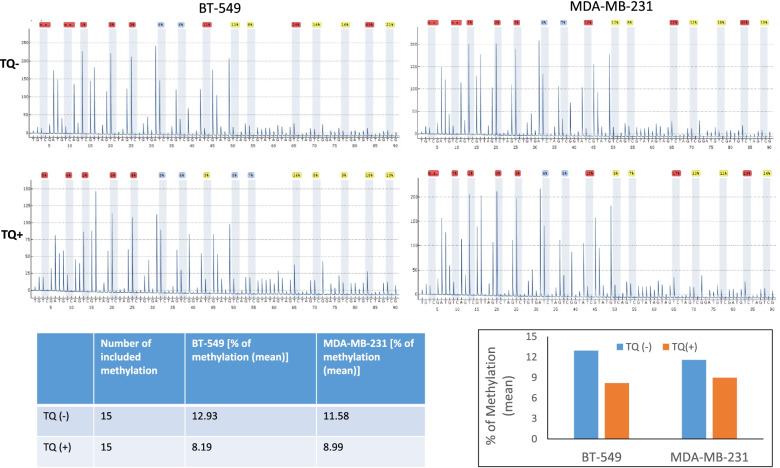
Effect of TQ on methylation status at the proximal promoter region of the gene of IL17RD (- strand). Total 15 regions of the proximal promoter of the gene of IL17RD were checked by pyrosequencing, and the methylation rate was found lower in TQ treated BT-549 cells (8.19%) than in nontreated cells (12.93%), while in the case of MDA-MB-231 cells, methylation rate was also found lower in TQ treated cells (8.99%) than in non-treated cells (11.58%)

Through comprehensive analysis and on the basis of the gene’s link with breast cancer, out of 136 peak-related genes, 12 were selected for further validation (Table 1). ChIP verification by PCR confirmed the hypomethylation of upstream regions of ‘– ‘strand of IL17RD (Fig. 2D) (Table 1, in red), showing nearly 78% levels of methylation lower at the IL17RD promoter region than that of the control (Ctrl) region by quantitative analysis (Fig. 2E), which might be responsible for the upregulation of IL17RD in TNBC cell lines BT-549 and MDA-MB-231. For further confirmation, we have used bisulfite based site-specific methylation level analysis by pyrosequencing. Total 15 points were analyzed in a genomic sequence [chr3:57,164,775–57,165,375 (‘-’strand)] rich in CpG island. In control BT-549 cells, the average methylation rate of these points were 12.93%, which was reduced by TQ treatment to 8.19%, while in the case of MDA-MB-231 cells, the methylation rate was found lower in TQ treated cells (8.99%) than in non-treated cells (11.58%) (Fig. 3).

**Table 1:** The selected genes for further validation

Hgnc_id	Hgnc_ symbol	Ensembl_ gene_id	Chr	Strand	Start_ position	End_ position	Description
HGNC:14,638	ABCA13	ENSG00000179869	7	1	48,171,458	4.9E + 07	ATP binding cassette subfamily A member 13 [Source:HGNC Symbol;Acc:HGNC:14638]
HGNC:38	ABCA8	ENSG00000141338	17	-1	68,867,292	6.9E + 07	ATP binding cassette subfamily A member 8 [Source:HGNC Symbol;Acc:HGNC:38]
HGNC:10,634	CCL7	ENSG00000108688	17	1	34,270,221	3.4E + 07	C–C motif chemokine ligand 7 [Source:HGNC Symbol;Acc:HGNC:10634]
HGNC:2673	DAP3	ENSG00000132676	1	1	155,687,960	1.6E + 08	death associated protein 3 [Source:HGNC Symbol;Acc:HGNC:2673]
HGNC:17,616	IL17RD	ENSG00000144730	3	-1	57,089,982	5.7E + 07	interleukin 17 receptor D [Source:HGNC Symbol;Acc:HGNC:17616]
HGNC:6122	IRF7	ENSG00000185507	11	-1	612,553	615,999	interferon regulatory factor 7 [Source:HGNC Symbol;Acc:HGNC:6122]
HGNC:9534	PSMA5	ENSG00000143106	1	-1	109,399,031	1.1E + 08	proteasome subunit alpha 5 [Source:HGNC Symbol;Acc:HGNC:9534]
HGNC:9630	PTN	ENSG00000105894	7	-1	137,227,341	1.4E + 08	pleiotrophin [Source:HGNC Symbol;Acc:HGNC:9630]
HGNC:11,639	TCF7	ENSG00000081059	5	1	134,114,711	1.3E + 08	transcription factor 7 [Source:HGNC Symbol;Acc:HGNC:11639]
HGNC:12,962	TRIM26	ENSG00000234127	6	-1	30,184,455	3E + 07	tripartite motif containing 26 [Source:HGNC Symbol;Acc:HGNC:12962]
HGNC:16,278	TRIM7	ENSG00000146054	5	-1	181,193,924	1.8E + 08	tripartite motif containing 7 [Source:HGNC Symbol;Acc:HGNC:16278]
HGNC:16,265	WNT5B	ENSG00000111186	12	1	1,529,891	1,647,243	Wnt family member 5B [Source:HGNC Symbol;Acc:HGNC:16265]

### Thymoquinone upregulates IL17RD expression in TNBC cells

**Fig. 4:**
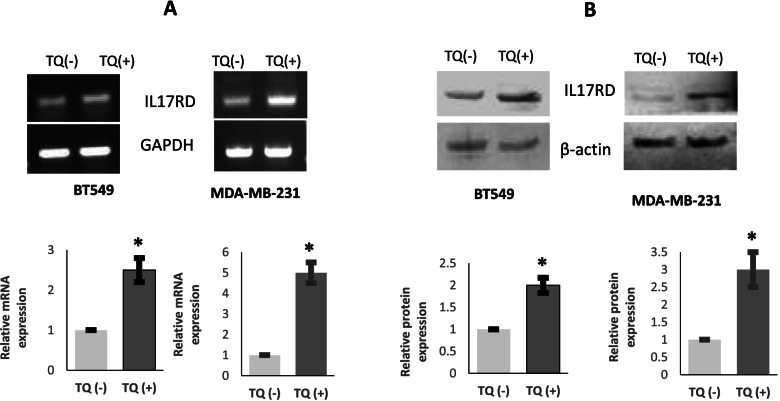
TQ upregulates IL17RD expression in TNBC cells. **A** Treatment of TQ (5 µM) increased the mRNA level expression of IL17RD in both of BT-549 and MDA-MB-231 cells. **B** Treatment of TQ (5 µM) increased the protein level expression of IL17RD in both of BT-549 and MDA-MB-231 cells. Bars are presented as mean ± SD. *, *p* < 0.05 (*N* = 3)

Given that IL17RD promoter was hypomethylated by TQ treatment, then we wanted to know whether IL17RD expression is upregulated at RNA level and protein level upon TQ treatment. Thus, PCR amplification and western blotting were performed, and we found that usually IL17RD is very low expressed in these cell lines, but TQ treatment increased the expression of IL17RD in BT-549 and MDA-MB-231 cells at both mRNA and protein levels (Fig. 4), which is consistent with the hypomethylation effect of TQ in IL17RD upstream regions.

### Thymoquinone controls TNBC cell growth, migration, and invasion associated with IL17RD regulation

**Fig. 5:**
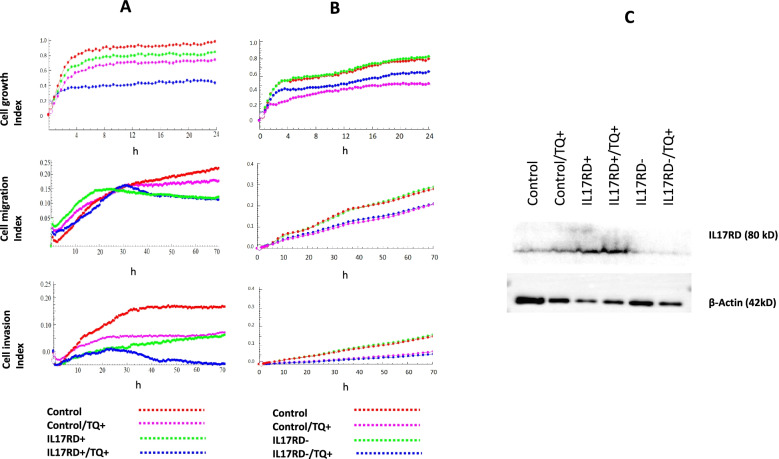
Role of IL17RD overexpression and knockdown on BT-549 TNBC cells, and the effect of TQ on these. **A** IL17RD overexpression had a slight effect on cell growth index but clearly decreased the cellular migration and invasion, which was further synergized by TQ. **B** IL17RD knockdown had no obvious effect on cell growth, migration, and invasion index, but treatment with TQ certainly had a decreasing effect on cell growth, migration, and invasion index. **C** Western blot bands indicates IL17RD expression, and confirms the overexpression and knockdown efficiency

The upregulation of IL17RD is probably associated with the cell growth inhibiting effects of TQ in TNBC cells. Like our previous studies [10, 13], we again showed that TQ has an inhibitory effect on the growth, migration, and invasion characteristics of TNBC cells (Fig. 5). Inducible overexpression of IL17RD in BT-549 cells had a mild effects on cell growth, but clearly inhibited cell migration and invasion (Fig. 5A). However, knockdown of IL17RD did not show further effect on cell growth, migration, and invasion of BT-549 cells (Fig. 5B). Western blot analysis confirmed the efficiency of overexpression or knockdown of IL17RD (Fig. 5C). TQ treatment inhibited the growth, migration, and invasion of BT-549 cells with or without IL17RD overexpression or knockdown, however, the combination of IL17RD overexpression and TQ treatment were most effective. This indicates that IL17RD is a potent target of TQ for its anticancer activity, but there are more mechanisms of action of TQ other than targeting IL17RD.

### High expression of IL17RD is associated with longer survival in TNBC patients

**Fig. 6:**
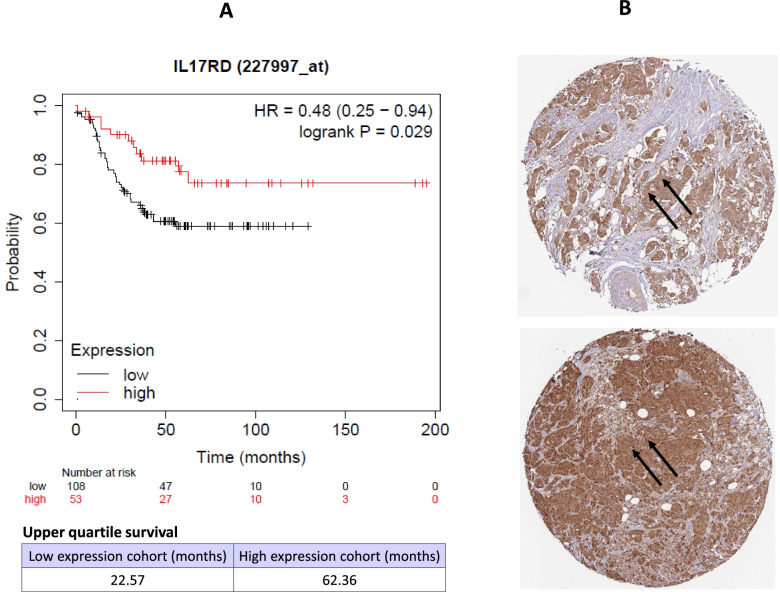
Kaplan–Meier survival results and immunohistochemistry images. **A** Kaplan–Meier plots for the expression of IL17RD in TNBC patients with upper quartile survival. The log-rank *p* = 0.029. **B** Representative images for Immunohistochemistry with high expression of IL17RD in duct carcinoma. Upper panel, patient ID 1874, high staining; bottom panel, patient ID 4193, high staining

Given the fact that TQ controls TNBC cell growth, migration, and invasion associated with IL17RD regulation, further clinical relationships between IL17RD expression and survival outcomes were also studied by Kaplan–Meier survival analysis. The results of recurrence free survival (RFS) analysis showed that high expression of IL17RD is correlated with longer survival (high expression cohort, 62.36 months) and low expression of IL17RD is correlated with shorter survival (low expression cohort, 22.57 months) in TNBC patients, respectively (Fig. 6A, *p*-value < 0.05), and the hazard ratio (HR) for TNBC was 0.48 (95% CI, 0.25–0.94), indicating that IL17RD may be a prognostic or therapeutic marker for TNBC patients. IHC analysis for IR17RD protein indicated weak or no immunoreactivity in the cytoplasmic/membranous in lobular carcinoma patients but strong immunoreactivity in the cytoplasmic/membranous/nuclear locations in duct carcinoma patients (Fig. 6B).

## Discussion

IL17RD, also known as similar expression to FGF (Sef), has been indicated as a tumor suppressor in different cancers more than a decade ago [25]. Although the role of IL17 and IL17Rs in cancer are a bit controversial, as some studies reported their role in cancer progression, and indicated IL17RD as a target for cancer therapeutics [26]. But many studies indicated its role in regulating cancer progression and metastasis in different cancer types, like prostate cancer, breast cancer, etc. [27–30]. Mechanistically, IL17RD regulates epithelial to mesenchymal transition (EMT) in breast cancer cell lines, and loss of its function promotes EMT and cancer metastasis [29, 30]. Our study also found that TNBC cells have lower expression of IL17RD, and upregulation of IL17RD is associated with controlling the growth and metastasis of TNBC cell lines. While searching for the novel target of the anticancer molecule, TQ, we found that IL17RD is a potent target of TQ. In TNBC cells, TQ upregulated IL17RD expression, which was found to be associated with TQ’s anticancer and antimetastatic (anti-migratory and anti-invasive) activities. Furthermore, high expression of IL17RD was associated with longer survival in TNBC patients, indicating the importance of therapeutic or prognostic roles for TQ and IL17RD in TNBC. However, of course, IL17RD is not the sole target of TQ, as many other pathways are targeted by TQ in breast cancer or other cancer types, including TNBC [10, 12, 31]. In fact in this study, we found that even in IL17RD-knockdown cells, TQ exerts anticancer effects. What we report here is, IL17RD is identified as a new target of TQ.

The anticancer and antimetastatic role of TQ in preclinical studies has been confirmed by numerous studies in recent years, and this small molecular natural product has received recognition as a promising anticancer molecule for future drug development [11]. The interference of TQ with epigenetic machinery is well documented in different cancer cells [32–34]. In this study, we took an attempt to study the genome-wide epigenetic role of TQ in TNBC cells (BT-549, MDA-MB-231). DNA dot blotting did not show an obvious effect on whole genome methylation status, probably because TQ methylates some regions of the genome, while demethylates some regions [33]. To identify specific target genes of TQ, we made ChIP-Seq, and found a total of 136 genes, of which the proximate sequence methylation statuss were affected by TQ treatment. From these, we identified IL17RD, of which the proximate sequences were hypomethylated and consequently its expression was upregulated by TQ treatment. More importantly, these were found associated with the antimetastatic role of TQ in TNBC cells and survival in TNBC patients. TNBCs have higher migration and invasion characteristics. If cells are treated with TQ or IL17RD is inducibly overexpressed, migration and invasion are decreased (Fig. 5A). Therefore, it postulates that IL17RD overexpression might decrease migration and invasion, which is further potentiated by TQ. However, in IL17RD knocked-down cells, migration and invasion did not change (further increased), because in these cells, to further increase migration and invasion, many other factors might be related (Fig. 5B). However, treating TQ decreased migration and invasion (usual effect). TQ treatment did not upregulate IL17RD in IL17RD-knocked-down cells, because we made shrna-mediated knocked down of IL17RD, which probably minimized TQ effect. Regulating the inflammatory pathways is one of the mechanisms of TQ to combat cancer and inflammation-associated diseases [35, 36].

## Conclusion

TQ is considered among the promising anticancer molecules for future drug development. Although preclinical studies have enough evidence to support the anticancer and antimetastatic role of this natural product, clinical studies are not yet approved. These studies further confirm the anticancer and antimetastatic role of this compound through the epigenetic modulation of IL17RD, which also indicates the possible role of TQ in the immunomodulation of cancer cells, however, further research is necessary to clarify the immunotherapeutic role of TQ.

## Supplementary Information

**Additional file 1.** 

**Additional file 2.** 

**Additional file 3.** 

**Additional file 4: Table S1.** Methylated peaks chr.

**Additional file 5: Table S2.** Methylated related genes.

**Additional file 6: Table S3.** GO analysis result.

## Data Availability

All data used in this study was generated by experimental studies and available in this paper. ChIP-Seq data is supplied as supplemental files, and also deposited in https://www.ncbi.nlm.nih.gov/geo/query/acc.cgi?acc=GSE178334.
