# Supplementary figures and images for: Thymoquinone upregulates IL17RD in controlling the growth and metastasis of triple negative breast cancer cells in vitro
Source: BMC Cancer. 2022 Jun 27;22:707. doi: 10.1186/s12885-022-09782-z (PMC9238053; doi:10.1186/s12885-022-09782-z)

BT-549

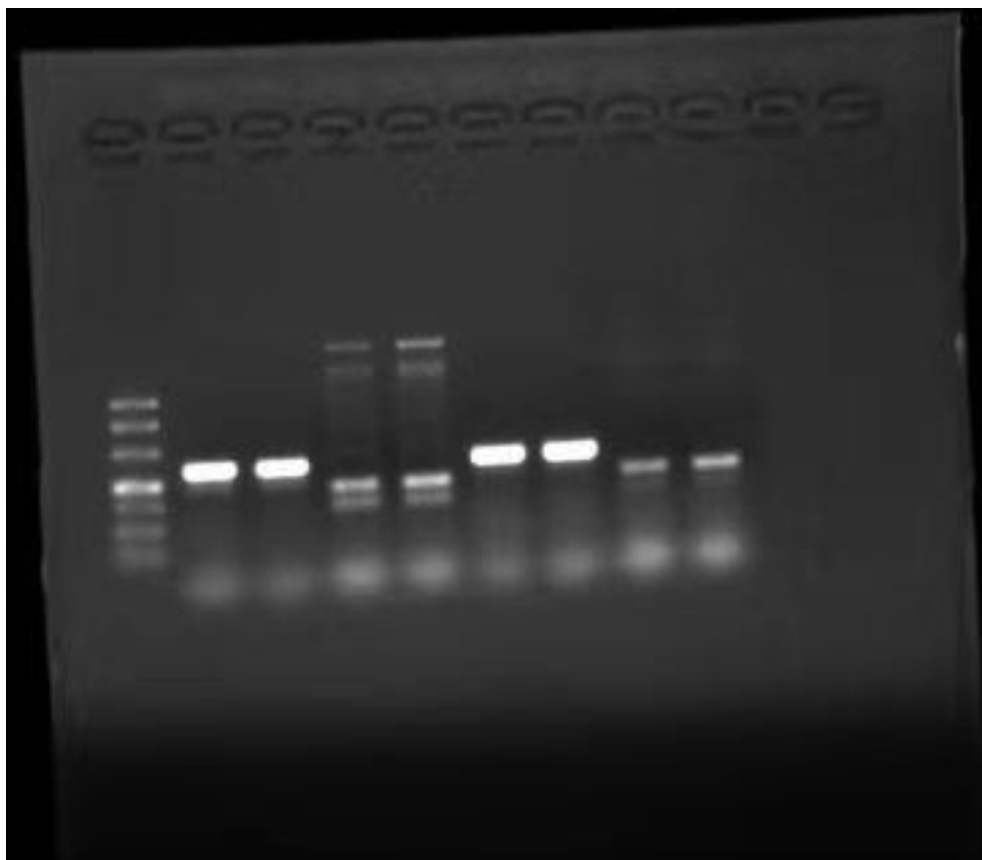

MDA-MB-231

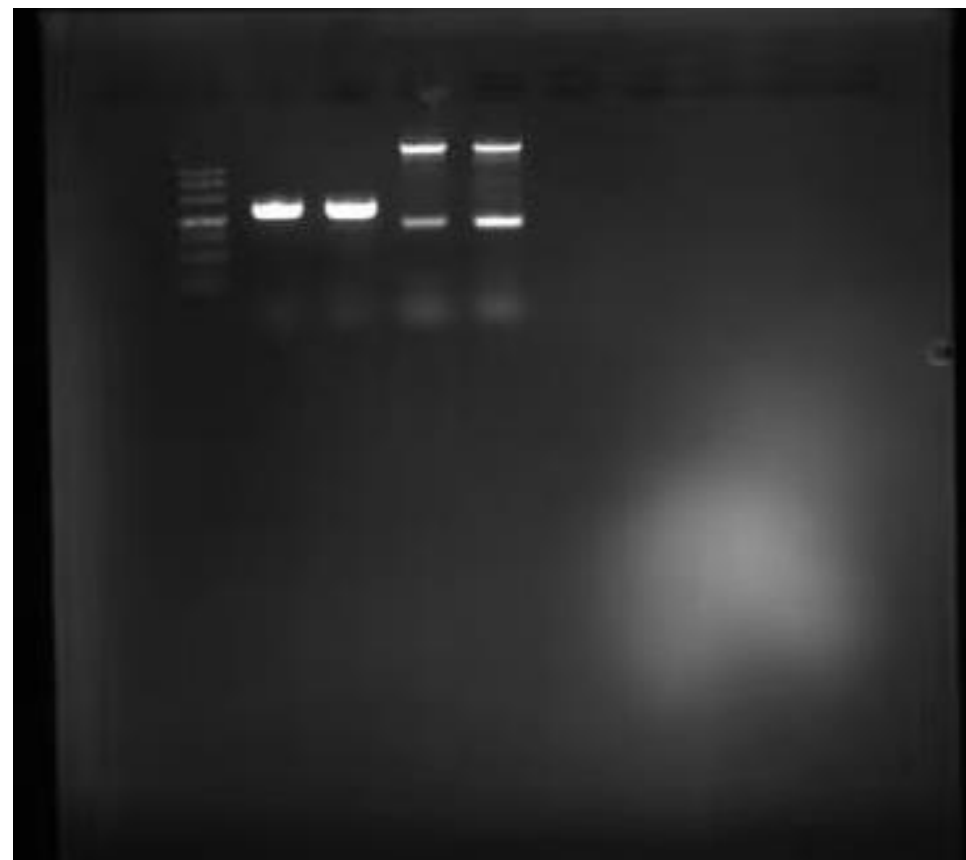

Supplement: Supplementary file 1 — Additional file 1. [file 12885_2022_9782_MOESM1_ESM.pdf]

Fig 4B- BT549-  $\beta$ -actin

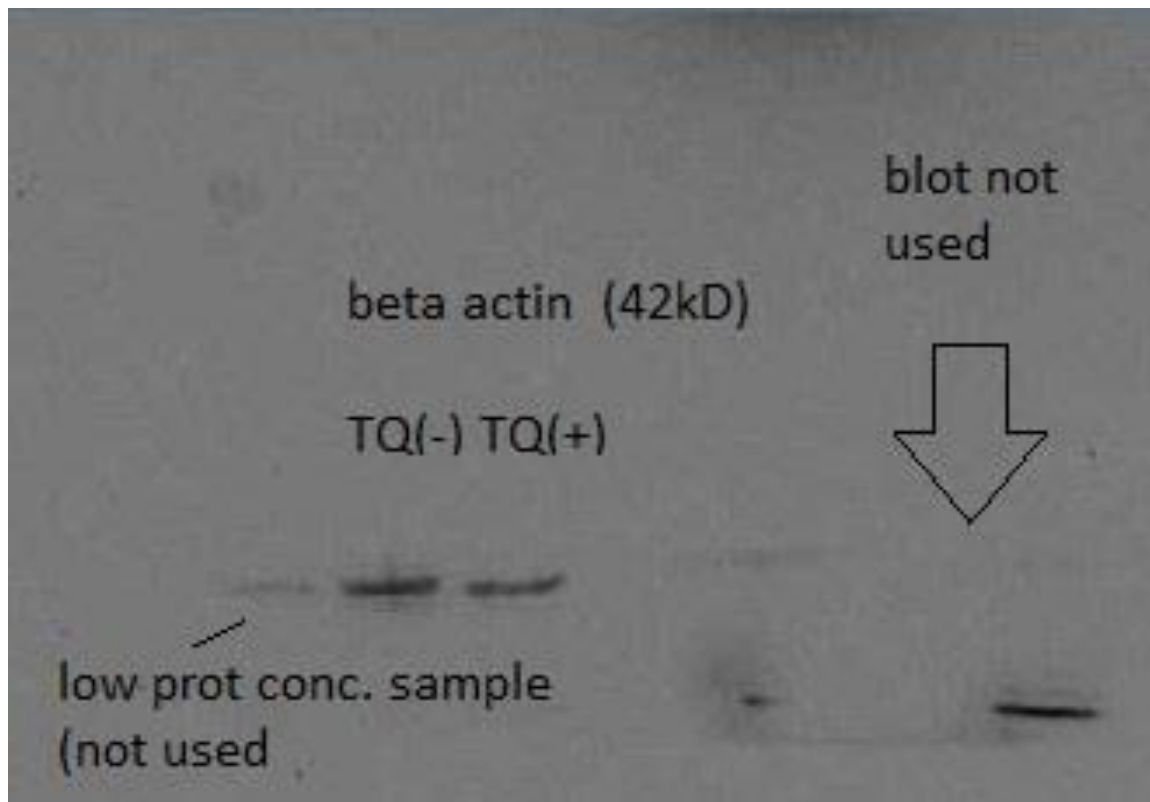

Fig 4B- BT549-IL17RD

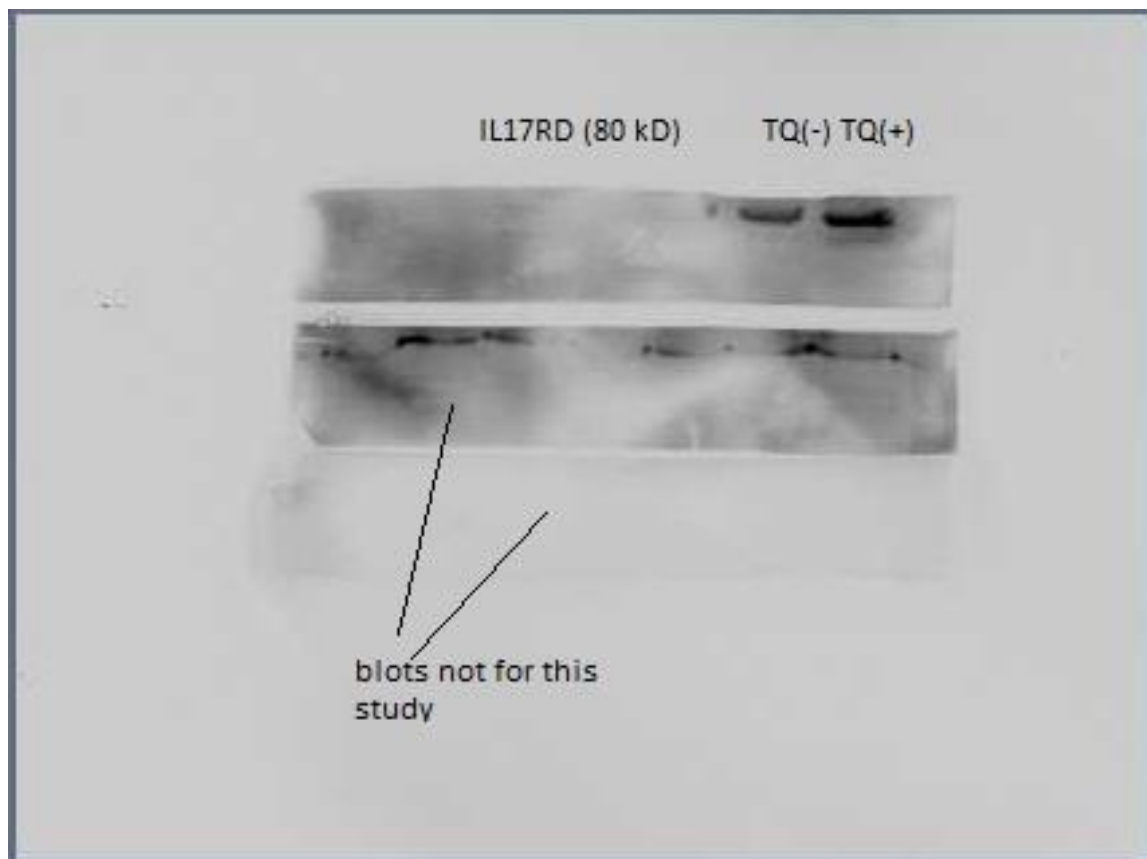

Fig 4B- MDA-MB-231-  $\beta$ -actin

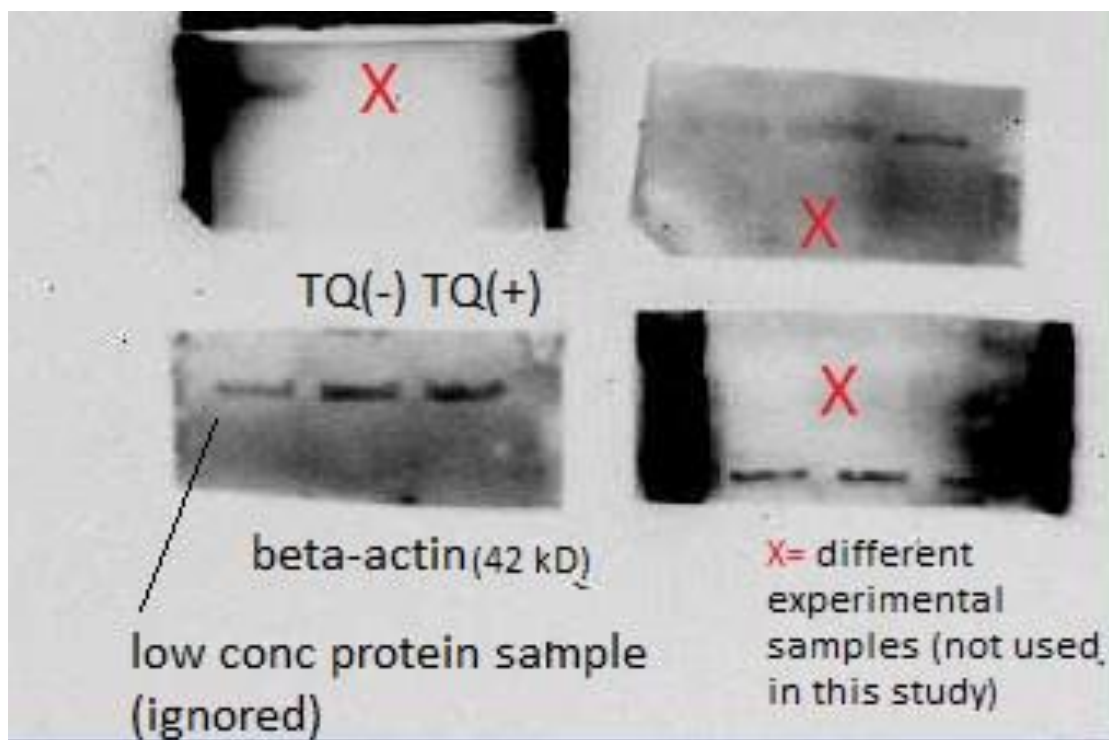

Fig 4B- MDA-MB-231 IL17RD

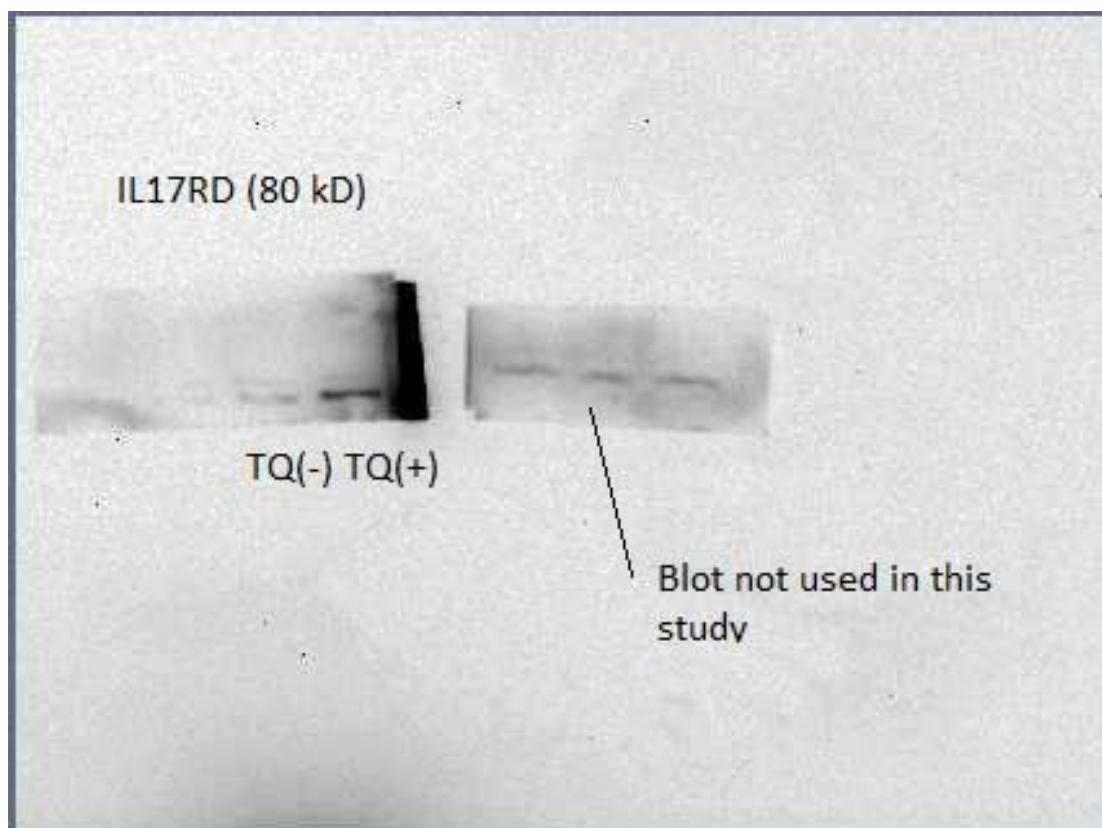

Supplement: Supplementary file 2 — Additional file 2. [file 12885_2022_9782_MOESM2_ESM.pdf]

Raw figure for efficiency of il17rd overexpression or knockdown

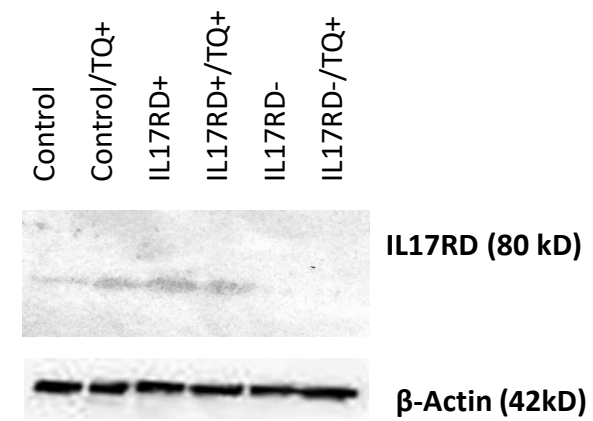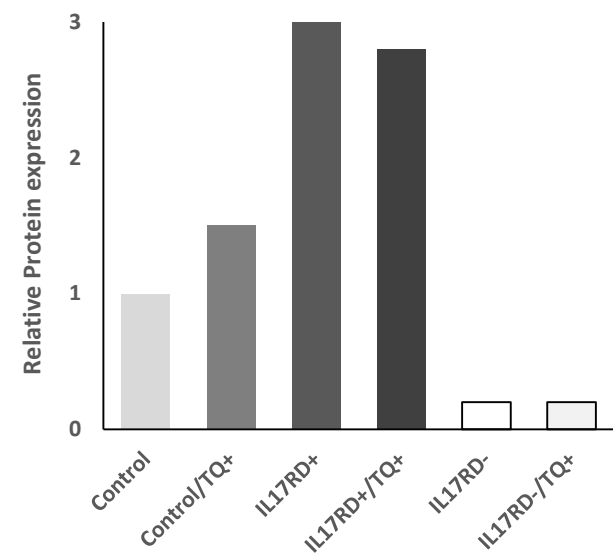

Supplement: Supplementary file 3 — Additional file 3. [file 12885_2022_9782_MOESM3_ESM.pdf]
