# Supplementary material for: Thymoquinone upregulates IL17RD in controlling the growth and metastasis of triple negative breast cancer cells in vitro
Source: BMC Cancer. 2022 Jun 27;22:707. doi: 10.1186/s12885-022-09782-z (PMC9238053; doi:10.1186/s12885-022-09782-z)
Supplement: Supplementary file 4 — Additional file 4: Table S1. Methylated peaks chr. [file 12885_2022_9782_MOESM4_ESM.pdf]

Table S1: Methylated peaks

chr (chromosome)	start	end	length	summit	pvalue	FDR(%)
chr1	4028058	4028249	192	96	0.000	76.84
chr1	8197124	8197364	241	80	0.000	67.17
chr1	9401001	9401328	328	252	0.000	59.72
chr1	14059647	14059781	135	67	0.000	75.72
chr1	15352246	15352458	213	106	0.000	73.76
chr1	16602551	16602811	261	62	0.000	71.32
chr1	16691626	16691823	198	99	0.000	63.33
chr1	17763051	17763308	258	117	0.000	54.55
chr1	17845850	17845986	137	68	0.000	32.46
chr1	17928976	17929140	165	82	0.000	45.33
chr1	20270927	20271142	216	108	0.000	70.66
chr1	20703153	20703311	159	79	0.000	71.61
chr1	21864363	21864702	340	87	0.000	70.72
chr1	22114061	22114281	221	110	0.000	75
chr1	23729672	23729862	191	95	0.000	74.52
chr1	25548579	25548806	228	114	0.000	75
chr1	29095319	29095543	225	112	0.000	68.37
chr1	29656814	29657106	293	73	0.000	71.28
chr1	30621181	30621548	368	190	0.000	62.24
chr1	31998740	31998977	238	91	0.000	63.33
chr1	32363352	32363567	216	108	0.000	70.66
chr1	36529151	36529326	176	88	0.000	32.56
chr1	36809303	36809487	185	92	0.000	62.61
chr1	37375210	37375334	125	62	0.000	26.67
chr1	38060458	38060581	124	62	0.000	31.82
chr1	39715075	39715263	189	94	0.000	74.52
chr1	44627399	44627591	193	96	0.000	64.55
chr1	47198517	47198660	144	72	0.000	28.46
chr1	48286938	48287130	193	96	0.000	64.92
chr1	51171844	51172015	172	86	0.000	61.04
chr1	52876465	52876654	190	95	0.000	64.92
chr1	54453000	54453198	199	99	0.000	61.8
chr1	54596457	54596716	260	106	0.000	75.3
chr1	55081130	55081305	176	88	0.000	74.68
chr1	55609731	55609961	231	115	0.000	75.98
chr1	58539807	58540132	326	144	0.000	71.73
chr1	60455915	60456045	131	65	0.000	45.16
chr1	61259793	61259952	160	80	0.000	46.33
chr1	61415371	61415633	263	61	0.000	74.64
chr1	61955849	61955974	126	63	0.000	72.29
chr1	62140938	62141075	138	69	0.000	32.46
chr1	62768468	62768594	127	63	0.000	27.56
chr1	65189454	65189652	199	99	0.000	71.67
chr1	66096910	66097124	215	107	0.000	45.8
chr1	66805371	66805580	210	105	0.000	71.67
chr1	68189620	68189751	132	66	0.000	34.57
chr1	68663880	68664190	311	217	0.000	61.15
chr1	69243537	69243673	137	68	0.000	75.82
chr1	72179205	72179453	249	76	0.000	73.17
chr1	72570887	72571153	267	112	0.000	67.17
chr1	73044854	73045123	270	117	0.000	71.6
chr1	73979936	73980083	148	74	0.000	70.98
chr1	77417822	77417944	123	61	0.000	38.12
chr1	78556194	78556357	164	82	0.000	62.08
chr1	79761943	79762287	345	283	0.000	54.33
chr1	80636364	80636526	163	81	0.000	54.8

chr1	81897679	81897907	229	114	0.000	74.16
chr1	82645482	82645774	293	96	0.000	47.62
chr1	83070918	83071126	209	104	0.000	70.66
chr1	85784575	85784910	336	101	0.000	67.7
chr1	88085699	88085949	251	108	0.000	61.94
chr1	88258936	88259058	123	61	0.000	38.12
chr1	88756184	88756422	239	123	0.000	62.18
chr1	91446881	91447037	157	78	0.000	66.63
chr1	94343450	94343642	193	96	0.000	47.53
chr1	95407700	95407849	150	75	0.000	76.08
chr1	99597165	99597341	177	88	0.000	63.82
chr1	103419048	103419208	161	80	0.000	72.14
chr1	103592161	103592401	241	112	0.000	75.58
chr1	107291218	107291421	204	102	0.000	72.2
chr1	108359731	108359899	169	84	0.000	54.42
chr1	109968554	109968993	440	113	0.000	66.67
chr1	110739477	110739653	177	88	0.000	72
chr1	112005090	112005237	148	74	0.000	35.76
chr1	113001781	113001939	159	79	0.000	71.61
chr1	114218647	114218783	137	68	0.000	75.82
chr1	116963439	116963713	275	157	0.000	62.22
chr1	118975584	118975878	295	69	0.000	71.26
chr1	120206035	120206219	185	92	0.000	68.45
chr1	121462483	121462652	170	85	0.000	74.19
chr1	145465772	145465919	148	74	0.000	56.29
chr1	146505142	146505299	158	79	0.000	46.33
chr1	147000045	147000205	161	80	0.000	45.33
chr1	147292841	147293342	502	116	0.000	75.53
chr1	148943612	148943961	350	279	0.000	72
chr1	149024602	149024933	332	223	0.000	68
chr1	149359522	149359655	134	67	0.000	54.42
chr1	152488429	152488595	167	83	0.000	64.33
chr1	152596322	152596530	209	104	0.000	70.73
chr1	155659580	155659803	224	112	0.000	61.3
chr1	160749984	160750193	210	105	0.000	73.7
chr1	161521841	161522162	322	111	0.000	60.71
chr1	161821061	161821284	224	112	0.000	63.12
chr1	162745038	162745386	349	65	0.000	62.11
chr1	162750043	162750195	153	76	0.000	56.29
chr1	164640114	164640320	207	103	0.000	73.98
chr1	164765484	164765778	295	124	0.000	75.34
chr1	169443174	169443319	146	73	0.000	64.1
chr1	176077476	176077631	156	78	0.000	33.6
chr1	177619632	177619772	141	70	0.000	32.46
chr1	177809675	177809849	175	87	0.000	60.31
chr1	179188915	179189201	287	110	0.000	63.75
chr1	179319378	179319562	185	92	0.000	63.33
chr1	180424221	180424353	133	66	0.000	32.51
chr1	181371454	181371758	305	183	0.000	71.55
chr1	182407126	182407398	273	100	0.000	63.05
chr1	183802674	183802844	171	85	0.000	60.32
chr1	186583974	186584182	209	104	0.000	71.63
chr1	187389267	187389476	210	105	0.000	68.45
chr1	187722770	187723142	373	75	0.000	73.37
chr1	190958417	190958589	173	86	0.000	32.46
chr1	191781870	191782028	159	79	0.000	45.33
chr1	193988960	193989297	338	65	0.000	63.03
chr1	196509199	196509425	227	113	0.000	76.93
chr1	197923866	197923989	124	62	0.000	34.57
chr1	200409100	200409404	305	114	0.000	64.06

chr1	203019457	203019649	193	96	0.000	47.42
chr1	203169438	203169849	412	130	0.000	72.17
chr1	203479936	203480166	231	115	0.000	74.46
chr1	204013637	204013898	262	105	0.000	46.98
chr1	204099327	204099492	166	83	0.000	35.56
chr1	204275237	204275376	140	70	0.000	64.84
chr1	204866919	204867044	126	63	0.000	74.01
chr1	206598760	206598945	186	93	0.000	75.81
chr1	207306352	207306524	173	86	0.000	35.76
chr1	207377653	207377977	325	116	0.000	66.42
chr1	208889052	208889256	205	102	0.000	75
chr1	211074658	211074894	237	161	0.000	75
chr1	211155563	211155785	223	111	0.000	75
chr1	217165333	217165604	272	69	0.000	53.81
chr1	217227586	217227910	325	96	0.000	61.29
chr1	217372543	217372870	328	63	0.000	59.72
chr1	222815399	222815558	160	80	0.000	36.88
chr1	223657906	223658060	155	77	0.000	33.6
chr1	226452783	226453055	273	61	0.000	70.47
chr1	230065884	230066012	129	64	0.000	76.82
chr1	230566176	230566528	353	235	0.000	64.65
chr1	231605605	231605935	331	91	0.000	65.37
chr1	232712511	232712863	353	96	0.000	63.85
chr1	233837463	233837621	159	79	0.000	57.22
chr1	236221580	236221719	140	70	0.000	54.42
chr1	239663906	239664072	167	83	0.000	74.64
chr1	239902937	239903061	125	62	0.000	31.82
chr1	241468589	241468848	260	89	0.000	74.68
chr1	243096400	243096526	127	63	0.000	72.53
chr1	245879202	245879335	134	67	0.000	45.45
chr1	246773989	246774217	229	114	0.000	76.74
chr1	248048628	248048779	152	76	0.000	33.6
chr10	413396	413582	187	93	0.000	57.22
chr10	3089982	3090147	166	83	0.000	45.33
chr10	7998245	7998481	237	116	0.000	75.02
chr10	8200821	8201127	307	108	0.000	61.55
chr10	8345367	8345534	168	84	0.000	50.57
chr10	8915550	8915731	182	91	0.000	63.33
chr10	9430874	9431005	132	66	0.000	26.67
chr10	11125241	11125546	306	141	0.000	50.78
chr10	11511936	11512238	303	107	0.000	46.82
chr10	12574080	12574319	240	81	0.000	67.93
chr10	14544151	14544384	234	117	0.000	72.62
chr10	15100922	15101108	187	93	0.000	63.33
chr10	17914416	17914607	192	96	0.000	33.6
chr10	18237190	18237344	155	77	0.000	66.36
chr10	19035720	19035892	173	86	0.000	63.13
chr10	22153625	22153826	202	101	0.000	72.11
chr10	22198210	22198632	423	168	0.000	71.91
chr10	23795176	23795368	193	96	0.000	76.78
chr10	26574177	26574377	201	100	0.000	66.35
chr10	26964603	26964900	298	224	0.000	62.99
chr10	27128489	27128748	260	85	0.000	60.82
chr10	28696272	28696399	128	64	0.000	47.5
chr10	28979891	28980019	129	64	0.000	42.42
chr10	31227499	31227626	128	64	0.000	50
chr10	32068958	32069089	132	66	0.000	0
chr10	35528766	35529027	262	91	0.000	72.92
chr10	38013593	38013722	130	65	0.000	0
chr10	42386856	42388795	1940	1530	0.000	62.5

chr10	42480719	42480956	238	60	0.000	70.66
chr10	42540534	42540927	394	235	0.000	70.53
chr10	42665388	42665705	318	176	0.000	74.7
chr10	43922426	43922666	241	173	0.000	74.59
chr10	45570449	45570734	286	171	0.000	44.74
chr10	50130314	50130508	195	97	0.000	35.76
chr10	50383679	50384027	349	106	0.000	71.34
chr10	55380695	55380823	129	64	0.000	26.67
chr10	56512557	56512682	126	63	0.000	34.57
chr10	57419740	57419888	149	74	0.000	33.6
chr10	57515923	57516201	279	67	0.000	69.88
chr10	57572808	57572982	175	87	0.000	69.55
chr10	58983784	58984087	304	85	0.000	56.09
chr10	59892269	59892808	540	152	0.000	65.54
chr10	60768260	60768429	170	85	0.000	45.8
chr10	64458608	64458940	333	253	0.000	75.58
chr10	64785775	64785944	170	85	0.000	56.29
chr10	65978388	65978703	316	99	0.000	61.84
chr10	70324418	70324563	146	73	0.000	71.15
chr10	70682966	70683096	131	65	0.000	70.96
chr10	73553568	73553725	158	79	0.000	57.22
chr10	73900932	73901184	253	175	0.000	64.55
chr10	74815387	74815743	357	284	0.000	63.4
chr10	75544875	75545046	172	86	0.000	56.29
chr10	78335520	78335847	328	232	0.000	59.72
chr10	79463199	79463485	287	71	0.000	47.55
chr10	83213426	83213577	152	76	0.000	50.58
chr10	83600160	83600284	125	62	0.000	26.67
chr10	84503853	84504262	410	215	0.000	51.71
chr10	88001753	88001933	181	90	0.000	63.33
chr10	88810021	88810172	152	76	0.000	65.49
chr10	89540404	89540595	192	96	0.000	71.19
chr10	90670519	90670697	179	89	0.000	64.92
chr10	90815493	90815685	193	96	0.000	76.75
chr10	91043724	91043990	267	158	0.000	63.33
chr10	92417541	92417701	161	80	0.000	75.15
chr10	93156194	93156381	188	94	0.000	68.45
chr10	93228108	93228254	147	73	0.000	32.54
chr10	94678792	94678972	181	90	0.000	74.52
chr10	95030711	95031031	321	250	0.000	68.68
chr10	95918038	95918175	138	69	0.000	76.07
chr10	98279683	98279916	234	117	0.000	62.61
chr10	98410173	98410374	202	101	0.000	72.11
chr10	100000889	100001015	127	63	0.000	34.57
chr10	101378096	101378243	148	74	0.000	70.98
chr10	109677353	109677567	215	107	0.000	71.92
chr10	114706193	114706399	207	103	0.000	71.77
chr10	115256313	115256558	246	167	0.000	73.2
chr10	116035253	116035431	179	89	0.000	77.08
chr10	117177837	117178004	168	84	0.000	73.93
chr10	119185815	119185991	177	88	0.000	45.95
chr10	119376923	119377272	350	235	0.000	61.86
chr10	121549542	121549788	247	117	0.000	67.17
chr10	122577467	122577646	180	90	0.000	76.22
chr10	123515650	123516031	382	104	0.000	67.17
chr10	124440985	124441250	266	167	0.000	70.63
chr10	127224224	127224374	151	75	0.000	45.33
chr10	127593964	127594218	255	75	0.000	71.25
chr10	130269810	130269994	185	92	0.000	75.79
chr10	131378858	131379022	165	82	0.000	33.6

chr11	615323	615731	409	253	0.000	72.28
chr11	1473808	1474153	346	62	0.000	62.53
chr11	7173813	7174059	247	98	0.000	71.71
chr11	8941480	8941661	182	91	0.000	64.92
chr11	13268882	13269104	223	111	0.000	63.33
chr11	14709633	14709854	222	111	0.000	70.66
chr11	14989016	14989153	138	69	0.000	76.07
chr11	15353315	15353523	209	104	0.000	73.18
chr11	17142157	17142437	281	220	0.000	71.1
chr11	18603808	18603930	123	61	0.000	71.59
chr11	19610520	19610642	123	61	0.000	26.67
chr11	20604465	20604590	126	63	0.000	26.67
chr11	20615061	20615261	201	100	0.000	63.11
chr11	29434526	29434684	159	79	0.000	45.95
chr11	32712977	32713105	129	64	0.000	34.57
chr11	39349882	39350265	384	109	0.000	71.71
chr11	39929299	39929525	227	113	0.000	64.16
chr11	41708463	41708687	225	112	0.000	32.51
chr11	45287390	45287542	153	76	0.000	45.33
chr11	45531486	45531688	203	101	0.000	75
chr11	47757785	47758003	219	109	0.000	75
chr11	48817849	48818088	240	122	0.000	66.02
chr11	50712787	50713105	319	157	0.000	71.34
chr11	51579921	51580690	770	603	0.000	75.48
chr11	57222822	57223059	238	114	0.000	75.58
chr11	58577337	58577618	282	86	0.000	75.58
chr11	64805308	64805618	311	77	0.000	67.27
chr11	66533717	66533928	212	106	0.000	75
chr11	67010511	67010663	153	76	0.000	33.6
chr11	69907127	69907431	305	110	0.000	64.73
chr11	71564726	71564963	238	162	0.000	75
chr11	71656315	71656613	299	82	0.000	60.08
chr11	80264597	80264719	123	61	0.000	38.12
chr11	80877009	80877349	341	96	0.000	67.4
chr11	84418831	84419083	253	143	0.000	71.67
chr11	85466438	85466750	313	63	0.000	60.88
chr11	86888729	86888860	132	66	0.000	34.57
chr11	90047006	90047282	277	146	0.000	66.02
chr11	95003820	95004069	250	101	0.000	66.26
chr11	100172881	100173064	184	92	0.000	63.88
chr11	100512218	100512362	145	72	0.000	71.87
chr11	104510909	104511046	138	69	0.000	46.67
chr11	114535947	114536138	192	96	0.000	53.89
chr11	119154418	119154724	307	78	0.000	61.55
chr11	121582882	121583070	189	94	0.000	57.22
chr11	122145415	122145579	165	82	0.000	73.74
chr11	122793964	122794147	184	92	0.000	62.61
chr11	124362233	124362433	201	100	0.000	71.56
chr11	125585117	125585457	341	115	0.000	60.42
chr11	127339482	127339821	340	170	0.000	72.28
chr11	127818404	127818544	141	70	0.000	63.59
chr11	131398700	131398968	269	116	0.000	75.37
chr11	132324581	132324814	234	117	0.000	73.09
chr12	1725283	1725443	161	80	0.000	72.14
chr12	8631479	8631702	224	112	0.000	63.12
chr12	11285060	11285195	136	68	0.000	63.12
chr12	11597510	11597899	390	289	0.000	64.31
chr12	13848854	13849078	225	112	0.000	75
chr12	15570841	15571096	256	117	0.000	71.45
chr12	16229566	16229756	191	95	0.000	53.83

chr12	20392106	20392291	186	93	0.000	45.33
chr12	23536494	23536799	306	114	0.000	76.62
chr12	29788841	29789007	167	83	0.000	45.33
chr12	32285959	32286160	202	101	0.000	72.11
chr12	33120409	33120551	143	71	0.000	76.53
chr12	38167379	38167715	337	65	0.000	63.5
chr12	38248542	38248755	214	107	0.000	70.66
chr12	42729185	42729352	168	84	0.000	76.46
chr12	44063847	44064098	252	117	0.000	71.93
chr12	44298406	44298563	158	79	0.000	57.22
chr12	52151996	52152288	293	226	0.000	62.21
chr12	52772046	52772342	297	117	0.000	62.77
chr12	53570796	53571015	220	110	0.000	75
chr12	54082694	54083114	421	239	0.000	65.05
chr12	54672308	54672464	157	78	0.000	33.6
chr12	55081870	55082032	163	81	0.000	71.74
chr12	56979451	56979745	295	60	0.000	63.1
chr12	58908083	58908249	167	83	0.000	60.96
chr12	61546713	61546941	229	114	0.000	62.45
chr12	67446017	67446169	153	76	0.000	65.93
chr12	68205363	68205542	180	90	0.000	55.56
chr12	69033374	69033496	123	61	0.000	26.67
chr12	69627108	69627431	324	100	0.000	68.42
chr12	69857484	69857801	318	125	0.000	27.34
chr12	72060979	72061115	137	68	0.000	35.76
chr12	76773253	76773398	146	73	0.000	35.76
chr12	79130839	79130991	153	76	0.000	65.93
chr12	82446386	82446595	210	105	0.000	71.82
chr12	83349562	83349759	198	99	0.000	33.6
chr12	86706604	86706907	304	213	0.000	75.58
chr12	87997218	87997366	149	74	0.000	33.6
chr12	88448528	88448682	155	77	0.000	66.36
chr12	88903718	88903955	238	106	0.000	53.81
chr12	90478162	90478317	156	78	0.000	63.23
chr12	91177173	91177339	167	83	0.000	52.17
chr12	92604612	92604948	337	61	0.000	63.5
chr12	98446280	98446458	179	89	0.000	36.88
chr12	100656128	100656337	210	105	0.000	68.45
chr12	102460084	102460266	183	91	0.000	64.92
chr12	103084817	103084995	179	89	0.000	75.83
chr12	103406600	103406722	123	61	0.000	71.59
chr12	103808576	103808734	159	79	0.000	54.42
chr12	105037387	105037525	139	69	0.000	76.22
chr12	110119932	110120064	133	66	0.000	32.46
chr12	117146414	117146655	242	127	0.000	74.14
chr12	118325593	118325761	169	84	0.000	73.92
chr12	123397838	123398221	384	126	0.000	76.15
chr12	124563166	124563373	208	104	0.000	74.22
chr12	126276406	126276681	276	202	0.000	69.76
chr12	130417623	130417935	313	101	0.000	69.9
chr12	130534760	130534928	169	84	0.000	54.42
chr12	131303940	131304137	198	99	0.000	68.45
chr13	23801223	23801408	186	93	0.000	60.98
chr13	24436369	24436535	167	83	0.000	70.29
chr13	28349354	28349476	123	61	0.000	26.67
chr13	28684099	28684279	181	90	0.000	71.81
chr13	29912261	29912615	355	271	0.000	61.17
chr13	30772476	30772755	280	166	0.000	71.71
chr13	30959430	30959606	177	88	0.000	75.83
chr13	31248044	31248328	285	113	0.000	75.58

chr13	31325690	31325945	256	115	0.000	71.55
chr13	33460285	33460486	202	101	0.000	76.93
chr13	35814181	35814512	332	110	0.000	69.7
chr13	36840533	36840736	204	102	0.000	74.86
chr13	37527360	37527503	144	72	0.000	72.86
chr13	37887967	37888239	273	199	0.000	69.95
chr13	39048255	39048420	166	83	0.000	57.22
chr13	41410424	41410570	147	73	0.000	65.6
chr13	43489142	43489570	429	247	0.000	75.58
chr13	43713459	43713683	225	112	0.000	72.62
chr13	45214811	45215057	247	93	0.000	75.58
chr13	45341446	45341583	138	69	0.000	76.07
chr13	45865209	45865406	198	99	0.000	71.33
chr13	49673279	49673584	306	208	0.000	61.8
chr13	50209145	50209313	169	84	0.000	32.46
chr13	53713970	53714133	164	82	0.000	45.6
chr13	62876549	62876803	255	66	0.000	75.58
chr13	63551984	63552112	129	64	0.000	34.57
chr13	63697365	63697499	135	67	0.000	35.76
chr13	63949665	63949790	126	63	0.000	26.67
chr13	65061299	65061487	189	94	0.000	76.83
chr13	67868766	67868949	184	92	0.000	63.33
chr13	67985376	67985595	220	110	0.000	63
chr13	68565525	68565728	204	102	0.000	72.2
chr13	70030542	70030691	150	75	0.000	57.22
chr13	73254931	73255169	239	124	0.000	70.09
chr13	74012691	74012897	207	103	0.000	73.7
chr13	74054063	74054268	206	103	0.000	74.75
chr13	74744567	74744860	294	224	0.000	73.8
chr13	76763349	76763478	130	65	0.000	26.67
chr13	87309035	87309283	249	115	0.000	61.83
chr13	87385589	87385843	255	192	0.000	74.75
chr13	87585516	87585679	164	82	0.000	57.22
chr13	87977453	87977630	178	89	0.000	64.56
chr13	89443782	89443992	211	105	0.000	64.05
chr13	91168248	91168409	162	81	0.000	32.46
chr13	92675178	92675481	304	98	0.000	47.27
chr13	93957792	93957941	150	75	0.000	38.12
chr13	94072068	94072360	293	123	0.000	66.27
chr13	95114665	95114985	321	228	0.000	68.68
chr13	96460542	96460705	164	82	0.000	32.51
chr13	98078436	98078617	182	91	0.000	75.75
chr13	98695755	98696031	277	83	0.000	61.46
chr13	99269269	99269452	184	92	0.000	75.34
chr13	101580851	101581150	300	155	0.000	59.96
chr13	102462924	102463079	156	78	0.000	26.67
chr13	103318881	103319149	269	171	0.000	73.81
chr13	109182573	109182918	346	233	0.000	62.53
chr13	109810079	109810253	175	87	0.000	75.14
chr13	110565072	110565293	222	111	0.000	70.66
chr14	20608921	20609238	318	220	0.000	75.67
chr14	20619312	20619536	225	112	0.000	33.01
chr14	20664520	20664717	198	99	0.000	67.16
chr14	22341249	22341438	190	95	0.000	76.84
chr14	26104462	26104863	402	99	0.000	50.77
chr14	26295050	26295254	205	102	0.000	72.33
chr14	29348474	29348803	330	216	0.000	67.17
chr14	34246133	34246326	194	97	0.000	75.24
chr14	37700651	37700793	143	71	0.000	72.92
chr14	44441008	44441165	158	79	0.000	34.57

chr14	44956248	44956375	128	64	0.000	38.12
chr14	45636259	45636433	175	87	0.000	63.91
chr14	46027415	46027667	253	177	0.000	70.82
chr14	50249841	50250169	329	250	0.000	63.68
chr14	50902315	50902581	267	177	0.000	70.62
chr14	52572390	52572518	129	64	0.000	26.67
chr14	56518004	56518340	337	217	0.000	74.41
chr14	57251326	57251576	251	151	0.000	71.43
chr14	57733265	57733501	237	119	0.000	71.46
chr14	63919814	63920092	279	99	0.000	75.58
chr14	66600343	66600465	123	61	0.000	71.59
chr14	67765252	67765594	343	279	0.000	66.83
chr14	68699551	68699834	284	98	0.000	67.17
chr14	69464961	69465138	178	89	0.000	47.78
chr14	71474455	71474622	168	84	0.000	32.46
chr14	75652916	75653067	152	76	0.000	66.63
chr14	79863160	79863447	288	176	0.000	71.37
chr14	81423770	81423943	174	87	0.000	74.59
chr14	82668472	82668630	159	79	0.000	57.22
chr14	83539711	83540079	369	256	0.000	76.43
chr14	85879672	85879903	232	116	0.000	70.21
chr14	86867054	86867462	409	99	0.000	76.75
chr14	87367378	87367661	284	72	0.000	76.71
chr14	89386159	89386387	229	114	0.000	76.87
chr14	90522780	90523024	245	155	0.000	73.41
chr14	93562928	93563104	177	88	0.000	63.33
chr14	96753102	96753236	135	67	0.000	32.46
chr14	96863465	96863617	153	76	0.000	45.33
chr14	101717504	101717788	285	191	0.000	62.39
chr14	103982522	103982820	299	75	0.000	75.84
chr14	106573359	106573480	122	61	0.000	71.82
chr14	106996302	106996444	143	71	0.000	35.56
chr15	20487256	20487404	149	74	0.000	38.12
chr15	25776285	25776509	225	112	0.000	45.33
chr15	28005098	28005459	362	177	0.000	60.77
chr15	31535897	31536179	283	109	0.000	71.41
chr15	31536620	31536808	189	94	0.000	76.83
chr15	35877015	35877341	327	107	0.000	73.93
chr15	38485944	38486216	273	69	0.000	75.43
chr15	43771635	43771790	156	78	0.000	66.51
chr15	44108675	44108947	273	65	0.000	70.47
chr15	44185249	44185587	339	96	0.000	64.44
chr15	46125025	46125303	279	89	0.000	69.14
chr15	47031352	47031508	157	78	0.000	66.79
chr15	48836634	48836936	303	152	0.000	76.41
chr15	54865872	54866034	163	81	0.000	32.46
chr15	55291917	55292379	463	112	0.000	74.84
chr15	56466970	56467317	348	60	0.000	62.2
chr15	59312425	59312662	238	83	0.000	75
chr15	61416296	61416528	233	116	0.000	70.66
chr15	66145394	66145661	268	187	0.000	70.48
chr15	66436690	66436845	156	78	0.000	50.58
chr15	73140430	73140564	135	67	0.000	32.46
chr15	73986879	73987013	135	67	0.000	45.95
chr15	74050391	74050630	240	117	0.000	67.57
chr15	83050004	83050217	214	107	0.000	68.45
chr15	83646294	83646420	127	63	0.000	28.46
chr15	85679199	85679398	200	100	0.000	75
chr15	88291473	88291814	342	273	0.000	63.13
chr15	89157030	89157202	173	86	0.000	76.39

chr15	91087248	91087521	274	117	0.000	76.2
chr15	91498858	91499040	183	91	0.000	77.04
chr15	93839974	93840228	255	71	0.000	71.97
chr15	95090948	95091116	169	84	0.000	61.36
chr15	100207757	100207897	141	70	0.000	76.21
chr16	1004947	1005118	172	86	0.000	63.33
chr16	3208890	3209127	238	92	0.000	71.93
chr16	3222025	3222206	182	91	0.000	61.32
chr16	3806729	3807159	431	138	0.000	55.89
chr16	5450608	5450865	258	195	0.000	73.12
chr16	7441660	7441839	180	90	0.000	47.59
chr16	8368387	8368511	125	62	0.000	34.57
chr16	9641893	9642210	318	78	0.000	69.47
chr16	10080248	10080410	163	81	0.000	33.6
chr16	11264467	11264805	339	252	0.000	67.87
chr16	12734879	12735273	395	114	0.000	72.97
chr16	12895988	12896166	179	89	0.000	64.6
chr16	14584811	14584937	127	63	0.000	26.67
chr16	16122053	16122377	325	261	0.000	47.56
chr16	16640993	16641224	232	116	0.000	63.9
chr16	16772883	16773213	331	219	0.000	63.62
chr16	24064434	24064607	174	87	0.000	74.59
chr16	25335608	25335764	157	78	0.000	46.33
chr16	29294568	29294784	217	108	0.000	68.45
chr16	31155400	31155712	313	251	0.000	69.9
chr16	35144617	35145043	427	111	0.000	71.91
chr16	35215927	35216096	170	85	0.000	61.99
chr16	46403354	46409281	5928	365	0.000	71.28
chr16	46426663	46429481	2819	389	0.000	63.83
chr16	47986964	47987146	183	91	0.000	76.19
chr16	48469489	48469823	335	74	0.000	67.67
chr16	51813339	51813536	198	99	0.000	69.85
chr16	53301618	53301775	158	79	0.000	26.67
chr16	54795280	54795408	129	64	0.000	34.57
chr16	56353886	56354146	261	92	0.000	71.32
chr16	57056179	57056312	134	67	0.000	62.05
chr16	57379498	57380014	517	296	0.000	72.79
chr16	57632640	57632934	295	234	0.000	63.1
chr16	59053808	59053986	179	89	0.000	56.29
chr16	59560679	59560956	278	163	0.000	32.12
chr16	60333664	60333800	137	68	0.000	35.76
chr16	63122199	63122526	328	169	0.000	74.6
chr16	72005057	72005314	258	174	0.000	72.01
chr16	72082219	72082405	187	93	0.000	71.06
chr16	72404538	72404682	145	72	0.000	63.95
chr16	74990323	74990620	298	130	0.000	72.24
chr16	75122661	75122824	164	82	0.000	35.76
chr16	79747549	79747922	374	96	0.000	71.93
chr16	81124417	81124553	137	68	0.000	63.7
chr16	84100897	84101056	160	80	0.000	75.87
chr16	87149467	87149811	345	282	0.000	66.98
chr17	199600	199937	338	272	0.000	60.27
chr17	2625155	2625359	205	102	0.000	32.46
chr17	11744868	11745159	292	115	0.000	67.23
chr17	12468716	12469022	307	229	0.000	70.52
chr17	12793120	12793533	414	215	0.000	70.86
chr17	14646210	14646581	372	294	0.000	73.81
chr17	15304848	15304981	134	67	0.000	73.27
chr17	21170717	21170993	277	111	0.000	61.46
chr17	21906618	21906756	139	69	0.000	73.7

chr17	26566297	26566616	320	237	0.000	69.01
chr17	27012806	27013017	212	106	0.000	72.62
chr17	27086968	27087100	133	66	0.000	54.42
chr17	28136310	28136572	263	159	0.000	65.1
chr17	28190052	28190176	125	62	0.000	52.17
chr17	29083121	29083404	284	202	0.000	64.7
chr17	30222427	30222643	217	108	0.000	60.44
chr17	30487896	30488045	150	75	0.000	57.22
chr17	32473636	32473840	205	102	0.000	66.76
chr17	32598133	32598460	328	179	0.000	76.15
chr17	33711668	33711935	268	90	0.000	64.2
chr17	33970521	33970785	265	168	0.000	76.2
chr17	34534273	34534434	162	81	0.000	74.74
chr17	35389403	35389585	183	91	0.000	75.83
chr17	41229053	41229295	243	164	0.000	67.76
chr17	42113713	42113934	222	111	0.000	62.61
chr17	45468030	45468260	231	115	0.000	45.33
chr17	50618735	50618911	177	88	0.000	63.33
chr17	50983529	50983722	194	97	0.000	71.5
chr17	51341063	51341267	205	102	0.000	75
chr17	52254795	52254922	128	64	0.000	45.21
chr17	53408689	53408842	154	77	0.000	45.33
chr17	55394514	55394764	251	117	0.000	68.52
chr17	60446531	60446698	168	84	0.000	61.09
chr17	64615351	64615575	225	112	0.000	33.6
chr17	66955036	66955196	161	80	0.000	76.79
chr17	68036162	68036514	353	291	0.000	66.23
chr17	68371241	68371484	244	152	0.000	71.93
chr17	68987763	68987910	148	74	0.000	70.98
chr17	69880651	69880852	202	101	0.000	75
chr17	70926524	70926658	135	67	0.000	32.46
chr17	73620594	73620731	138	69	0.000	63.42
chr17	73623344	73623579	236	117	0.000	71.1
chr17	74163117	74163402	286	99	0.000	75.83
chr17	76416804	76416951	148	74	0.000	34.57
chr17	77232977	77233186	210	105	0.000	65.48
chr17	78081093	78081270	178	89	0.000	38.12
chr17	78415834	78415961	128	64	0.000	73.48
chr18	2610326	2610504	179	89	0.000	68.45
chr18	3689080	3689206	127	63	0.000	53.89
chr18	5624886	5625098	213	106	0.000	67.84
chr18	7905996	7906205	210	105	0.000	70.73
chr18	8735157	8735484	328	63	0.000	63.78
chr18	8784160	8784511	352	202	0.000	72.26
chr18	9107666	9107866	201	100	0.000	74.52
chr18	10334093	10334535	443	122	0.000	76.17
chr18	11480047	11480231	185	92	0.000	61.7
chr18	19030056	19030318	263	165	0.000	75.58
chr18	20132803	20132937	135	67	0.000	63.98
chr18	20640893	20641224	332	271	0.000	59.49
chr18	23246108	23246235	128	64	0.000	73.48
chr18	24965596	24965737	142	71	0.000	28.57
chr18	26334490	26334625	136	68	0.000	76.33
chr18	26433947	26434123	177	88	0.000	63.33
chr18	34222839	34223013	175	87	0.000	75.14
chr18	37047640	37047935	296	153	0.000	74.39
chr18	38218781	38219014	234	117	0.000	74.54
chr18	39378286	39378460	175	87	0.000	67.16
chr18	48137615	48137792	178	89	0.000	64.56
chr18	51802235	51802572	338	175	0.000	75.58

chr18	53266181	53266371	191	95	0.000	76.88
chr18	61884185	61884509	325	100	0.000	37.95
chr18	65426671	65426866	196	98	0.000	71.36
chr18	66075428	66075656	229	114	0.000	63.15
chr18	67227138	67227456	319	217	0.000	71.77
chr18	69449757	69449891	135	67	0.000	75.72
chr18	69579182	69579319	138	69	0.000	54.42
chr18	71083988	71084357	370	115	0.000	64.06
chr18	72984770	72985058	289	107	0.000	76.75
chr19	1588302	1588430	129	64	0.000	26.67
chr19	9565387	9565636	250	141	0.000	75.39
chr19	11931416	11931619	204	102	0.000	33.6
chr19	13715068	13715282	215	107	0.000	75.86
chr19	14521532	14521714	183	91	0.000	68.45
chr19	16914666	16914869	204	102	0.000	45.33
chr19	19345218	19345428	211	105	0.000	63.06
chr19	20877252	20877654	403	205	0.000	71.73
chr19	20888523	20888731	209	104	0.000	73.18
chr19	24575502	24575927	426	214	0.000	75.89
chr19	27792765	27792958	194	97	0.000	64.47
chr19	27823805	27824020	216	108	0.000	72.62
chr19	28100843	28101004	162	81	0.000	57.22
chr19	28141299	28141441	143	71	0.000	45.8
chr19	30174197	30174319	123	61	0.000	64.71
chr19	32059575	32059726	152	76	0.000	34.57
chr19	37434420	37434640	221	110	0.000	63.36
chr19	38185930	38186137	208	104	0.000	63.33
chr19	42655345	42655521	177	88	0.000	76.58
chr19	44915541	44915800	260	110	0.000	67.17
chr19	46176201	46176368	168	84	0.000	47.71
chr19	46896371	46896511	141	70	0.000	32.46
chr19	50276183	50276400	218	109	0.000	62.31
chr19	51709888	51710018	131	65	0.000	34.57
chr19	52650067	52650194	128	64	0.000	28.57
chr19	54190899	54191218	320	105	0.000	69.01
chr19	55493309	55493656	348	282	0.000	64.41
chr19	55911180	55911406	227	113	0.000	68.45
chr19	57123647	57123822	176	88	0.000	32.46
chr19	58065861	58066051	191	95	0.000	76.88
chr19	58610041	58610394	354	269	0.000	55.69
chr19	58649802	58649998	197	98	0.000	64.92
chr2	682949	683142	194	97	0.000	70.91
chr2	3948978	3949347	370	262	0.000	74.03
chr2	4609789	4610067	279	181	0.000	62.86
chr2	7248713	7249009	297	92	0.000	71.29
chr2	7607105	7607330	226	113	0.000	64.92
chr2	8701748	8701959	212	106	0.000	70.66
chr2	9600581	9600948	368	259	0.000	45.98
chr2	10819852	10820080	229	114	0.000	33.6
chr2	11377801	11377988	188	94	0.000	71.32
chr2	15624077	15624256	180	90	0.000	76.37
chr2	16263051	16263229	179	89	0.000	75.87
chr2	18597690	18597892	203	101	0.000	72.04
chr2	19405608	19405791	184	92	0.000	64.92
chr2	22492131	22492274	144	72	0.000	76.7
chr2	23411808	23411955	148	74	0.000	70.98
chr2	26571220	26571388	169	84	0.000	33.6
chr2	27732760	27732905	146	73	0.000	71.15
chr2	28915473	28915649	177	88	0.000	64.92
chr2	29817897	29818098	202	101	0.000	66.7

chr2	33023936	33024280	345	265	0.000	62.51
chr2	38561915	38562088	174	87	0.000	68.45
chr2	41147857	41148064	208	104	0.000	67.47
chr2	45243207	45243459	253	149	0.000	65.94
chr2	49985678	49985807	130	65	0.000	73.79
chr2	50230482	50230824	343	127	0.000	62.6
chr2	52149014	52149339	326	60	0.000	68.41
chr2	52948991	52949207	217	108	0.000	74.89
chr2	57585635	57585800	166	83	0.000	73.41
chr2	57714431	57714606	176	88	0.000	68.45
chr2	58126623	58126751	129	64	0.000	31.82
chr2	59276001	59276322	322	122	0.000	71.38
chr2	60879984	60880117	134	67	0.000	35.76
chr2	66147045	66147392	348	87	0.000	62.2
chr2	74178916	74179153	238	78	0.000	70.66
chr2	75219814	75219996	183	91	0.000	68.45
chr2	78231886	78232231	346	250	0.000	76.38
chr2	79211278	79211454	177	88	0.000	76.58
chr2	87109354	87109506	153	76	0.000	66.63
chr2	87782302	87782508	207	103	0.000	76.24
chr2	89848606	89848896	291	105	0.000	76.69
chr2	91817087	91817357	271	195	0.000	61.95
chr2	92283453	92283623	171	85	0.000	73.87
chr2	92290002	92291760	1759	1499	0.000	64.22
chr2	92317132	92322247	5116	4680	0.000	72.05
chr2	97002480	97002631	152	76	0.000	65.49
chr2	98537515	98537736	222	111	0.000	73.42
chr2	100274833	100274973	141	70	0.000	76.21
chr2	101605633	101605755	123	61	0.000	26.67
chr2	102260662	102260793	132	66	0.000	27.13
chr2	103093240	103093534	295	137	0.000	76.12
chr2	107943273	107943476	204	102	0.000	66.54
chr2	116255568	116255718	151	75	0.000	65.31
chr2	116964678	116964965	288	106	0.000	75.58
chr2	117176200	117176332	133	66	0.000	35.76
chr2	121264362	121264507	146	73	0.000	71.15
chr2	123939301	123939655	355	195	0.000	66.76
chr2	132588445	132588569	125	62	0.000	38.12
chr2	132976505	132976662	158	79	0.000	50.18
chr2	137768424	137768660	237	102	0.000	71.48
chr2	140725821	140726131	311	86	0.000	70.12
chr2	143984526	143984721	196	98	0.000	63.33
chr2	146077220	146077486	267	115	0.000	71.34
chr2	146547801	146547988	188	94	0.000	33.6
chr2	147014049	147014296	248	110	0.000	73.44
chr2	148095395	148095604	210	105	0.000	75
chr2	150460116	150460409	294	113	0.000	68.27
chr2	151087302	151087505	204	102	0.000	72.2
chr2	153618918	153619141	224	112	0.000	70.66
chr2	153668834	153668958	125	62	0.000	34.57
chr2	156384368	156384513	146	73	0.000	32.23
chr2	156966098	156966224	127	63	0.000	34.57
chr2	157542092	157542433	342	93	0.000	71.91
chr2	157641574	157641748	175	87	0.000	63.33
chr2	158546836	158547024	189	94	0.000	68.6
chr2	160531639	160531922	284	126	0.000	63.37
chr2	161224909	161225049	141	70	0.000	76.21
chr2	165943953	165944093	141	70	0.000	63.59
chr2	181021732	181021955	224	112	0.000	75
chr2	182195996	182196170	175	87	0.000	68.38

chr2	192737639	192737873	235	117	0.000	70.66
chr2	192777463	192777608	146	73	0.000	64.1
chr2	193943052	193943271	220	110	0.000	61
chr2	195065757	195065932	176	88	0.000	75.95
chr2	199577619	199577762	144	72	0.000	38.12
chr2	201600776	201600976	201	100	0.000	71.56
chr2	202953850	202953973	124	62	0.000	34.57
chr2	205137792	205137939	148	74	0.000	70.98
chr2	205693812	205694009	198	99	0.000	71.33
chr2	210756771	210757120	350	65	0.000	55.99
chr2	212282423	212282554	132	66	0.000	71.16
chr2	212627108	212627268	161	80	0.000	33.6
chr2	214350206	214350375	170	85	0.000	62.12
chr2	215375342	215375561	220	110	0.000	75.88
chr2	216951507	216951642	136	68	0.000	63.12
chr2	222291909	222292068	160	80	0.000	46.67
chr2	223625760	223625914	155	77	0.000	34.57
chr2	225373198	225373378	181	90	0.000	68.45
chr2	226512106	226512237	132	66	0.000	32.56
chr2	229463731	229464076	346	68	0.000	62.53
chr2	230703048	230703306	259	159	0.000	74.64
chr2	232062601	232062817	217	108	0.000	70.66
chr2	234714741	234714867	127	63	0.000	74.47
chr2	239298909	239299191	283	203	0.000	66.48
chr2	241353388	241353534	147	73	0.000	32.46
chr20	485441	485658	218	109	0.000	33.6
chr20	3436619	3436808	190	95	0.000	68.45
chr20	5141147	5141351	205	102	0.000	63.8
chr20	9700803	9700992	190	95	0.000	76.84
chr20	9794711	9794835	125	62	0.000	73.29
chr20	12085282	12085468	187	93	0.000	62.61
chr20	12821792	12822018	227	113	0.000	70.66
chr20	13852463	13852730	268	202	0.000	63.3
chr20	15145149	15145332	184	92	0.000	61.62
chr20	15741537	15741705	169	84	0.000	73.92
chr20	18413033	18413158	126	63	0.000	34.57
chr20	18722264	18722452	189	94	0.000	63.33
chr20	19290136	19290467	332	271	0.000	59.49
chr20	19384899	19385089	191	95	0.000	55.6
chr20	19766304	19766443	140	70	0.000	75.86
chr20	19925702	19926036	335	72	0.000	67.67
chr20	20343243	20343366	124	62	0.000	56.29
chr20	21805720	21805880	161	80	0.000	48.78
chr20	29518705	29518870	166	83	0.000	73.41
chr20	30777539	30777663	125	62	0.000	27.56
chr20	32692634	32692867	234	117	0.000	74.52
chr20	33336956	33337240	285	61	0.000	76.88
chr20	35428349	35428472	124	62	0.000	71.69
chr20	39324993	39325136	144	72	0.000	32.46
chr20	40137049	40137350	302	205	0.000	62.34
chr20	42282393	42282636	244	107	0.000	54.55
chr20	47794191	47794507	317	232	0.000	54.58
chr20	50175420	50175702	283	171	0.000	61.03
chr20	52182615	52182796	182	91	0.000	75.75
chr20	54408931	54409123	193	96	0.000	47.53
chr20	54418057	54418438	382	101	0.000	71.91
chr20	55090569	55090712	144	72	0.000	65.6
chr20	56491063	56491231	169	84	0.000	72.66
chr20	57589668	57589892	225	112	0.000	63.43
chr21	10536029	10536269	241	72	0.000	69.67

chr21	11055580	11055878	299	66	0.000	74.25
chr21	11056229	11056575	347	117	0.000	67.15
chr21	11081877	11082214	338	103	0.000	71.77
chr21	11090172	11090301	130	65	0.000	50.94
chr21	15353054	15353379	326	81	0.000	64.48
chr21	17318432	17318621	190	95	0.000	71.4
chr21	18735526	18735703	178	89	0.000	64.92
chr21	19714939	19715066	128	64	0.000	38.12
chr21	20851190	20851374	185	92	0.000	68.45
chr21	21091918	21092078	161	80	0.000	35.76
chr21	22949230	22949377	148	74	0.000	64.61
chr21	23085764	23085990	227	113	0.000	70.66
chr21	23764630	23764799	170	85	0.000	62.12
chr21	26420673	26420877	205	102	0.000	73.19
chr21	26497647	26497824	178	89	0.000	32.46
chr21	30733829	30733999	171	85	0.000	35.76
chr21	34886829	34887035	207	103	0.000	72.66
chr21	37216220	37216405	186	93	0.000	68.45
chr21	38610588	38610787	200	100	0.000	61.54
chr21	39725789	39726085	297	138	0.000	51.91
chr21	43366881	43367213	333	271	0.000	64.04
chr21	43391352	43391543	192	96	0.000	63.33
chr22	16852174	16852310	137	68	0.000	65.6
chr22	18512839	18512982	144	72	0.000	35.76
chr22	18544271	18544429	159	79	0.000	45.33
chr22	18717894	18718281	388	239	0.000	72.91
chr22	18721145	18721464	320	113	0.000	67.53
chr22	19684222	19684375	154	77	0.000	53.85
chr22	19712807	19712965	159	79	0.000	45.33
chr22	20657751	20658017	267	115	0.000	64.2
chr22	25369146	25369283	138	69	0.000	35.76
chr22	29966401	29966623	223	111	0.000	51.13
chr22	33087155	33087335	181	90	0.000	57.22
chr22	33647668	33647832	165	82	0.000	45.33
chr22	35319027	35319308	282	215	0.000	69.69
chr22	44749269	44749623	355	238	0.000	75.58
chr22	46200488	46200752	265	81	0.000	50.75
chr22	49311470	49311606	137	68	0.000	75.82
chr3	75099	75253	155	77	0.000	54.8
chr3	498329	498571	243	99	0.000	69.84
chr3	837620	837921	302	81	0.000	69.79
chr3	1613664	1613848	185	92	0.000	63.33
chr3	1615982	1616301	320	81	0.000	60.96
chr3	3346383	3346638	256	195	0.000	66.78
chr3	5116896	5117019	124	62	0.000	53.89
chr3	6064883	6065075	193	96	0.000	63.33
chr3	6173211	6173334	124	62	0.000	35.56
chr3	6259663	6259824	162	81	0.000	54.42
chr3	6289186	6289495	310	215	0.000	70.38
chr3	6635381	6635536	156	78	0.000	66.63
chr3	7228647	7228981	335	230	0.000	67.67
chr3	7474462	7474783	322	101	0.000	45.85
chr3	8070392	8070664	273	91	0.000	63.05
chr3	8942879	8943071	193	96	0.000	57.22
chr3	13107803	13107931	129	64	0.000	38.12
chr3	14180473	14180670	198	99	0.000	64.92
chr3	16385138	16385366	229	114	0.000	46.33
chr3	17431709	17431974	266	119	0.000	75.92
chr3	17821277	17821434	158	79	0.000	45.33
chr3	22065253	22065494	242	127	0.000	65.51

chr3	24447913	24448147	235	117	0.000	71.5
chr3	25891035	25891403	369	200	0.000	71.83
chr3	26724458	26724604	147	73	0.000	32.54
chr3	28752137	28752402	266	96	0.000	75.58
chr3	30599921	30600210	290	111	0.000	67.17
chr3	33203329	33203534	206	103	0.000	73.18
chr3	33534370	33534496	127	63	0.000	72.53
chr3	35678388	35678738	351	93	0.000	74.21
chr3	41649346	41649662	317	235	0.000	45.95
chr3	42710878	42711004	127	63	0.000	34.57
chr3	49593496	49593968	473	138	0.000	73.81
chr3	49894549	49894727	179	89	0.000	64.23
chr3	56355183	56355397	215	107	0.000	55.43
chr3	56652705	56652843	139	69	0.000	50.56
chr3	57180350	57180472	123	61	0.000	31.66
chr3	58067492	58067841	350	264	0.000	76.92
chr3	59092118	59092309	192	96	0.000	47.81
chr3	60809395	60809673	279	155	0.000	72.15
chr3	62525634	62525786	153	76	0.000	54.8
chr3	63732948	63733082	135	67	0.000	66.67
chr3	72176505	72176630	126	63	0.000	26.67
chr3	73561332	73561525	194	97	0.000	63.33
chr3	74977321	74977507	187	93	0.000	66.63
chr3	75891030	75891181	152	76	0.000	68.27
chr3	76252019	76252151	133	66	0.000	36.88
chr3	76419539	76419738	200	100	0.000	62.8
chr3	79885069	79885408	340	103	0.000	71.67
chr3	79964701	79964889	189	94	0.000	71.39
chr3	80007077	80007236	160	80	0.000	75.5
chr3	82199625	82199787	163	81	0.000	32.38
chr3	82485941	82486242	302	116	0.000	76.98
chr3	82891747	82892043	297	131	0.000	61.73
chr3	84721766	84722076	311	98	0.000	76.34
chr3	85676244	85676478	235	117	0.000	72.62
chr3	86814897	86815108	212	106	0.000	60.2
chr3	87025788	87026129	342	174	0.000	72.11
chr3	88289544	88289792	249	156	0.000	72.81
chr3	88496873	88497127	255	148	0.000	72.97
chr3	88639521	88639948	428	281	0.000	71.72
chr3	89047294	89047495	202	101	0.000	32.46
chr3	89430841	89430991	151	75	0.000	75.15
chr3	90487245	90487478	234	117	0.000	75.83
chr3	96836707	96836840	134	67	0.000	36.88
chr3	98649601	98649805	205	102	0.000	70.66
chr3	98697780	98697914	135	67	0.000	75.72
chr3	101090349	101090483	135	67	0.000	75.72
chr3	101622957	101623286	330	109	0.000	68.2
chr3	102581035	102581275	241	115	0.000	71.72
chr3	102985719	102985899	181	90	0.000	76.09
chr3	108209542	108209780	239	164	0.000	75.58
chr3	110341417	110341748	332	269	0.000	68
chr3	111048758	111048888	131	65	0.000	27.56
chr3	112317125	112317341	217	108	0.000	72.62
chr3	112434281	112434545	265	64	0.000	74.29
chr3	113184927	113185324	398	250	0.000	53.81
chr3	113761779	113762121	343	271	0.000	76.73
chr3	116112920	116113222	303	96	0.000	47.63
chr3	119833690	119833879	190	95	0.000	56.19
chr3	124070475	124070815	341	110	0.000	63.05
chr3	124757137	124757475	339	112	0.000	67.17

chr3	125025975	125026217	243	145	0.000	67.17
chr3	125264269	125264396	128	64	0.000	73.48
chr3	127696958	127697210	253	63	0.000	70.82
chr3	129107200	129107403	204	102	0.000	75
chr3	136175201	136175366	166	83	0.000	67.07
chr3	137751512	137751664	153	76	0.000	46.67
chr3	138012839	138013000	162	81	0.000	71.98
chr3	138222050	138222240	191	95	0.000	74.52
chr3	138438482	138438654	173	86	0.000	60.44
chr3	140901840	140902032	193	96	0.000	64.33
chr3	143452078	143452242	165	82	0.000	73.7
chr3	146869102	146869249	148	74	0.000	70.98
chr3	147580302	147580560	259	69	0.000	71.92
chr3	148444658	148445059	402	106	0.000	53.81
chr3	149959300	149959430	131	65	0.000	26.67
chr3	152544252	152544588	337	60	0.000	63.5
chr3	152697155	152697321	167	83	0.000	72.08
chr3	157033611	157033839	229	114	0.000	70.66
chr3	157824314	157824470	157	78	0.000	66.79
chr3	160631193	160631327	135	67	0.000	75.72
chr3	162730733	162730955	223	111	0.000	74.47
chr3	164979948	164980245	298	61	0.000	64.02
chr3	165273431	165273675	245	112	0.000	62.58
chr3	166180992	166181251	260	151	0.000	71.46
chr3	166967995	166968165	171	85	0.000	70.91
chr3	167897247	167897389	143	71	0.000	66.29
chr3	168299002	168299183	182	91	0.000	64.76
chr3	169897796	169898035	240	151	0.000	67.17
chr3	170573924	170574135	212	106	0.000	72.62
chr3	171681402	171681610	209	104	0.000	57.22
chr3	172220087	172220220	134	67	0.000	32.46
chr3	173470050	173470171	122	61	0.000	76.39
chr3	173905076	173905198	123	61	0.000	26.67
chr3	174280565	174280778	214	107	0.000	76.86
chr3	175928028	175928359	332	252	0.000	63.34
chr3	176887843	176887975	133	66	0.000	51.13
chr3	176955233	176955376	144	72	0.000	38.51
chr3	178339054	178339283	230	115	0.000	74.31
chr3	180571626	180571976	351	82	0.000	61.67
chr3	181638081	181638211	131	65	0.000	73.87
chr3	184582796	184583083	288	225	0.000	76.94
chr3	186055817	186055938	122	61	0.000	34.57
chr3	187477985	187478327	343	272	0.000	50.58
chr3	188251538	188251675	138	69	0.000	54.42
chr3	188367721	188367865	145	72	0.000	75.58
chr3	190857465	190857812	348	75	0.000	67.57
chr3	192164017	192164193	177	88	0.000	76.02
chr3	194417929	194418253	325	256	0.000	68.45
chr3	195256368	195256492	125	62	0.000	71.87
chr3	195436576	195436766	191	95	0.000	75.15
chr3	196607829	196607993	165	82	0.000	33.6
chr4	3192541	3192669	129	64	0.000	34.57
chr4	8227675	8227896	222	111	0.000	75.45
chr4	12343498	12343704	207	103	0.000	66.89
chr4	15376901	15377099	199	99	0.000	71.43
chr4	16604871	16605046	176	88	0.000	73.06
chr4	17452938	17453112	175	87	0.000	63.33
chr4	23360727	23360984	258	197	0.000	61.23
chr4	23927506	23927651	146	73	0.000	73.7
chr4	26235509	26235661	153	76	0.000	34.57

chr4	27466444	27466574	131	65	0.000	26.67
chr4	30476082	30476250	169	84	0.000	35.76
chr4	31863471	31863616	146	73	0.000	45.8
chr4	34625615	34625944	330	268	0.000	63.76
chr4	34801047	34801178	132	66	0.000	46.67
chr4	35251546	35251699	154	77	0.000	66.51
chr4	36605107	36605375	269	191	0.000	63.84
chr4	37095500	37095647	148	74	0.000	70.91
chr4	37759536	37759746	211	105	0.000	71.69
chr4	39895946	39896279	334	239	0.000	63.83
chr4	43326746	43326966	221	110	0.000	76.53
chr4	43355992	43356155	164	82	0.000	32.46
chr4	45642271	45642496	226	113	0.000	68.48
chr4	47911332	47911533	202	101	0.000	75
chr4	48467599	48467869	271	203	0.000	71.51
chr4	48615208	48615375	168	84	0.000	73.93
chr4	49291490	49292027	538	240	0.000	75.93
chr4	53857459	53857641	183	91	0.000	76.91
chr4	55407377	55407579	203	101	0.000	72.62
chr4	56337022	56337258	237	145	0.000	66.75
chr4	57137293	57137587	295	160	0.000	60.39
chr4	57319665	57319789	125	62	0.000	71.87
chr4	61676023	61676253	231	115	0.000	72.62
chr4	61756420	61756628	209	104	0.000	31.96
chr4	65033245	65033472	228	114	0.000	72.62
chr4	66187275	66187477	203	101	0.000	70.66
chr4	66440619	66440772	154	77	0.000	53.85
chr4	66565863	66566210	348	179	0.000	64.39
chr4	67842679	67842858	180	90	0.000	76.37
chr4	71585596	71585800	205	102	0.000	67.5
chr4	72566947	72567282	336	274	0.000	60.51
chr4	72894786	72894927	142	71	0.000	69.72
chr4	75385053	75385259	207	103	0.000	75
chr4	79167615	79167764	150	75	0.000	45.33
chr4	81248842	81249081	240	100	0.000	70.04
chr4	81653215	81653368	154	77	0.000	57.22
chr4	81727892	81728179	288	182	0.000	74.7
chr4	83353346	83353583	238	123	0.000	34.57
chr4	84162260	84162529	270	202	0.000	71.53
chr4	85012793	85012983	191	95	0.000	64.33
chr4	89024282	89024480	199	99	0.000	75
chr4	91497760	91498097	338	248	0.000	72.79
chr4	91779655	91779971	317	92	0.000	61.02
chr4	93026696	93026828	133	66	0.000	32.46
chr4	95092215	95092388	174	87	0.000	68.45
chr4	96594467	96594666	200	100	0.000	65.6
chr4	97618497	97618683	187	93	0.000	72.85
chr4	99116018	99116173	156	78	0.000	33.6
chr4	99371255	99371458	204	102	0.000	72.44
chr4	101809404	101809549	146	73	0.000	0
chr4	102162153	102162284	132	66	0.000	40
chr4	102639730	102639996	267	149	0.000	72.38
chr4	104864338	104864613	276	116	0.000	71
chr4	105043753	105043971	219	109	0.000	72.23
chr4	105471066	105471374	309	62	0.000	70.43
chr4	106627178	106627304	127	63	0.000	73.29
chr4	106630590	106630725	136	68	0.000	54.42
chr4	108376564	108376724	161	80	0.000	73.29
chr4	112523986	112524306	321	107	0.000	68.68
chr4	116323953	116324219	267	207	0.000	70.62

chr4	117181028	117181152	125	62	0.000	71.87
chr4	119637350	119637903	554	220	0.000	64.27
chr4	124687973	124688126	154	77	0.000	66.51
chr4	125073697	125073822	126	63	0.000	26.67
chr4	126958053	126958263	211	105	0.000	75
chr4	127863606	127863863	258	96	0.000	75.58
chr4	128297169	128297398	230	115	0.000	74.31
chr4	128356020	128356221	202	101	0.000	72.62
chr4	130413664	130413813	150	75	0.000	33.6
chr4	131172227	131172593	367	249	0.000	35.53
chr4	131531062	131531193	132	66	0.000	34.57
chr4	133958105	133958259	155	77	0.000	54.02
chr4	134453788	134453933	146	73	0.000	32.46
chr4	134576411	134576663	253	181	0.000	70.82
chr4	137267102	137267297	196	98	0.000	32.84
chr4	137878632	137878944	313	81	0.000	63.96
chr4	139412238	139412431	194	97	0.000	32.56
chr4	142089878	142090048	171	85	0.000	35.76
chr4	145629166	145629504	339	224	0.000	67.17
chr4	147609159	147609419	261	116	0.000	65.08
chr4	150339044	150339270	227	113	0.000	33.6
chr4	150793111	150793260	150	75	0.000	74.18
chr4	152440280	152440446	167	83	0.000	73.86
chr4	152912067	152912274	208	104	0.000	63.33
chr4	152986352	152986486	135	67	0.000	72.2
chr4	153219089	153219238	150	75	0.000	33.6
chr4	153602105	153602292	188	94	0.000	68.45
chr4	156908287	156908514	228	114	0.000	75
chr4	157419627	157420045	419	302	0.000	32.29
chr4	158103615	158103739	125	62	0.000	73.81
chr4	159530195	159530586	392	275	0.000	71.93
chr4	162078187	162078376	190	95	0.000	64.92
chr4	163395674	163395817	144	72	0.000	72.86
chr4	166104145	166104496	352	115	0.000	61.48
chr4	167047523	167047825	303	136	0.000	76.93
chr4	167699967	167700090	124	62	0.000	61.62
chr4	169487304	169487511	208	104	0.000	60.74
chr4	172045878	172046044	167	83	0.000	33.6
chr4	176785119	176785281	163	81	0.000	72.5
chr4	179960625	179960765	141	70	0.000	45.8
chr4	180281614	180281853	240	103	0.000	67.17
chr4	181972085	181972261	177	88	0.000	74.52
chr4	182201965	182202143	179	89	0.000	76.1
chr4	182495804	182495933	130	65	0.000	26.67
chr4	184416782	184417018	237	119	0.000	72.15
chr4	186040024	186040339	316	89	0.000	61.84
chr4	186046877	186047004	128	64	0.000	62.5
chr4	189068686	189069030	345	93	0.000	63.53
chr4	189594245	189594371	127	63	0.000	74.47
chr5	7209531	7209711	181	90	0.000	69.64
chr5	7564443	7564613	171	85	0.000	67.16
chr5	7678458	7678719	262	147	0.000	64.1
chr5	10892194	10892336	143	71	0.000	45.8
chr5	12965122	12965340	219	109	0.000	63.33
chr5	16157494	16157856	363	82	0.000	47.45
chr5	16607675	16607797	123	61	0.000	26.67
chr5	19964311	19964442	132	66	0.000	74.45
chr5	21611902	21612033	132	66	0.000	34.57
chr5	22873655	22873881	227	113	0.000	75
chr5	24067606	24067762	157	78	0.000	57.22

chr5	24476681	24476832	152	76	0.000	45.33
chr5	24964352	24964641	290	185	0.000	72.92
chr5	26460070	26460197	128	64	0.000	52.17
chr5	28721225	28721418	194	97	0.000	75.24
chr5	30669960	30670293	334	62	0.000	67.87
chr5	31014105	31014380	276	193	0.000	69.89
chr5	33631100	33631284	185	92	0.000	53.89
chr5	33954228	33954362	135	67	0.000	54.65
chr5	33988773	33988935	163	81	0.000	72.85
chr5	34028028	34028221	194	97	0.000	64.84
chr5	34588873	34589156	284	63	0.000	61.07
chr5	35816251	35816434	184	92	0.000	76.18
chr5	37003552	37003761	210	105	0.000	72.62
chr5	38143069	38143364	296	183	0.000	75.98
chr5	39604427	39604716	290	66	0.000	73.82
chr5	40236893	40237099	207	103	0.000	70.66
chr5	41674251	41674606	356	106	0.000	45.42
chr5	41927792	41927924	133	66	0.000	66.67
chr5	43750428	43750592	165	82	0.000	35.76
chr5	44634340	44634645	306	72	0.000	72.06
chr5	44638915	44639211	297	200	0.000	71.55
chr5	45100785	45101136	352	102	0.000	64.08
chr5	45857372	45857591	220	110	0.000	75.42
chr5	49547362	49547697	336	230	0.000	71.66
chr5	49775442	49775717	276	114	0.000	73.61
chr5	53025151	53025281	131	65	0.000	53.89
chr5	59589927	59590366	440	127	0.000	71.42
chr5	59675954	59676122	169	84	0.000	57.22
chr5	61270809	61271160	352	269	0.000	64.08
chr5	62251758	62252046	289	167	0.000	71.36
chr5	66149963	66150088	126	63	0.000	34.57
chr5	67106783	67106922	140	70	0.000	36.88
chr5	67160991	67161318	328	120	0.000	60.83
chr5	72090918	72091123	206	103	0.000	66.92
chr5	72094585	72094770	186	93	0.000	54.8
chr5	74520332	74520553	222	111	0.000	73.8
chr5	75118649	75118785	137	68	0.000	45.8
chr5	78914690	78915025	336	127	0.000	70.28
chr5	83076451	83076608	158	79	0.000	74.5
chr5	84254403	84254823	421	309	0.000	67.94
chr5	84900014	84900173	160	80	0.000	71.56
chr5	85092546	85092887	342	277	0.000	67.34
chr5	86839077	86839247	171	85	0.000	63.33
chr5	89008550	89008873	324	254	0.000	66.55
chr5	90744885	90745033	149	74	0.000	57.22
chr5	93225232	93225513	282	104	0.000	66.63
chr5	95062084	95062400	317	60	0.000	75.41
chr5	97611890	97612152	263	201	0.000	70.77
chr5	97751097	97751286	190	95	0.000	75.28
chr5	97843687	97843857	171	85	0.000	68.45
chr5	98356137	98356450	314	62	0.000	69.85
chr5	99759926	99760238	313	92	0.000	60.88
chr5	100722725	100722997	273	189	0.000	71.44
chr5	102494058	102494379	322	253	0.000	75.58
chr5	104881946	104882193	248	141	0.000	66.58
chr5	105589165	105589424	260	98	0.000	75.39
chr5	108292991	108293250	260	112	0.000	64.2
chr5	112952229	112952534	306	112	0.000	68.44
chr5	113580770	113581174	405	305	0.000	71.5
chr5	115712819	115713108	290	77	0.000	67.17

chr5	117553388	117553555	168	84	0.000	50.58
chr5	118988557	118988903	347	238	0.000	66.54
chr5	121036842	121037028	187	93	0.000	74.52
chr5	123879647	123879820	174	87	0.000	74.59
chr5	124621551	124621800	250	95	0.000	66.26
chr5	124767562	124767686	125	62	0.000	52.17
chr5	124910875	124911002	128	64	0.000	34.57
chr5	127611994	127612199	206	103	0.000	72.81
chr5	129867788	129868086	299	64	0.000	63
chr5	130266786	130267007	222	111	0.000	68.24
chr5	131918826	131919034	209	104	0.000	70.66
chr5	133479891	133480021	131	65	0.000	51.13
chr5	135035061	135035192	132	66	0.000	74.45
chr5	136677909	136678103	195	97	0.000	71.32
chr5	138218084	138218212	129	64	0.000	73.5
chr5	138336442	138336616	175	87	0.000	70.91
chr5	141998006	141998356	351	113	0.000	75.39
chr5	144170326	144170531	206	103	0.000	64.59
chr5	144456944	144457101	158	79	0.000	64.78
chr5	144888514	144888714	201	100	0.000	75
chr5	145192671	145192796	126	63	0.000	32.68
chr5	146496420	146496618	199	99	0.000	45.8
chr5	146702649	146703118	470	195	0.000	70.38
chr5	149674205	149674373	169	84	0.000	32.46
chr5	150958102	150958433	332	79	0.000	72.87
chr5	151975377	151975545	169	84	0.000	76.52
chr5	152741883	152742178	296	117	0.000	75.98
chr5	153472066	153472333	268	113	0.000	61.11
chr5	154135897	154136028	132	66	0.000	45.45
chr5	154967547	154967754	208	104	0.000	72.62
chr5	161051780	161051989	210	105	0.000	63.27
chr5	161869072	161869403	332	67	0.000	64.12
chr5	162591940	162592122	183	91	0.000	70.04
chr5	163404939	163405106	168	84	0.000	57.22
chr5	164544164	164544294	131	65	0.000	56.29
chr5	166799762	166800136	375	189	0.000	64.66
chr5	167219457	167219608	152	76	0.000	71.69
chr5	167675632	167675782	151	75	0.000	56.29
chr5	173483177	173483321	145	72	0.000	67.21
chr5	174140176	174140303	128	64	0.000	34.57
chr5	180529233	180529417	185	92	0.000	66.33
chr5	180634748	180635100	353	98	0.000	61.99
chr6	2714454	2714574	121	60	0.000	56.29
chr6	2762322	2762459	138	69	0.000	35.76
chr6	3488613	3488812	200	100	0.000	73.49
chr6	5212511	5212688	178	89	0.000	68.45
chr6	8531843	8532268	426	282	0.000	71.73
chr6	9087340	9087583	244	148	0.000	71.71
chr6	9818554	9818715	162	81	0.000	45.8
chr6	12568183	12568428	246	184	0.000	70.34
chr6	14092745	14092908	164	82	0.000	46.33
chr6	17882333	17882568	236	123	0.000	62.93
chr6	18650296	18650427	132	66	0.000	74.45
chr6	21129080	21129323	244	126	0.000	73.92
chr6	21909534	21909705	172	86	0.000	62.73
chr6	22072745	22072873	129	64	0.000	45.18
chr6	23568981	23569435	455	236	0.000	71.76
chr6	24127560	24127721	162	81	0.000	57.22
chr6	25988051	25988284	234	117	0.000	70.66
chr6	26577162	26577538	377	262	0.000	71.55

chr6	27271550	27271708	159	79	0.000	38.06
chr6	28863996	28864185	190	95	0.000	76.84
chr6	28949942	28950103	162	81	0.000	61.62
chr6	30180620	30180798	179	89	0.000	71.71
chr6	33283358	33283487	130	65	0.000	34.57
chr6	33762877	33763161	285	224	0.000	71.54
chr6	35052321	35052453	133	66	0.000	32.46
chr6	35594931	35595062	132	66	0.000	74.45
chr6	39491730	39491979	250	95	0.000	62.87
chr6	40184528	40184679	152	76	0.000	45.33
chr6	41365159	41365384	226	113	0.000	76.27
chr6	49098200	49098405	206	103	0.000	68.45
chr6	49415113	49415251	139	69	0.000	63.74
chr6	50348632	50348779	148	74	0.000	65.6
chr6	54842730	54842901	172	86	0.000	60.44
chr6	55524040	55524246	207	103	0.000	66.89
chr6	56416405	56416526	122	61	0.000	71.82
chr6	57395910	57396208	299	214	0.000	66.2
chr6	58156427	58156626	200	100	0.000	73.49
chr6	62777820	62777959	140	70	0.000	67.08
chr6	64072159	64072338	180	90	0.000	68.45
chr6	64274772	64275119	348	247	0.000	66.64
chr6	64875967	64876256	290	106	0.000	75.83
chr6	68721536	68721766	231	115	0.000	75.98
chr6	69689663	69689848	186	93	0.000	64.92
chr6	74260570	74260738	169	84	0.000	45.33
chr6	74529732	74530033	302	199	0.000	76.25
chr6	76117260	76117607	348	94	0.000	54.55
chr6	78415217	78415547	331	107	0.000	63.62
chr6	79614613	79614754	142	71	0.000	35.76
chr6	81895439	81895601	163	81	0.000	43.59
chr6	86051409	86051572	164	82	0.000	45.33
chr6	88933581	88933860	280	177	0.000	68.93
chr6	89263269	89263462	194	97	0.000	34.57
chr6	92649827	92650015	189	94	0.000	76.29
chr6	93218544	93218763	220	110	0.000	75.89
chr6	93566090	93566297	208	104	0.000	70.66
chr6	95175173	95175405	233	116	0.000	75
chr6	95345110	95345237	128	64	0.000	26.67
chr6	96112266	96112399	134	67	0.000	65.6
chr6	96518010	96518345	336	161	0.000	75.74
chr6	97853936	97854210	275	85	0.000	69.95
chr6	97912042	97912372	331	115	0.000	53.81
chr6	99682903	99683232	330	144	0.000	63.76
chr6	101303724	101303851	128	64	0.000	73.48
chr6	103753523	103753775	253	144	0.000	71.93
chr6	106677212	106677418	207	103	0.000	66.89
chr6	109424173	109424461	289	77	0.000	71.28
chr6	109681855	109682156	302	239	0.000	62.34
chr6	110542414	110542655	242	110	0.000	74.14
chr6	113047612	113047763	152	76	0.000	57.22
chr6	114323732	114324094	363	102	0.000	63.64
chr6	114412943	114413152	210	105	0.000	66.17
chr6	115266879	115267085	207	103	0.000	75
chr6	115813002	115813185	184	92	0.000	71.45
chr6	116108611	116108748	138	69	0.000	63.42
chr6	118282452	118282685	234	117	0.000	75
chr6	119098311	119098485	175	87	0.000	67.64
chr6	120459547	120459782	236	173	0.000	64.92
chr6	122048742	122048871	130	65	0.000	73.79

chr6	127252747	127252887	141	70	0.000	32.46
chr6	127855077	127855369	293	210	0.000	60.62
chr6	135590559	135590682	124	62	0.000	26.67
chr6	140979409	140979629	221	110	0.000	63.18
chr6	142144624	142144885	262	73	0.000	72.1
chr6	142965873	142966179	307	152	0.000	75.58
chr6	146962024	146962158	135	67	0.000	35.76
chr6	152322965	152323129	165	82	0.000	54.42
chr6	154801060	154801255	196	98	0.000	75.83
chr6	155634019	155634206	188	94	0.000	63.33
chr6	160471079	160471398	320	73	0.000	69.01
chr6	163162344	163162493	150	75	0.000	74.96
chr7	645687	645911	225	112	0.000	73.88
chr7	959213	959384	172	86	0.000	76.81
chr7	1751136	1751301	166	83	0.000	54.42
chr7	2449527	2449708	182	91	0.000	63.91
chr7	3040537	3040927	391	102	0.000	66.83
chr7	4437637	4437798	162	81	0.000	73.07
chr7	5903533	5903658	126	63	0.000	31.82
chr7	9553010	9553312	303	207	0.000	75.58
chr7	9895074	9895258	185	92	0.000	63.84
chr7	12970153	12970521	369	99	0.000	53.81
chr7	13704860	13705025	166	83	0.000	47.53
chr7	14276551	14276878	328	247	0.000	68.31
chr7	14616667	14616904	238	102	0.000	45.33
chr7	14665923	14666148	226	113	0.000	64.92
chr7	15020905	15021153	249	62	0.000	64.54
chr7	17934521	17934799	279	201	0.000	69.14
chr7	17938177	17938406	230	115	0.000	67.84
chr7	18440255	18440400	146	73	0.000	45.8
chr7	18511487	18511726	240	157	0.000	70.04
chr7	26618270	26618459	190	95	0.000	76.84
chr7	28679058	28679335	278	109	0.000	75.58
chr7	29402376	29402732	357	296	0.000	66.67
chr7	30169724	30170043	320	144	0.000	62.67
chr7	35251889	35252090	202	101	0.000	75
chr7	37466540	37466740	201	100	0.000	71.91
chr7	39899581	39899999	419	211	0.000	75.58
chr7	41989062	41989234	173	86	0.000	60.44
chr7	42339043	42339374	332	66	0.000	63.34
chr7	42490283	42490470	188	94	0.000	70.91
chr7	43701356	43701521	166	83	0.000	73.41
chr7	46255520	46255971	452	109	0.000	62.07
chr7	46296642	46296833	192	96	0.000	60.44
chr7	46974724	46974916	193	96	0.000	71.48
chr7	48413398	48413589	192	96	0.000	74.52
chr7	49871172	49871329	158	79	0.000	75.15
chr7	50330599	50330911	313	251	0.000	66.7
chr7	52507409	52507645	237	177	0.000	75
chr7	65926031	65926257	227	113	0.000	70.66
chr7	68031303	68031489	187	93	0.000	71.06
chr7	71363718	71363949	232	116	0.000	63.9
chr7	73337684	73337967	284	220	0.000	66.6
chr7	75192995	75193345	351	258	0.000	47.13
chr7	76999133	76999276	144	72	0.000	34.57
chr7	77127518	77127680	163	81	0.000	54.42
chr7	79763388	79763720	333	137	0.000	71.75
chr7	80077376	80077692	317	216	0.000	61.71
chr7	81048994	81049412	419	169	0.000	71.82
chr7	90007143	90007264	122	61	0.000	50

chr7	90787780	90787964	185	92	0.000	60.44
chr7	91190087	91190276	190	95	0.000	68.45
chr7	95776232	95776525	294	177	0.000	71.32
chr7	96304906	96305255	350	78	0.000	61.86
chr7	100182689	100182989	301	219	0.000	68.14
chr7	101887386	101887551	166	83	0.000	71.86
chr7	102537344	102537623	280	171	0.000	69.61
chr7	104308294	104308525	232	116	0.000	66.45
chr7	105746119	105746320	202	101	0.000	76.81
chr7	106073965	106074270	306	196	0.000	62.09
chr7	106268662	106268806	145	72	0.000	53.72
chr7	107717517	107717914	398	125	0.000	73.91
chr7	109586969	109587398	430	202	0.000	71.93
chr7	112128495	112128641	147	73	0.000	32.51
chr7	112273504	112273865	362	197	0.000	64.96
chr7	114198750	114199043	294	131	0.000	75.97
chr7	115221775	115221954	180	90	0.000	63.33
chr7	115635964	115636114	151	75	0.000	76.63
chr7	116041563	116041720	158	79	0.000	57.22
chr7	119116276	119116464	189	94	0.000	76.29
chr7	122268482	122268630	149	74	0.000	33.6
chr7	123345436	123345733	298	203	0.000	53.81
chr7	124312984	124313152	169	84	0.000	32.46
chr7	125882954	125883082	129	64	0.000	73.5
chr7	126057308	126057447	140	70	0.000	75.86
chr7	126515169	126515504	336	147	0.000	63.77
chr7	126742934	126743107	174	87	0.000	38.06
chr7	128208033	128208290	258	160	0.000	72.01
chr7	132885086	132885259	174	87	0.000	63.43
chr7	133852597	133852827	231	115	0.000	73.7
chr7	133917363	133917504	142	71	0.000	69.72
chr7	136752698	136753030	333	85	0.000	67.97
chr7	137032378	137032505	128	64	0.000	73.48
chr7	144404291	144404432	142	71	0.000	38.06
chr7	145312819	145313004	186	93	0.000	45.72
chr7	147534967	147535109	143	71	0.000	34.57
chr7	148134677	148134807	131	65	0.000	34.57
chr7	149766565	149766698	134	67	0.000	71.58
chr7	153879651	153879785	135	67	0.000	35.76
chr7	154592364	154592495	132	66	0.000	61.01
chr7	155838149	155838486	338	91	0.000	67.54
chr7	156300125	156300322	198	99	0.000	63.91
chr7	157411808	157411955	148	74	0.000	70.98
chr7	157954984	157955111	128	64	0.000	52
chr8	2948740	2949028	289	181	0.000	63.55
chr8	3883851	3884036	186	93	0.000	74.52
chr8	5985829	5986124	296	189	0.000	63.07
chr8	8432602	8432866	265	155	0.000	74.29
chr8	9208546	9208738	193	96	0.000	63.33
chr8	9761653	9761797	145	72	0.000	75.58
chr8	10110200	10110499	300	77	0.000	65.13
chr8	16040612	16040897	286	225	0.000	60.08
chr8	17169107	17169286	180	90	0.000	72.66
chr8	18171607	18171980	374	274	0.000	73.3
chr8	18661896	18662030	135	67	0.000	45.8
chr8	21520996	21521273	278	108	0.000	75.58
chr8	24669978	24670258	281	95	0.000	66.02
chr8	26423891	26424116	226	113	0.000	62.33
chr8	27213429	27213760	332	233	0.000	75.33
chr8	27366237	27366360	124	62	0.000	42.42

chr8	33814653	33815127	475	363	0.000	67.23
chr8	35788490	35788643	154	77	0.000	69.94
chr8	35809878	35810248	371	113	0.000	67.55
chr8	36255816	36256022	207	103	0.000	61.44
chr8	39568350	39568561	212	106	0.000	74.8
chr8	40511966	40512356	391	215	0.000	75.58
chr8	41745596	41745778	183	91	0.000	45.33
chr8	42606490	42606623	134	67	0.000	45.36
chr8	43092704	43093992	1289	232	0.000	63.16
chr8	43691154	43691477	324	87	0.000	70.44
chr8	47177610	47177776	167	83	0.000	57.22
chr8	52017566	52017740	175	87	0.000	75.14
chr8	52786792	52786934	143	71	0.000	76.79
chr8	53763661	53763790	130	65	0.000	26.67
chr8	55142996	55143120	125	62	0.000	34.57
chr8	59522764	59522887	124	62	0.000	61.11
chr8	59964094	59964224	131	65	0.000	27.56
chr8	60294239	60294423	185	92	0.000	74.52
chr8	61405985	61406260	276	111	0.000	64.2
chr8	63707512	63707872	361	65	0.000	74.04
chr8	65875456	65875631	176	88	0.000	47.8
chr8	69194601	69194758	158	79	0.000	47.53
chr8	69629549	69629672	124	62	0.000	34.57
chr8	69876855	69877171	317	112	0.000	74.36
chr8	71565557	71565867	311	206	0.000	62.64
chr8	73669376	73669507	132	66	0.000	32.85
chr8	75628138	75628488	351	60	0.000	74.1
chr8	75985114	75985330	217	108	0.000	74.33
chr8	76180046	76180232	187	93	0.000	45.33
chr8	79758041	79758198	158	79	0.000	61.41
chr8	80788908	80789126	219	109	0.000	72.23
chr8	82841700	82841856	157	78	0.000	57.22
chr8	83324479	83324637	159	79	0.000	71.61
chr8	83595239	83595552	314	212	0.000	71.33
chr8	86046804	86047034	231	115	0.000	66.39
chr8	86324975	86325227	253	164	0.000	45.57
chr8	86796592	86797121	530	439	0.000	76.58
chr8	87055116	87055258	143	71	0.000	46.75
chr8	87903852	87904027	176	88	0.000	63.48
chr8	88228557	88228795	239	111	0.000	70.09
chr8	90311674	90311868	195	97	0.000	60.44
chr8	92981118	92981364	247	184	0.000	60.54
chr8	93970394	93970732	339	98	0.000	60.19
chr8	94047253	94047390	138	69	0.000	45.8
chr8	95311044	95311294	251	83	0.000	73.39
chr8	96273924	96274253	330	76	0.000	75.08
chr8	96342169	96342497	329	236	0.000	75.3
chr8	97920294	97920584	291	111	0.000	64.67
chr8	100446838	100447021	184	92	0.000	75.83
chr8	100579126	100579315	190	95	0.000	64.92
chr8	101691923	101692108	186	93	0.000	26.45
chr8	102193567	102193914	348	211	0.000	75.94
chr8	103329534	103329739	206	103	0.000	70.66
chr8	106879038	106879221	184	92	0.000	74.52
chr8	107101620	107101795	176	88	0.000	74.52
chr8	107248216	107248380	165	82	0.000	45.33
chr8	108860460	108860633	174	87	0.000	63.33
chr8	109040949	109041254	306	109	0.000	71.37
chr8	109371979	109372141	163	81	0.000	72.5
chr8	117291540	117291877	338	226	0.000	76.47

chr8	117498195	117498335	141	70	0.000	76.21
chr8	119365835	119366256	422	166	0.000	66.02
chr8	124112033	124112309	277	124	0.000	73.67
chr8	124402243	124402416	174	87	0.000	63.33
chr8	125300084	125300360	277	167	0.000	63.91
chr8	125862350	125862508	159	79	0.000	69.39
chr8	125980169	125980384	216	108	0.000	72.62
chr8	126666649	126666969	321	209	0.000	72.39
chr8	129362612	129363090	479	224	0.000	63.99
chr8	130671012	130671364	353	75	0.000	66.23
chr8	132351880	132352062	183	91	0.000	76.91
chr8	134831860	134832192	333	60	0.000	64.04
chr8	135942531	135942817	287	198	0.000	76.78
chr8	136800756	136801060	305	244	0.000	70.67
chr8	138684886	138685232	347	281	0.000	68.1
chr8	139598704	139598826	123	61	0.000	71.59
chr8	141434052	141434307	256	191	0.000	61.42
chr8	142911721	142911944	224	112	0.000	70.66
chr9	518859	518992	134	67	0.000	75.47
chr9	1945618	1946082	465	333	0.000	73.96
chr9	3009450	3009795	346	88	0.000	66.73
chr9	5020675	5020798	124	62	0.000	34.57
chr9	7789047	7789221	175	87	0.000	32.46
chr9	16480819	16480941	123	61	0.000	32.56
chr9	17738833	17739050	218	109	0.000	67.84
chr9	18044590	18044785	196	98	0.000	71.01
chr9	22644567	22644763	197	98	0.000	71.07
chr9	23860432	23860626	195	97	0.000	64.9
chr9	28229817	28230042	226	113	0.000	62.33
chr9	31742495	31742719	225	112	0.000	46.11
chr9	32801426	32801619	194	97	0.000	64.92
chr9	36290493	36290828	336	256	0.000	62.46
chr9	36636434	36636558	125	62	0.000	26.67
chr9	44541809	44541981	173	86	0.000	71.68
chr9	46057549	46057822	274	108	0.000	64.53
chr9	66840645	66840809	165	82	0.000	66.64
chr9	66844827	66845285	459	206	0.000	71.72
chr9	66970955	66971574	620	287	0.000	62.72
chr9	69710606	69711580	975	507	0.000	74.47
chr9	70006421	70006650	230	115	0.000	74.31
chr9	72707691	72707901	211	105	0.000	66.63
chr9	74160284	74160458	175	87	0.000	64.92
chr9	79366772	79367121	350	117	0.000	73.91
chr9	82622003	82622297	295	117	0.000	70.81
chr9	83848327	83848501	175	87	0.000	74.9
chr9	84753831	84754015	185	92	0.000	63.33
chr9	87026560	87026703	144	72	0.000	76.7
chr9	88150716	88150840	125	62	0.000	50
chr9	91323918	91324182	265	147	0.000	72.29
chr9	92681581	92681737	157	78	0.000	33.6
chr9	93471069	93471340	272	66	0.000	73.22
chr9	93905216	93905339	124	62	0.000	32.56
chr9	100999778	101000126	349	70	0.000	66.57
chr9	106292948	106293200	253	60	0.000	70.82
chr9	106645452	106645645	194	97	0.000	68.45
chr9	109043937	109044276	340	65	0.000	67.17
chr9	109320918	109321082	165	82	0.000	35.56
chr9	109600412	109600560	149	74	0.000	55.79
chr9	111592980	111593181	202	101	0.000	74.12
chr9	112357191	112357527	337	83	0.000	67.5

chr9	114385307	114385451	145	72	0.000	54.42
chr9	114717223	114717442	220	110	0.000	75
chr9	114916919	114917086	168	84	0.000	75.15
chr9	118041164	118041403	240	66	0.000	74.81
chr9	118998420	118998551	132	66	0.000	38.12
chr9	119347382	119347654	273	200	0.000	69.78
chr9	120822259	120822478	220	110	0.000	75
chr9	121460613	121460813	201	100	0.000	74.92
chr9	121472010	121472337	328	214	0.000	63.78
chr9	123652617	123652777	161	80	0.000	45.33
chr9	125816490	125816704	215	107	0.000	75.86
chr9	126210299	126210471	173	86	0.000	64.92
chr9	131507235	131507365	131	65	0.000	46.67
chr9	132119176	132119412	237	98	0.000	63.92
chr9	132614765	132615109	345	81	0.000	73.81
chr9	136190975	136191177	203	101	0.000	75
chrX	4232795	4232941	147	73	0.000	70.91
chrX	6388930	6389085	156	78	0.000	57.22
chrX	9258727	9258911	185	92	0.000	34.57
chrX	10443290	10443440	151	75	0.000	26.67
chrX	12508305	12508510	206	103	0.000	64.92
chrX	12653260	12653574	315	171	0.000	64.96
chrX	15295671	15295890	220	110	0.000	75
chrX	16753411	16753598	188	94	0.000	76.48
chrX	19696220	19696384	165	82	0.000	33.6
chrX	21187856	21188102	247	115	0.000	72.56
chrX	27260601	27260792	192	96	0.000	76.84
chrX	28892392	28892603	212	106	0.000	72.22
chrX	32185146	32185333	188	94	0.000	63.33
chrX	32895049	32895238	190	95	0.000	73.23
chrX	34706218	34706433	216	108	0.000	70.66
chrX	36706943	36707139	197	98	0.000	65.25
chrX	38023762	38024147	386	133	0.000	71.05
chrX	40510387	40510572	186	93	0.000	68.45
chrX	43592329	43592466	138	69	0.000	66.67
chrX	44823975	44824152	178	89	0.000	64.92
chrX	55172385	55172684	300	223	0.000	65.13
chrX	57912721	57912862	142	71	0.000	76.68
chrX	58578425	58580300	1876	486	0.000	73.59
chrX	61837521	61837666	146	73	0.000	65.6
chrX	66970654	66970907	254	75	0.000	66.83
chrX	69115160	69115407	248	145	0.000	67.17
chrX	77142304	77142448	145	72	0.000	76.7
chrX	78783457	78783623	167	83	0.000	73.86
chrX	78808117	78808349	233	116	0.000	54.14
chrX	78930190	78930399	210	105	0.000	71.82
chrX	82780523	82780686	164	82	0.000	33.6
chrX	83474352	83474497	146	73	0.000	47.96
chrX	85416604	85416948	345	109	0.000	62.51
chrX	87732674	87732810	137	68	0.000	75.82
chrX	88148882	88149026	145	72	0.000	32.46
chrX	90042282	90042463	182	91	0.000	75.75
chrX	94319259	94319454	196	98	0.000	50.37
chrX	95162594	95162971	378	136	0.000	74.78
chrX	96946732	96946996	265	73	0.000	60.9
chrX	102731699	102731891	193	96	0.000	76.75
chrX	107527738	107528015	278	210	0.000	67.17
chrX	107879911	107880082	172	86	0.000	76.43
chrX	108945518	108945775	258	109	0.000	72.01
chrX	111180766	111180941	176	88	0.000	63.33

chrX	111528749	111528973	225	112	0.000	65.43
chrX	113363199	113363369	171	85	0.000	68.45
chrX	113990257	113990406	150	75	0.000	33.6
chrX	114615313	114615596	284	216	0.000	64.7
chrX	116017339	116017488	150	75	0.000	50.58
chrX	119437823	119437950	128	64	0.000	73.48
chrX	120545655	120545855	201	100	0.000	75
chrX	120734724	120735011	288	92	0.000	71.37
chrX	120969761	120969900	140	70	0.000	35.76
chrX	124008869	124008992	124	62	0.000	26.67
chrX	124705383	124705727	345	281	0.000	66.98
chrX	126285396	126285743	348	273	0.000	66.64
chrX	126557336	126557505	170	85	0.000	76.83
chrX	128766393	128766517	125	62	0.000	26.67
chrX	130848153	130848339	187	93	0.000	66.63
chrX	130894989	130895122	134	67	0.000	32.46
chrX	130996251	130996565	315	136	0.000	75.76
chrX	136133194	136133387	194	97	0.000	72.31
chrX	140303037	140303198	162	81	0.000	72.34
chrX	140341796	140341918	123	61	0.000	34.57
chrX	142569922	142570067	146	73	0.000	71.15
chrX	144601409	144601593	185	92	0.000	45.33
chrX	145061290	145061713	424	230	0.000	75.58
chrX	147140333	147140485	153	76	0.000	65.93
chrX	149012571	149012802	232	116	0.000	64.92
chrX	149852190	149852366	177	88	0.000	63.33
chrX	154460253	154460425	173	86	0.000	74.66
chrY	2856205	2856334	130	65	0.000	66.67
chrY	3148800	3148949	150	75	0.000	76.08
chrY	4283232	4283528	297	125	0.000	76.29
chrY	4745102	4745256	155	77	0.000	45.33
chrY	9573670	9573801	132	66	0.000	74.45
chrY	13420711	13421101	391	106	0.000	53.97
chrY	13467171	13469520	2350	1952	0.000	76.41
chrY	17478066	17478195	130	65	0.000	34.57
chrY	58823277	58823781	505	218	0.000	64.2
chrY	58825362	58825784	423	240	0.000	54.55
chrY	58827222	58827648	427	127	0.000	71.67
chrY	58828935	58829247	313	199	0.000	75.36
chrY	58834539	58835135	597	255	0.000	63.3
chrY	58838663	58839002	340	109	0.000	76.8
chrY	58844383	58844981	599	429	0.000	61.57
chrY	58845900	58846233	334	140	0.000	73.72
chrY	58846809	58847136	328	101	0.000	53.81
chrY	58847254	58847607	354	278	0.000	72.02
chrY	58860775	58860999	225	112	0.000	72.62
chrY	58871384	58871967	584	351	0.000	67.67
chrY	58886276	58886564	289	68	0.000	76.14
chrY	58887911	58888492	582	301	0.000	65.16
chrY	58888782	58889165	384	276	0.000	72.16
chrY	58891870	58892290	421	144	0.000	69.3
chrY	58892560	58893007	448	255	0.000	66.1
chrY	58904800	58905378	579	74	0.000	47.38
chrY	58912214	58912917	704	376	0.000	75.83
chrY	58915842	58916254	413	261	0.000	73.48
