# Supplementary material for: Thymoquinone upregulates IL17RD in controlling the growth and metastasis of triple negative breast cancer cells in vitro
Source: BMC Cancer. 2022 Jun 27;22:707. doi: 10.1186/s12885-022-09782-z (PMC9238053; doi:10.1186/s12885-022-09782-z)
Supplement: Supplementary file 5 — Additional file 5: Table S2. Methylated related genes. [file 12885_2022_9782_MOESM5_ESM.pdf]

Table S2: Methylated related genes

hgnc_id	hgnc_symbol	ensembl_gene_id	chromosome_name	strand	start_position	end_position	description
HGNC:14638	ABCA13	ENSG00000179869	7	1	48171458	48647496	ATP binding cassette subfamily A member 13 [Source:HGNC Symbol;Acc:HGNC:14638]
HGNC:38	ABCA8	ENSG00000141338	17	-1	68867292	68955392	ATP binding cassette subfamily A member 8 [Source:HGNC Symbol;Acc:HGNC:38]
HGNC:16486	ADAP1	ENSG00000105963	7	-1	897901	955407	ArfGAP with dual PH domains 1 [Source:HGNC Symbol;Acc:HGNC:16486]
HGNC:18989	ADGRF3	ENSG00000173567	2	-1	26308173	26346817	adhesion G protein-coupled receptor F3 [Source:HGNC Symbol;Acc:HGNC:18989]
HGNC:336	AGTR1	ENSG00000144891	3	1	148697784	148743008	angiotensin II receptor type 1 [Source:HGNC Symbol;Acc:HGNC:336]
HGNC:42024	ANKRD20A11P	ENSG00000215559	21	-1	13909574	13980437	ankyrin repeat domain 20 family member A11, pseudogene [Source:HGNC Symbol;Acc:HGNC:42024]
HGNC:20192	AP5M1	ENSG00000053770	14	1	57268909	57298742	adaptor related protein complex 5 mu 1 subunit [Source:HGNC Symbol;Acc:HGNC:20192]
HGNC:25540	ARHGEF10L	ENSG00000074964	1	1	17539835	17697874	Rho guanine nucleotide exchange factor 10 like [Source:HGNC Symbol;Acc:HGNC:25540]
HGNC:20730	ARMC9	ENSG00000135931	2	1	231198546	231374837	armadillo repeat containing 9 [Source:HGNC Symbol;Acc:HGNC:20730]
HGNC:16968	ARPP21	ENSG00000172995	3	1	35638945	35794496	cAMP regulated phosphoprotein 21 [Source:HGNC Symbol;Acc:HGNC:16968]
HGNC:28262	ATP1A1-AS1	ENSG00000203865	1	-1	116392247	116418622	ATP1A1 antisense RNA 1 [Source:HGNC Symbol;Acc:HGNC:28262]
HGNC:11405	BRSK2	ENSG00000174672	11	1	1389899	1462689	BR serine/threonine kinase 2 [Source:HGNC Symbol;Acc:HGNC:11405]
HGNC:1117	BSN	ENSG00000164061	3	1	49554489	49671545	bassoon presynaptic cytomatrix protein [Source:HGNC Symbol;Acc:HGNC:1117]

HGNC:42445	BSN-AS2	ENSG00000226913	3	-1	49549306	49554366	BSN antisense RNA 2 (head to head) [Source:HGNC Symbol;Acc:HGNC:42445]
HGNC:14326	C1QTNF3	ENSG00000082196	5	-1	34019448	34043832	C1q and TNF related 3 [Source:HGNC Symbol;Acc:HGNC:14326]
HGNC:14342	C1QTNF7	ENSG00000163145	4	1	15339818	15446166	C1q and TNF related 7 [Source:HGNC Symbol;Acc:HGNC:14342]
HGNC:1555	CBX5	ENSG00000094916	12	-1	54230940	54280133	chromobox 5 [Source:HGNC Symbol;Acc:HGNC:1555]
HGNC:10634	CCL7	ENSG00000108688	17	1	34270221	34272242	C-C motif chemokine ligand 7 [Source:HGNC Symbol;Acc:HGNC:10634]
HGNC:13733	CDH23	ENSG00000107736	10	1	71396934	71815947	cadherin related 23 [Source:HGNC Symbol;Acc:HGNC:13733]
HGNC:20359	CEP128	ENSG00000100629	14	-1	80476983	80959517	centrosomal protein 128 [Source:HGNC Symbol;Acc:HGNC:20359]
HGNC:25701	CHD9	ENSG00000177200	16	1	53055033	53329150	chromodomain helicase DNA binding protein 9 [Source:HGNC Symbol;Acc:HGNC:25701]
HGNC:43578	CHEK2P2	ENSG00000259156	15	1	20282744	20291586	checkpoint kinase 2 pseudogene 2 [Source:HGNC Symbol;Acc:HGNC:43578]
HGNC:25632	CPPED1	ENSG00000103381	16	-1	12659799	12804017	calcineurin like phosphoesterase domain containing 1 [Source:HGNC Symbol;Acc:HGNC:25632]
HGNC:29875	CRCT1	ENSG00000169509	1	1	152514502	152516010	cysteine rich C-terminal 1 [Source:HGNC Symbol;Acc:HGNC:29875]
HGNC:18966	CSAD	ENSG00000139631	12	-1	53157663	53180909	cysteine sulfinic acid decarboxylase [Source:HGNC Symbol;Acc:HGNC:18966]
HGNC:28589	CXorf38	ENSG00000185753	X	-1	40626921	40647554	chromosome X open reading frame 38 [Source:HGNC Symbol;Acc:HGNC:28589]
HGNC:2673	DAP3	ENSG00000132676	1	1	155687960	155739010	death associated protein 3 [Source:HGNC Symbol;Acc:HGNC:2673]
HGNC:24013	DNTTIP2	ENSG00000067334	1	-1	93866283	93879918	deoxynucleotidyltransferase terminal interacting protein 2 [Source:HGNC Symbol;Acc:HGNC:24013]
HGNC:15858	DZANK1	ENSG00000089091	20	-1	18383367	18467281	double zinc ribbon and ankyrin repeat domains 1 [Source:HGNC Symbol;Acc:HGNC:15858]

HGNC:26379	EFCAB3	ENSG00000172421		17	1	62343941	62416479	EF-hand calcium binding domain 3 [Source:HGNC Symbol;Acc:HGNC:26379]
HGNC:24599	ENOPH1	ENSG00000145293		4	1	82430562	82461091	enolase-phosphatase 1 [Source:HGNC Symbol;Acc:HGNC:24599]
HGNC:13595	FBXO24	ENSG00000106336		7	1	100583982	100601117	F-box protein 24 [Source:HGNC Symbol;Acc:HGNC:13595]
HGNC:3619	FCGR3A	ENSG00000203747		1	-1	161541759	161550737	Fc fragment of IgG receptor IIIa [Source:HGNC Symbol;Acc:HGNC:3619]
HGNC:19185	FRAS1	ENSG00000138759		4	1	78057570	78544269	Fraser extracellular matrix complex subunit 1 [Source:HGNC Symbol;Acc:HGNC:19185]
HGNC:4384	GNAI1	ENSG00000127955		7	1	80133955	80219402	G protein subunit alpha i1 [Source:HGNC Symbol;Acc:HGNC:4384]
HGNC:29925	GNL2	ENSG00000134697		1	-1	37566816	37595935	G protein nucleolar 2 [Source:HGNC Symbol;Acc:HGNC:29925]
HGNC:31042	GREB1L	ENSG00000141449		18	1	21242242	21525417	growth regulation by estrogen in breast cancer 1 like [Source:HGNC Symbol;Acc:HGNC:31042]
HGNC:25806	GSTCD	ENSG00000138780		4	1	105708778	105847728	glutathione S-transferase C-terminal domain containing [Source:HGNC Symbol;Acc:HGNC:25806]
HGNC:5031	HNRNPA1	ENSG00000135486		12	1	54280193	54287088	heterogeneous nuclear ribonucleoprotein A1 [Source:HGNC Symbol;Acc:HGNC:5031]
HGNC:5037	HNRNPDL	ENSG00000152795		4	-1	82422564	82430408	heterogeneous nuclear ribonucleoprotein D like [Source:HGNC Symbol;Acc:HGNC:5037]
HGNC:17616	IL17RD	ENSG00000144730		3	-1	57089982	57170306	interleukin 17 receptor D [Source:HGNC Symbol;Acc:HGNC:17616]
HGNC:25067	INTS12	ENSG00000138785		4	-1	105682627	105895986	integrator complex subunit 12 [Source:HGNC Symbol;Acc:HGNC:25067]
HGNC:6122	IRF7	ENSG00000185507		11	-1	612553	615999	interferon regulatory factor 7 [Source:HGNC Symbol;Acc:HGNC:6122]
HGNC:6122	IRF7	ENSG00000276561	CHR_HSCHR11_1_CTG8		-1	612805	616257	interferon regulatory factor 7 [Source:HGNC Symbol;Acc:HGNC:6122]
HGNC:13606	KDM2A	ENSG00000173120		11	1	67119269	67258087	lysine demethylase 2A [Source:HGNC Symbol;Acc:HGNC:13606]
HGNC:29219	KIAA1211	ENSG00000109265		4	1	56049073	56328625	KIAA1211 [Source:HGNC Symbol;Acc:HGNC:29219]

HGNC:29531	LARP1	ENSG00000155506		5	1	154712902	154817607	La ribonucleoprotein domain family member 1 [Source:HGNC Symbol;Acc:HGNC:29531]
HGNC:29461	LCE3A	ENSG00000185962		1	-1	152622834	152623103	late cornified envelope 3A [Source:HGNC Symbol;Acc:HGNC:29461]
HGNC:26865	LINC00303	ENSG00000176754		1	-1	204032447	204041265	long intergenic non-protein coding RNA 303 [Source:HGNC Symbol;Acc:HGNC:26865]
HGNC:27231	LINC00599	ENSG00000253230		8	-1	9900064	9905366	long intergenic non-protein coding RNA 599 [Source:HGNC Symbol;Acc:HGNC:27231]
HGNC:49796	LINC01239	ENSG00000234840		9	1	22646200	22824213	long intergenic non-protein coding RNA 1239 [Source:HGNC Symbol;Acc:HGNC:49796]
HGNC:52421	LINC01634	ENSG00000235295		22	1	18029385	18037968	long intergenic non-protein coding RNA 1634 [Source:HGNC Symbol;Acc:HGNC:52421]
HGNC:52582	LINC01792	ENSG00000237166		2	-1	200712305	200735177	long intergenic non-protein coding RNA 1792 [Source:HGNC Symbol;Acc:HGNC:52582]
HGNC:6691	LRCH4	ENSG00000077454		7	-1	100574011	100586153	leucine rich repeats and calponin homology domain containing 4 [Source:HGNC Symbol;Acc:HGNC:6691]
HGNC:24008	MIA3	ENSG00000154305		1	1	222618086	222668012	MIA family member 3, ER export factor [Source:HGNC Symbol;Acc:HGNC:24008]
HGNC:7095	MID1	ENSG00000101871	X		-1	10445310	10833654	midline 1 [Source:HGNC Symbol;Acc:HGNC:7095]
HGNC:31502	MIR124-1	ENSG00000284321		8	-1	9903388	9903472	microRNA 124-1 [Source:HGNC Symbol;Acc:HGNC:31502]
HGNC:35255	MIR1283-1	ENSG00000221421		19	1	53688481	53688567	microRNA 1283-1 [Source:HGNC Symbol;Acc:HGNC:35255]
HGNC:38372	MIR2909	ENSG00000276326		17	1	37033745	37033813	microRNA 2909 [Source:HGNC Symbol;Acc:HGNC:38372]
HGNC:41564	MIR3529	ENSG00000283484		15	-1	88611847	88611924	microRNA 3529 [Source:HGNC Symbol;Acc:HGNC:41564]
HGNC:32098	MIR519C	ENSG00000207788		19	1	53686469	53686555	microRNA 519c [Source:HGNC Symbol;Acc:HGNC:32098]
HGNC:32099	MIR520A	ENSG00000207594		19	1	53690881	53690965	microRNA 520a [Source:HGNC Symbol;Acc:HGNC:32099]
HGNC:32898	MIR642A	ENSG00000207773		19	1	45674928	45675024	microRNA 642a [Source:HGNC Symbol;Acc:HGNC:32898]

HGNC:38902	MIR642B	ENSG00000283212		19	-1	45674932	45675008	microRNA 642b [Source:HGNC Symbol;Acc:HGNC:38902]
HGNC:31639	MIR7-2	ENSG00000207703		15	1	88611825	88611934	microRNA 7-2 [Source:HGNC Symbol;Acc:HGNC:31639]
HGNC:29121	MTCL1	ENSG00000168502		18	1	8705661	8832778	microtubule crosslinking factor 1 [Source:HGNC Symbol;Acc:HGNC:29121]
HGNC:7454	MTMR7	ENSG00000003987		8	-1	17298030	17413528	myotubularin related protein 7 [Source:HGNC Symbol;Acc:HGNC:7454]
HGNC:1112	NCAPH	ENSG00000121152		2	1	96335787	96373845	non-SMC condensin I complex subunit H [Source:HGNC Symbol;Acc:HGNC:1112]
HGNC:29933	NLRC5	ENSG00000140853		16	1	56989485	57083531	NLR family CARD domain containing 5 [Source:HGNC Symbol;Acc:HGNC:29933]
HGNC:4525	NPFFR2	ENSG00000056291		4	1	72031804	72148067	neuropeptide FF receptor 2 [Source:HGNC Symbol;Acc:HGNC:4525]
HGNC:17881	NRSN1	ENSG00000152954		6	1	24126122	24154900	neurensin 1 [Source:HGNC Symbol;Acc:HGNC:17881]
HGNC:15346	OR11G2	ENSG00000196832		14	1	20190894	20201075	olfactory receptor family 11 subfamily G member 2 [Source:HGNC Symbol;Acc:HGNC:15346]
HGNC:15358	OR4N5	ENSG00000184394		14	1	20138820	20145471	olfactory receptor family 4 subfamily N member 5 [Source:HGNC Symbol;Acc:HGNC:15358]
HGNC:15682	PHC3	ENSG00000173889		3	-1	170086732	170181749	polyhomeotic homolog 3 [Source:HGNC Symbol;Acc:HGNC:15682]
HGNC:9248	PPFIA4	ENSG00000143847		1	1	203026498	203078740	PTPRF interacting protein alpha 4 [Source:HGNC Symbol;Acc:HGNC:9248]
HGNC:9534	PSMA5	ENSG00000143106		1	-1	109399031	109426427	proteasome subunit alpha 5 [Source:HGNC Symbol;Acc:HGNC:9534]
HGNC:9630	PTN	ENSG00000105894		7	-1	137227341	137343865	pleiotrophin [Source:HGNC Symbol;Acc:HGNC:9630]
HGNC:30297	RCC2	ENSG00000179051		1	-1	17406760	17439724	regulator of chromosome condensation 2 [Source:HGNC Symbol;Acc:HGNC:30297]
HGNC:30297	RCC2	ENSG00000281540	CHR_HG2095_PATCH		-1	17409469	17442433	regulator of chromosome condensation 2 [Source:HGNC Symbol;Acc:HGNC:30297]
HGNC:30457	RCCD1	ENSG00000166965		15	1	90954870	90963125	RCC1 domain containing 1 [Source:HGNC Symbol;Acc:HGNC:30457]
HGNC:9950	RECQL5	ENSG00000108469		17	-1	75626845	75667189	RecQ like helicase 5 [Source:HGNC Symbol;Acc:HGNC:9950]

HGNC:24152	RSRC1	ENSG00000174891		3	1	158105855	158545730	arginine and serine rich coiled-coil 1 [Source:HGNC Symbol;Acc:HGNC:24152]
HGNC:30285	RUFY3	ENSG00000018189		4	1	70704204	70807315	RUN and FYVE domain containing 3 [Source:HGNC Symbol;Acc:HGNC:30285]
HGNC:30179	SAC3D1	ENSG00000168061		11	1	65040901	65044828	SAC3 domain containing 1 [Source:HGNC Symbol;Acc:HGNC:30179]
HGNC:26009	SH3TC1	ENSG00000125089		4	1	8182072	8241803	SH3 domain and tetratricopeptide repeats 1 [Source:HGNC Symbol;Acc:HGNC:26009]
HGNC:10854	SHOX2	ENSG00000168779		3	-1	158095954	158106503	short stature homeobox 2 [Source:HGNC Symbol;Acc:HGNC:10854]
HGNC:23472	SLC25A28	ENSG00000155287		10	-1	99610522	99620609	solute carrier family 25 member 28 [Source:HGNC Symbol;Acc:HGNC:23472]
HGNC:20860	SLC39A12	ENSG00000148482		10	1	17951839	18043292	solute carrier family 39 member 12 [Source:HGNC Symbol;Acc:HGNC:20860]
HGNC:16472	SLC45A2	ENSG00000164175		5	-1	33944616	33984730	solute carrier family 45 member 2 [Source:HGNC Symbol;Acc:HGNC:16472]
HGNC:16472	SLC45A2	ENSG00000281919	CHR_HSCHR5_6_CTG1		-1	33946602	33956490	solute carrier family 45 member 2 [Source:HGNC Symbol;Acc:HGNC:16472]
HGNC:24105	STAMBPL1	ENSG00000138134		10	1	88879734	88975153	STAM binding protein like 1 [Source:HGNC Symbol;Acc:HGNC:24105]
HGNC:11470	SUPT6H	ENSG00000109111		17	1	28662091	28702684	SPT6 homolog, histone chaperone [Source:HGNC Symbol;Acc:HGNC:11470]
HGNC:30232	SZRD1	ENSG00000055070		1	1	16352575	16398145	SUZ RNA binding domain containing 1 [Source:HGNC Symbol;Acc:HGNC:30232]
HGNC:11566	TAPBP	ENSG00000231925		6	-1	33299694	33314387	TAP binding protein [Source:HGNC Symbol;Acc:HGNC:11566]
HGNC:11566	TAPBP	ENSG00000112493	CHR_HSCHR6_MHC_COX_CTG1		-1	33221298	33235989	TAP binding protein [Source:HGNC Symbol;Acc:HGNC:11566]
HGNC:11566	TAPBP	ENSG00000236490	CHR_HSCHR6_MHC_DBB_CTG1		-1	33277619	33292309	TAP binding protein [Source:HGNC Symbol;Acc:HGNC:11566]
HGNC:11566	TAPBP	ENSG00000206281	CHR_HSCHR6_MHC_QBL_CTG1		-1	33228522	33243221	TAP binding protein [Source:HGNC Symbol;Acc:HGNC:11566]

HGNC:11566	TAPBP	ENSG00000206208	CHR_HSCHR6_MHC_MCF_CTG1		-1	33470037	33484728	TAP binding protein [Source:HGNC Symbol;Acc:HGNC:11566]
HGNC:19112	TAS2R30	ENSG00000256188		12	-1	11132958	11134644	taste 2 receptor member 30 [Source:HGNC Symbol;Acc:HGNC:19112]
HGNC:19112	TAS2R30	ENSG00000262111	CHR_HSCHR12_2_CTG2		-1	11164906	11166592	taste 2 receptor member 30 [Source:HGNC Symbol;Acc:HGNC:19112]
HGNC:19112	TAS2R30	ENSG00000274699	CHR_HSCHR12_3_CTG2		-1	11129613	11130572	taste 2 receptor member 30 [Source:HGNC Symbol;Acc:HGNC:19112]
HGNC:30888	TBC1D31	ENSG00000156787		8	1	123041968	123152153	TBC1 domain family member 31 [Source:HGNC Symbol;Acc:HGNC:30888]
HGNC:11639	TCF7	ENSG00000081059		5	1	134114711	134151865	transcription factor 7 [Source:HGNC Symbol;Acc:HGNC:11639]
HGNC:11641	TCF7L2	ENSG00000148737		10	1	112950250	113167678	transcription factor 7 like 2 [Source:HGNC Symbol;Acc:HGNC:11641]
HGNC:17037	TFB1M	ENSG00000029639		6	-1	155257509	155314493	transcription factor B1, mitochondrial [Source:HGNC Symbol;Acc:HGNC:17037]
HGNC:26489	TMEM154	ENSG00000170006		4	-1	152618632	152680165	transmembrane protein 154 [Source:HGNC Symbol;Acc:HGNC:26489]
HGNC:30764	TRAIP	ENSG00000183763		3	-1	49828599	49856574	TRAF interacting protein [Source:HGNC Symbol;Acc:HGNC:30764]
HGNC:12962	TRIM26	ENSG00000228881	CHR_HSCHR6_MHC_APD_CTG1		-1	30176913	30205889	tripartite motif containing 26 [Source:HGNC Symbol;Acc:HGNC:12962]
HGNC:12962	TRIM26	ENSG00000234046	CHR_HSCHR6_MHC_SSTO_CTG1		-1	30174807	30203783	tripartite motif containing 26 [Source:HGNC Symbol;Acc:HGNC:12962]
HGNC:12962	TRIM26	ENSG00000226060	CHR_HSCHR6_MHC_DBB_CTG1		-1	30174734	30203710	tripartite motif containing 26 [Source:HGNC Symbol;Acc:HGNC:12962]
HGNC:12962	TRIM26	ENSG00000234127		6	-1	30184455	30213427	tripartite motif containing 26 [Source:HGNC Symbol;Acc:HGNC:12962]
HGNC:12962	TRIM26	ENSG00000137313	CHR_HSCHR6_MHC_QBL_CTG1		-1	30173958	30202936	tripartite motif containing 26 [Source:HGNC Symbol;Acc:HGNC:12962]

HGNC:12962	TRIM26	ENSG00000231641	CHR_HSCHR6_MHC_MCF_CTG1	-1	30262891	30291774	tripartite motif containing 26 [Source:HGNC Symbol;Acc:HGNC:12962]
HGNC:12962	TRIM26	ENSG00000231002	CHR_HSCHR6_MHC_MANN_CTG1	-1	30229518	30258487	tripartite motif containing 26 [Source:HGNC Symbol;Acc:HGNC:12962]
HGNC:12962	TRIM26	ENSG00000230230	CHR_HSCHR6_MHC_COX_CTG1	-1	30174354	30203317	tripartite motif containing 26 [Source:HGNC Symbol;Acc:HGNC:12962]
HGNC:16278	TRIM7	ENSG00000146054	5	-1	181193924	181205293	tripartite motif containing 7 [Source:HGNC Symbol;Acc:HGNC:16278]
HGNC:12373	TSHR	ENSG00000165409	14	1	80954989	81146302	thyroid stimulating hormone receptor [Source:HGNC Symbol;Acc:HGNC:12373]
HGNC:16256	TSPY26P	ENSG00000235217	20	-1	32186477	32190527	testis specific protein, Y-linked 26, pseudogene [Source:HGNC Symbol;Acc:HGNC:16256]
HGNC:14023	TTTY2	ENSG00000212855	Y	1	9740584	9758476	testis-specific transcript, Y-linked 2 (non-protein coding) [Source:HGNC Symbol;Acc:HGNC:14023]
HGNC:30866	UEVLD	ENSG00000151116	11	-1	18529609	18588747	UEV and lactate/malate dehyrogenase domains [Source:HGNC Symbol;Acc:HGNC:30866]
HGNC:41508	VWC2L-IT1	ENSG00000224257	2	1	214510196	214536890	VWC2L intronic transcript 1 [Source:HGNC Symbol;Acc:HGNC:41508]
HGNC:30238	WLS	ENSG00000116729	1	-1	68098473	68233120	wntless Wnt ligand secretion mediator [Source:HGNC Symbol;Acc:HGNC:30238]
HGNC:16265	WNT5B	ENSG00000111186	12	1	1529891	1647243	Wnt family member 5B [Source:HGNC Symbol;Acc:HGNC:16265]
HGNC:20876	WRNIP1	ENSG00000124535	6	1	2765414	2786952	Werner helicase interacting protein 1 [Source:HGNC Symbol;Acc:HGNC:20876]
HGNC:30935	YY1AP1	ENSG00000163374	1	-1	155659443	155689000	YY1 associated protein 1 [Source:HGNC Symbol;Acc:HGNC:30935]
HGNC:13085	ZBTB22	ENSG00000236104	6	-1	33314406	33317942	zinc finger and BTB domain containing 22 [Source:HGNC Symbol;Acc:HGNC:13085]
HGNC:13085	ZBTB22	ENSG00000229253	CHR_HSCHR6_MHC_COX_CTG1	-1	33236020	33239546	zinc finger and BTB domain containing 22 [Source:HGNC Symbol;Acc:HGNC:13085]

HGNC:13085	ZBTB22	ENSG00000227780	CHR_HSCHR6_MHC_DBB_CTG1	-1	33292340	33295864	zinc finger and BTB domain containing 22 [Source:HGNC Symbol;Acc:HGNC:13085]
HGNC:13085	ZBTB22	ENSG00000206280	CHR_HSCHR6_MHC_QBL_CTG1	-1	33243252	33246776	zinc finger and BTB domain containing 22 [Source:HGNC Symbol;Acc:HGNC:13085]
HGNC:13085	ZBTB22	ENSG00000237056	CHR_HSCHR6_MHC_MCF_CTG1	-1	33484759	33488283	zinc finger and BTB domain containing 22 [Source:HGNC Symbol;Acc:HGNC:13085]
HGNC:28328	ZFC3H1	ENSG00000133858	12	-1	71609472	71667725	zinc finger C3H1-type containing [Source:HGNC Symbol;Acc:HGNC:28328]
HGNC:28643	ZNF550	ENSG00000251369	19	-1	57535257	57559863	zinc finger protein 550 [Source:HGNC Symbol;Acc:HGNC:28643]
HGNC:27465	ZNF740	ENSG00000139651	12	1	53180700	53195141	zinc finger protein 740 [Source:HGNC Symbol;Acc:HGNC:27465]
HGNC:26745	ZNF781	ENSG00000196381	19	-1	37667751	37692322	zinc finger protein 781 [Source:HGNC Symbol;Acc:HGNC:26745]
HGNC:21037	ZSCAN18	ENSG00000121413	19	-1	58083838	58118427	zinc finger and SCAN domain containing 18 [Source:HGNC Symbol;Acc:HGNC:21037]
HGNC:23528	ZSWIM8	ENSG00000214655	10	1	73785582	73801797	zinc finger SWIM-type containing 8 [Source:HGNC Symbol;Acc:HGNC:23528]
