# Supplementary material for: Thymoquinone upregulates IL17RD in controlling the growth and metastasis of triple negative breast cancer cells in vitro
Source: BMC Cancer. 2022 Jun 27;22:707. doi: 10.1186/s12885-022-09782-z (PMC9238053; doi:10.1186/s12885-022-09782-z)
Supplement: Supplementary file 6 — Additional file 6: Table S3. GO analysis result. [file 12885_2022_9782_MOESM6_ESM.pdf]

Table S3: GO analysis result

Gene_set	P-value	#genes in universe	#Gene in Gene Set	#genes in comparison	#Genes in Overlap	Overlapped genes							
GO_REGULATION_OF_HEPATOCYTE_PROLIFERATION	0.032325758	45956	13	116	1	PTN							
GO_CHROMOSOME_ORGANIZATION	0.004160598	45956	1009	116	8	RECQL5	RCC2	SUPT6H	CHD9	TSPY26P	TCF7L2	NCAPH	KDM2A
GO_HINDBRAIN_DEVELOPMENT	0.04719889	45956	137	116	2	PTN	WLS						
GO_CHROMOSOME_SEGREGATION	0.03186936	45956	272	116	3	RECQL5	RCC2	NCAPH					
GO_CYTOKINE_METABOLIC_PROCESS	0.042062122	45956	17	116	1	IRF7							
GO_REGULATION_OF_INTERFERON_BETA_PRODUCTION	0.0055757	45956	44	116	2	IRF7	TRAIP						
GO_REGULATION_OF_LYASE_ACTIVITY	0.020112662	45956	86	116	2	TSHR	NPFFR2						
GO_EPITHELIUM_DEVELOPMENT	0.033004276	45956	945	116	6	PTN	TCF7	FRAS1	LCE3A	CRCT1	WNT5B		
GO_SECONDARY_METABOLITE_BIOSYNTHETIC_PROCESS	0.049300602	45956	20	116	1	SLC45A2							
GO_NEGATIVE_REGULATION_OF_CELL_DEVELOPMENT	0.041729527	45956	303	116	3	PTN	RUFY3	RCC2					
GO_NEGATIVE_REGULATION_OF_GENE_EXPRESSION	0.035723314	45956	1493	116	8	RECQL5	TCF7	IRF7	C1QTNF3	TCF7L2	SHOX2	NLRC5	CBX5
GO_NEGATIVE_REGULATION_OF_TELOMERE_MAINTENANCE_VIA_TELOMERASE	0.029876378	45956	12	116	1	HNRNPA1							
GO_CHEMICAL_HOMEOSTASIS	0.006861224	45956	874	116	7	SLC39A12	CCL7	C1QTNF3	CDH23	TCF7L2	SLC25A28	AGTR1	
GO_REGULATION_OF_MICROTUBULE_BASED_PROCESS	0.02390497	45956	243	116	3	SLC39A12	RCC2	MID1					

GO_CELL_DIVISION	0.001143533	45956	460	116	6	RECQL5	SAC3D1	RCC2	BRSK2	NCAPH	GNAI1				
GO_SECRETION_BY_CELL	0.035084743	45956	486	116	4	PPFIA4	WLS	BRSK2	MIA3						
GO_PROTEIN_LOCALIZATION_TO_KINETOCHORE	0.027420851	45956	11	116	1	RCC2									
GO_NEGATIVE_REGULATION_OF_TRANSCRIPTION_ELONGATION_FROM_RNA_POLYMERASE_II_PROMOTER	0.027420851	45956	11	116	1	RECQL5									
GO_POSITIVE_REGULATION_OF_RESPONSE_TO_STIMULUS	0.009708675	45956	1929	116	11	LARP1	IRF7	CCL7	WLS	FCGR3A	TCF7L2	SHOX2	PSMA5	NLRC5	AGTR1
GO_REGULATION_OF_CANONICAL_WNT_SIGNALING_PATHWAY	0.003097607	45956	236	116	4	WNT5B	WLS	TCF7L2	PSMA5						
GO_REGULATION_OF_ADAPTIVE_IMMUNE_RESPONSE	0.038882069	45956	123	116	2	SUPT6H	IRF7								
GO_POSITIVE_REGULATION_OF_CELL_COMMUNICATION	0.001756273	45956	1532	116	11	LARP1	IRF7	CCL7	PTN	C1QTNF3	WLS	TCF7L2	SHOX2	PSMA5	NLRC5
GO_REGULATION_OF_CELL_MORPHOGENESIS_INVOLVED_IN_DIFFERENTIATION	0.010697882	45956	337	116	4	RUFY3	SHOX2	RCC2	TCF7L2						
GO_NON_CANONICAL_WNT_SIGNALING_PATHWAY	0.049058723	45956	140	116	2	TCF7L2	PSMA5								
GO_ALTERNATIVE_MRNA_SPLICING_VIA_SPLICEOSOME	0.027420851	45956	11	116	1	RSRC1									
GO_POSITIVE_REGULATION_OF_NEURON_DIFFERENTIATION	0.042756334	45956	306	116	3	PTN	RUFY3	SHOX2							
GO_PEPTIDE_CROSS_LINKING	0.008899835	45956	56	116	2	CRCT1	LCE3A								
GO_POSITIVE_REGULATION_OF_LOCOMOTION	0.022122601	45956	420	116	4	WNT5B	RUFY3	CCL7	MIA3						
GO_TISSUE_MORPHOGENESIS	0.04651703	45956	533	116	4	WLS	PTN	SHOX2	FRAS1						
GO_RESPONSE_TO_ANGIOTENSIN	0.042062122	45956	17	116	1	AGTR1									
GO_INOSITOL_PHOSPHATE_CATABOLIC_PROCESS	0.027420851	45956	11	116	1	MTMR7									
GO_POSITIVE_REGULATION_OF_CELLULAR_PROTEIN_LOCALIZATION	0.013349458	45956	360	116	4	PTN	RCC2	RUFY3	TCF7L2						
GO_CYTOKINE_MEDIATED_SIGNALING_PATHWAY	0.0010455	45956	452	116	6	IRF7	CCL7	TRIM26	PSMA5	IL17RD	MID1				

GO_NEGATIVE_REGULATION_OF_LOCOMOTION	0.029266256	45956	263	116	3	PTN	TRIM26	MIA3						
GO_POLYOL_CATABOLIC_PROCESS	0.044480999	45956	18	116	1	MTMR7								
GO_CELL_CELL_SIGNALING	0.013409427	45956	767	116	6	PPFIA4	CCL7	WLS	NPFFR2	BSN	TSHR			
GO_REGULATION_OF_HORMONE_LEVELS	0.033322483	45956	478	116	4	C1QTNF3	AGTR1	BRSK2	TCF7L2					
GO_REGULATION_OF_DEFENSE_RESPONSE	0.012792659	45956	759	116	6	IRF7	CCL7	C1QTNF3	PSMA5	NLRC5	AGTR1			
GO_NEGATIVE_REGULATION_OF_ADHERENS_JUNCTION_ORGANIZATION	0.039637174	45956	16	116	1	RCC2								
GO_CHROMOSOME_SEPARATION	0.049300602	45956	20	116	1	RECQL5								
GO_NEGATIVE_REGULATION_OF_TYPE_I_INTERFERON_PRODUCTION	0.004627606	45956	40	116	2	NLRC5	TRAIP							
GO_POSITIVE_REGULATION_OF_BIOSYNTHETIC_PROCESS	0.039408956	45956	1805	116	9	LARP1	SUPT6H	TRIM26	HNRNPA1	TCF7L2	SHOX2	IRF7	NLRC5	TSHR
GO_NEGATIVE_REGULATION_OF_NITROGEN_COMPOUND_METABOLIC_PROCESS	0.038657866	45956	1517	116	8	RECQL5	TCF7	IRF7	HNRNPA1	TCF7L2	SHOX2	NLRC5	CBX5	
GO_NEGATIVE_REGULATION_OF_TUMOR_NECROSIS_FACTOR_MEDIATED_SIGNALING_PATHWAY	0.037206141	45956	15	116	1	TRAIP								
GO_REGULATION_OF_WNT_SIGNALING_PATHWAY	0.008052322	45956	310	116	4	WNT5B	WLS	TCF7L2	PSMA5					
GO_NEGATIVE_REGULATION_OF_CELL_JUNCTION_ASSEMBLY	0.049300602	45956	20	116	1	RCC2								
GO_DRUG_TRANSMEMBRANE_TRANSPORT	0.046893821	45956	19	116	1	ABCA8								
GO_REGULATION_OF_CORTICOSTEROID_HORMONE_SECRETION	0.037206141	45956	15	116	1	AGTR1								
GO_POSITIVE_REGULATION_OF_MACROPHAGE_DERIVED_FOAM_CELL_DIFFERENTIATION	0.037206141	45956	15	116	1	AGTR1								
GO_REGULATION_OF_NUCLEOBASE_CONTAINING_COMPOUND_TRANSPORT	0.029876378	45956	12	116	1	SUPT6H								
GO_WNT_SIGNALING_PATHWAY	0.002028103	45956	351	116	5	WNT5B	TCF7	WLS	TCF7L2	PSMA5				
GO_NEGATIVE_REGULATION_OF_SEQUENCE_SPECIFIC_DNA_BINDING_TRANSCRIPTION_FACTOR_ACTIVITY	0.046584872	45956	136	116	2	NLRC5	TCF7L2							

GO_PANCREAS_DEVELOPMENT	0.0147711	45956	73	116	2	WLS	TCF7L2							
GO_RNA_PROCESSING	0.019515139	45956	835	116	6	INTS12	SUPT6H	RSRC1	HNRNPA1	TFB1M	ZFC3H1			
GO_EMBRYONIC_DIGESTIVE_TRACT_MORPHOGENESIS	0.000838015	45956	17	116	2	TCF7	SHOX2							
GO_MELANIN_METABOLIC_PROCESS	0.034769008	45956	14	116	1	SLC45A2								
GO_POSITIVE_REGULATION_OF_RESPONSE_TO_EXTERNAL_STIMULUS	0.039383056	45956	296	116	3	LARP1	AGTR1	CCL7						
GO_POSITIVE_REGULATION_OF_CELL_CYCLE_G2_M_PHASE_TRANSITION	0.044480999	45956	18	116	1	RCC2								
GO_NEGATIVE_REGULATION_OF_STEM_CELL_PROLIFERATION	0.042062122	45956	17	116	1	PTN								
GO_REGULATION_OF_TRANSCRIPTION_ELONGATION_FROM_RNA_POLYMERASE_II_PROMOTER	0.001681153	45956	24	116	2	RECQL5	SUPT6H							
GO_DIGESTIVE_SYSTEM_DEVELOPMENT	0.006360735	45956	148	116	3	SHOX2	WLS	TCF7						
GO_DNA_CONFORMATION_CHANGE	0.032165857	45956	273	116	3	RECQL5	TSPY26P	NCAPH						
GO_REGULATION_OF_ADENYLATE_CYCLASE_ACTIVITY	0.013640563	45956	70	116	2	TSHR	NPFFR2							
GO_MITOTIC_CELL_CYCLE	0.013331212	45956	766	116	6	RECQL5	SAC3D1	RCC2	BRSK2	NCAPH	PSMA5			
GO_REGULATION_OF_MESENCHYMAL_CELL_PROLIFERATION	0.003361314	45956	34	116	2	PTN	SHOX2							
GO_REGULATION_OF_CELL_PROJECTION_ORGANIZATION	0.013833433	45956	558	116	5	SLC39A12	PTN	RCC2	SHOX2	RUFY3				
GO_POSITIVE_REGULATION_OF_MOLECULAR_FUNCTION	0.037808807	45956	1791	116	9	RCC2	CCL7	TRIM26	TCF7L2	ADAP1	PSMA5	TSHR	AGTR1	ARHGEF10L
GO_NITROGEN_COMPOUND_TRANSPORT	0.0399646	45956	507	116	4	TAPBP	PPFIA4	HNRNPA1	SUPT6H					
GO_CELLULAR_RESPONSE_TO_INTERFERON_GAMMA	0.000269059	45956	122	116	4	TRIM26	IRF7	CCL7	MID1					
GO_NEGATIVE_REGULATION_OF_HISTONE_METHYLATION	0.039637174	45956	16	116	1	SUPT6H								
GO_ORGANELLE_FISSION	0.008652038	45956	496	116	5	RECQL5	SAC3D1	RCC2	BRSK2	NCAPH				

GO_TRANSITION_METAL_ION_HOMEOSTASIS	0.029643693	4596	106	116	2	SLC39A12	SLC25A28								
GO_PROTEIN_LOCALIZATION_TO_CHROMOSOME_CENTROMERIC_REGION	0.032325758	4596	13	116	1	RCC2									
GO_POSITIVE_REGULATION_OF_LEUKOCYTE_MIGRATION	0.031201472	4596	109	116	2	CCL7	MIA3								
GO_REGULATION_OF_SECRETION	0.032513921	4596	699	116	5	C1QTNF3	AGTR1	WLS	BRSK2	TCF7L2					
GO_POSITIVE_REGULATION_OF_TRANSCRIPTION_ELONGATION_FROM_RNA_POLYMERASE_II_PROMOTER	0.032325758	4596	13	116	1	SUPT6H									
GO_REGULATION_OF_NUCLEOCYTOPLASMIC_TRANSPORT	0.018464497	4596	220	116	3	C1QTNF3	SUPT6H	TCF7L2							
GO_REGULATION_OF_PROTEIN_SECRETION	0.017244925	4596	389	116	4	C1QTNF3	WLS	BRSK2	TCF7L2						
GO_REGULATION_OF_PEPTIDE_TRANSPORT	0.027323229	4596	256	116	3	C1QTNF3	BRSK2	TCF7L2							
GO_CANONICAL_WNT_SIGNALING_PATHWAY	0.024212789	4596	95	116	2	TCF7	TCF7L2								
GO_KERATINOCYTE_DIFFERENTIATION	0.027119515	4596	101	116	2	CRCT1	LCE3A								
GO_NEGATIVE_REGULATION_OF_GLIAL_CELL_PROLIFERATION	0.027420851	4596	11	116	1	PTN									
GO_POSITIVE_REGULATION_OF_PROTEIN_SECRETION	0.016546482	4596	211	116	3	C1QTNF3	WLS	TCF7L2							
GO_REGULATION_OF_STEROID_HORMONE_SECRETION	0.049300602	4596	20	116	1	AGTR1									
GO_EMBRYONIC_DIGESTIVE_TRACT_DEVELOPMENT	0.003168799	4596	33	116	2	TCF7	SHOX2								
GO_CELLULAR_RESPONSE_TO_ORGANIC_SUBSTANCE	0.000240652	4596	1848	116	14	GNAI1	PTN	TCF7	IRF7	CCL7	TRIM26	HNRNPA1	NPFFR2	WNT5B	PSMA5
GO_REGULATION_OF_ENDOCRINE_PROCESS	0.006604898	4596	48	116	2	C1QTNF3	AGTR1								
GO_POSITIVE_REGULATION_OF_IMMUNE_SYSTEM_PROCESS	0.022957051	4596	867	116	6	IRF7	CCL7	FCGR3A	MIA3	PSMA5	NLRC5				
GO_REGULATION_OF_HORMONE_SECRETION	0.004487659	4596	262	116	4	C1QTNF3	AGTR1	BRSK2	TCF7L2						
GO_NEGATIVE_REGULATION_OF_TELOMERE_MAINTENANCE_VIA_TELOMERE_LENGTHENING	0.042062122	4596	17	116	1	HNRNPA1									

GO_CHONDROCYTE_DIFFERENTIATION	0.010162251	45956	60	116	2	WNT5B	SHOX2								
GO_REGULATION_OF_CELLULAR_LOCALIZATION	0.005169746	45956	1277	116	9	PTN	RCC2	SUPT6H	BRSK2	C1QTNF3	RUFY3	WLS	TCF7L2	AGTR1	
GO_ANTIGEN_PROCESSING_AND_PRESENTATION_OF_PEPTIDE_ANTIGEN_VIA_MHC_CLASS_I	0.022351258	45956	91	116	2	TAPBP	PSMA5								
GO_RESPONSE_TO_PLATELET_DERIVED_GROWTH_FACTOR	0.044480999	45956	18	116	1	PTN									
GO_INNATE_IMMUNE_RESPONSE	0.020658209	45956	619	116	5	NLRC5	TRIM26	IRF7	CCL7	MID1					
GO_G_PROTEIN_COUPLED_RECEPTOR_SIGNALING_PATHWAY	0.010923836	45956	1193	116	8	CCL7	NPFFR2	OR4N5	TAS2R30	TSHR	AGTR1	OR11G2	GNAI1		
GO_DIGESTIVE_TRACT_MORPHOGENESIS	0.006604898	45956	48	116	2	TCF7	SHOX2								
GO_NEGATIVE_REGULATION_OF_CELL_MORPHOGENESIS_INVOLVED_IN_DIFFERENTIATION	0.035509302	45956	117	116	2	RUFY3	RCC2								
GO_REGULATION_OF_LYMPHOCYTE_CHEMOTAXIS	0.049300602	45956	20	116	1	CCL7									
GO_MITOTIC_NUCLEAR_DIVISION	0.002289921	45956	361	116	5	RECQL5	SAC3D1	RCC2	BRSK2	NCAPH					
GO_REGULATION_OF_RESPONSE_TO_STRESS	0.012241624	45956	1468	116	9	LARP1	IRF7	CCL7	C1QTNF3	BRSK2	PSMA5	NLRC5	AGTR1	MID1	
GO_REGULATION_OF_CELL_DIFFERENTIATION	0.004603313	45956	1492	116	10	SLC39A12	PTN	RCC2	IRF7	RUFY3	TCF7L2	SHOX2	SUPT6H	WNT5B	AGTR1
GO_METHIONINE_BIOSYNTHETIC_PROCESS	0.034769008	45956	14	116	1	ENOPH1									
GO_POSITIVE_REGULATION_OF_HORMONE_SECRETION	0.036620314	45956	119	116	2	C1QTNF3	TCF7L2								
GO_REGULATION_OF_INTRACELLULAR_PROTEIN_TRANSPORT	0.016107126	45956	381	116	4	C1QTNF3	RUFY3	BRSK2	TCF7L2						
GO_CELLULAR_RESPONSE_TO_ETHANOL	0.032325758	45956	13	116	1	CCL7									
GO_CAMERA_TYPE_EYE_PHOTORECEPTOR_CELL_DIFFERENTIATION	0.037206141	45956	15	116	1	PTN									
GO_NEGATIVE_REGULATION_OF_CHROMOSOME_ORGANIZATION	0.024687799	45956	96	116	2	HNRNPA1	SUPT6H								
GO_METHIONINE_METABOLIC_PROCESS	0.044480999	45956	18	116	1	ENOPH1									

GO_REGULATION_OF_ATTACHMENT_OF_SPINDLE_MICROTUBULES_TO_KINETOCHORE	0.027420851	45956	11	116	1	RCC2							
GO_EMBRYONIC_MORPHOGENESIS	0.048109336	45956	539	116	4	WLS	TCF7	SHOX2	FRAS1				
GO_NEGATIVE_REGULATION_OF_CANONICAL_WNT_SIGNALING_PATHWAY	0.008147049	45956	162	116	3	WNT5B	TCF7L2	PSMA5					
GO_INTERFERON_GAMMA_MEDIATED_SIGNALING_PATHWAY	0.000758304	45956	70	116	3	TRIM26	IRF7	MID1					
GO_EMBRYONIC_SKELETAL_JOINT_DEVELOPMENT	0.034769008	45956	14	116	1	SHOX2							
GO_POSITIVE_REGULATION_OF_GLYCOPROTEIN_METABOLIC_PROCESS	0.049300602	45956	20	116	1	TCF7L2							
GO_POSITIVE_REGULATION_OF_INNATE_IMMUNE_RESPONSE	0.024671814	45956	246	116	3	NLRC5	IRF7	PSMA5					
GO_REGULATION_OF_PROTEIN_LOCALIZATION	0.010555115	45956	950	116	7	PTN	RCC2	BRSK2	C1QTNF3	RUFY3	WLS	TCF7L2	
GO_DIVALENT_INORGANIC_CATION_HOMEOSTASIS	0.011353382	45956	343	116	4	SLC39A12	AGTR1	CDH23	CCL7				
GO_NEGATIVE_REGULATION_OF_RESPONSE_TO_CYTOKINE_STIMULUS	0.0055757	45956	44	116	2	NLRC5	TRAIP						
GO_CELLULAR_RESPONSE_TO_CYTOKINE_STIMULUS	0.000905382	45956	606	116	7	TCF7	IRF7	CCL7	TRIM26	PSMA5	IL17RD	MID1	
GO_FAT_CELL_DIFFERENTIATION	0.002502184	45956	106	116	3	WNT5B	TCF7L2	C1QTNF3					
GO_REGULATION_OF_CYTOPLASMIC_TRANSPORT	0.03397709	45956	481	116	4	C1QTNF3	SUPT6H	BRSK2	TCF7L2				
GO_REGULATION_OF_PHOSPHOLIPASE_A2_ACTIVITY	0.027420851	45956	11	116	1	AGTR1							
GO_IMMUNE_RESPONSE	0.006896984	45956	1100	116	8	TCF7	IRF7	CCL7	TRIM26	FCGR3A	TAPBP	NLRC5	MID1
GO_POSITIVE_REGULATION_OF_RESPONSE_TO_CYTOKINE_STIMULUS	0.003168799	45956	33	116	2	NLRC5	IRF7						
GO_NEGATIVE_REGULATION_OF_VIRAL_ENTRY_INTO_HOST_CELL	0.046893821	45956	19	116	1	TRIM26							
GO_CARTILAGE_DEVELOPMENT_INVOLVED_IN_ENDOCHONDRAL_BONE_MORPHOGENESIS	0.046893821	45956	19	116	1	SHOX2							
GO_CELL_CYCLE	0.018646341	45956	1316	116	8	RECQL5	SAC3D1	RCC2	BRSK2	PSMA5	TCF7L2	NCAPH	GNAI1

GO_POSITIVE_REGULATION_OF_CANONICAL_WNT_SIGNALING_PATHWAY	0.036620314	45956	119	116	2	WLS	PSMA5					
GO_REGULATION_OF_NEURON_PROJECTION_DEVELOPMENT	0.020145012	45956	408	116	4	SLC39A12	PTN	RUFY3	SHOX2			
GO_REGULATION_OF_STEM_CELL_PROLIFERATION	0.020996168	45956	88	116	2	PTN	SHOX2					
GO_REGULATION_OF_MONOCYTE_DIFFERENTIATION	0.034769008	45956	14	116	1	IRF7						
GO_DEPHOSPHORYLATION	0.036151829	45956	286	116	3	MTMR7	ENOPH1	CPPED1				
GO_REGULATION_OF_TYPE_I_INTERFERON_MEDIATED_SIGNALING_PATHWAY	0.004403457	45956	39	116	2	NLRC5	IRF7					
GO_REGULATION_OF_ANATOMICAL_STRUCTURE_MORPHOGENESIS	0.015154992	45956	1021	116	7	PTN	RCC2	CCL7	RUFY3	TCF7L2	SHOX2	PSMA5
GO_TOR_SIGNALING	0.039637174	45956	16	116	1	LARP1						
GO_THALAMUS_DEVELOPMENT	0.034769008	45956	14	116	1	PTN						
GO_NEGATIVE_REGULATION_OF_CHEMOKINE_PRODUCTION	0.039637174	45956	16	116	1	C1QTNF3						
GO_CELLULAR_RESPONSE_TO_ALCOHOL	0.034411749	45956	115	116	2	PTN	CCL7					
GO_NEGATIVE_REGULATION_OF_NF_KAPPAB_IMPORT_INTO_NUCLEUS	0.044480999	45956	18	116	1	C1QTNF3						
GO_REGULATION_OF_RETROGRADE_PROTEIN_TRANSPORT_ER_TO_CYTOSOL	0.032325758	45956	13	116	1	BRSK2						
GO_ION_HOMEOSTASIS	0.015659739	45956	576	116	5	SLC39A12	AGTR1	SLC25A28	CDH23	CCL7		
GO_NEGATIVE_REGULATION_OF_MYELOID_CELL_APOPTOTIC_PROCESS	0.034769008	45956	14	116	1	IRF7						
GO_POSITIVE_REGULATION_OF_INTERFERON_ALPHA_PRODUCTION	0.039637174	45956	16	116	1	IRF7						
GO_VIRAL_LATENCY	0.027420851	45956	11	116	1	IRF7						
GO_REGULATION_OF_INFLAMMATORY_RESPONSE	0.038725399	45956	294	116	3	C1QTNF3	AGTR1	CCL7				
GO_RESPONSE_TO_INTERFERON_GAMMA	0.000504301	45956	144	116	4	TRIM26	IRF7	CCL7	MID1			

GO_LOW_DENSITY_LIPOPROTEIN_PARTICLE_REMODELING	0.027420851	45956	11	116	1	AGTR1							
GO_CELLULAR_CHEMICAL_HOMEOSTASIS	0.015034025	45956	570	116	5	SLC39A12	C1QTNF3	AGTR1	CDH23	CCL7			
GO_POSITIVE_REGULATION_OF_CELL_DEVELOPMENT	0.032035695	45956	472	116	4	PTN	RUFY3	SHOX2	TCF7L2				
GO_REGULATION_OF_CELLULAR_COMPONENT_MOVEMENT	0.013725508	45956	771	116	6	PTN	RCC2	CCL7	RUFY3	MIA3	WNT5B		
GO_REGULATION_OF_PROTEIN_EXIT_FROM_ENDOPLASMIC_RETICULUM	0.046893821	45956	19	116	1	BRSK2							
GO_SULFUR_AMINO_ACID_BIOSYNTHETIC_PROCESS	0.046893821	45956	19	116	1	ENOPH1							
GO_CELLULAR_RESPONSE_TO_VITAMIN_D	0.034769008	45956	14	116	1	PTN							
GO_POSITIVE_REGULATION_OF_PROTEIN_EXPORT_FROM_NUCLEUS	0.046893821	45956	19	116	1	TCF7L2							
GO_REGULATION_OF_RESPONSE_TO_CYTOKINE_STIMULUS	0.005898189	45956	144	116	3	NLRC5	IRF7	TRAIP					
GO_BETA_CATENIN_TCF_COMPLEX_ASSEMBLY	0.005331007	45956	43	116	2	TCF7	TCF7L2						
GO_RESPONSE_TO_CYTOKINE	0.002298524	45956	714	116	7	TCF7	IRF7	CCL7	TRIM26	PSMA5	IL17RD	MID1	
GO_POSITIVE_REGULATION_OF_CELL_DIFFERENTIATION	0.018321058	45956	823	116	6	PTN	RUFY3	TCF7L2	SHOX2	WNT5B	AGTR1		
GO_NEGATIVE_REGULATION_OF_GLUCONEOGENESIS	0.032325758	45956	13	116	1	C1QTNF3							
GO_NEGATIVE_REGULATION_OF_DNA_TEMPLATED_TRANSCRIPTION_ELONGATION	0.032325758	45956	13	116	1	RECQL5							
GO_POSITIVE_REGULATION_OF_INFLAMMATORY_RESPONSE	0.033327817	45956	113	116	2	AGTR1	CCL7						
GO_REGULATION_OF_SULFUR_METABOLIC_PROCESS	0.049300602	45956	20	116	1	TCF7L2							
GO_SKIN_DEVELOPMENT	0.016546482	45956	211	116	3	CRCT1	FRAS1	LCE3A					
GO_CELLULAR_RESPONSE_TO_STRESS	0.045005248	45956	1565	116	8	RECQL5	PTN	IRF7	BRSK2	DZANK1	WRNIP1	KDM2A	ARPP21
GO_EPITHELIAL_CELL_DIFFERENTIATION	0.037131023	45956	495	116	4	WNT5B	CRCT1	LCE3A	PTN				

GO_REGULATION_OF_CELL_SHAPE	0.048435834	45956	139	1116	2	PTN	CCL7			
GO_REGULATION_OF_BLOOD_VOLUME_BY_RENIN_ANGIOTENSIN	0.027420851	45956	11	1116	1	AGTR1				
GO_NEGATIVE_REGULATION_OF_CYTOKINE_PRODUCTION	0.016546482	45956	211	1116	3	C1QTNF3	TRAIP	NLRC5		
GO_NEGATIVE_REGULATION_OF_WNT_SIGNALING_PATHWAY	0.013797053	45956	197	1116	3	WNT5B	TCF7L2	PSMA5		
GO_REGULATION_OF_CELLULAR_PROTEIN_LOCALIZATION	0.002849984	45956	552	1116	6	PTN	RCC2	BRSK2	C1QTNF3	RUFY3	TCF7L2
GO_NEGATIVE_REGULATION_OF_VIRAL_RELEASE_FROM_HOST_CELL	0.039637174	45956	16	1116	1	TRIM26				
GO_REGULATION_OF_INTRACELLULAR_TRANSPORT	0.020913273	45956	621	1116	5	C1QTNF3	RUFY3	SUPT6H	BRSK2	TCF7L2
GO_CELLULAR_HOMEOSTASIS	0.028750925	45956	676	1116	5	SLC39A12	C1QTNF3	AGTR1	CDH23	CCL7
GO_REGULATION_OF_INTERFERON_ALPHA_PRODUCTION	0.046893821	45956	19	1116	1	IRF7				
GO_NEGATIVE_REGULATION_OF_TRANSCRIPTION_FROM_RNA_POLYMERASE_II_PROMOTER	0.039956907	45956	740	1116	5	RECQL5	TCF7	SHOX2	IRF7	TCF7L2
GO_REGULATION_OF_CELL_DEVELOPMENT	0.019616988	45956	836	1116	6	SLC39A12	PTN	RCC2	RUFY3	TCF7L2	SHOX2
GO_NEGATIVE_REGULATION_OF_CELLULAR_COMPONENT_ORGANIZATION	0.007948486	45956	684	1116	6	PTN	RCC2	SUPT6H	RUFY3	HNRNPA1	MID1
GO_POSITIVE_REGULATION_OF_DEFENSE_RESPONSE	0.002373053	45956	364	1116	5	NLRC5	AGTR1	IRF7	CCL7	PSMA5
GO_ANTIGEN_PROCESSING_AND_PRESENTATION_OF_ENDOGENOUS_ANTIGEN	0.042062122	45956	17	1116	1	TAPBP				
GO_REGULATION_OF_CAMP_DEPENDENT_PROTEIN_KINASE_ACTIVITY	0.034769008	45956	14	1116	1	NPFFR2				
GO_REGULATION_OF_CAMP_METABOLIC_PROCESS	0.042371656	45956	129	1116	2	TSHR	NPFFR2			
GO_PHOSPHOLIPID_EFFLUX	0.034769008	45956	14	1116	1	ABCA13				
GO_RRNA_METHYLATION	0.046893821	45956	19	1116	1	TFB1M				
GO_REGULATION_OF_TYPE_I_INTERFERON_PRODUCTION	0.002850823	45956	111	1116	3	NLRC5	IRF7	TRAIP		

GO_CELL_CYCLE_PROCESS	0.02003493	45956	1081	116	7	RECQL5	SAC3D1	RCC2	BRSK2	PSMA5	TCF7L2	NCAPH	
GO_TISSUE_DEVELOPMENT	0.038783558	45956	1518	116	8	PTN	TCF7	FRAS1	WLS	LCE3A	CRCT1	SHOX2	WNT5B
GO_POSITIVE_REGULATION_OF_ESTABLISHMENT_OF_PROTEIN_LOCALIZATION	0.041673113	45956	514	116	4	C1QTNF3	RUFY3	WLS	TCF7L2				
GO_POSITIVE_REGULATION_OF_AXONOGENESIS	0.013272499	45956	69	116	2	RUFY3	SHOX2						
GO_NEGATIVE_REGULATION_OF_CELL_COMMUNICATION	0.03178496	45956	1192	116	7	C1QTNF3	WNT5B	TRAIP	TCF7L2	PSMA5	NLRC5	GNAI1	
GO_POSITIVE_REGULATION_OF_NEURON_PROJECTION_DEVELOPMENT	0.021206197	45956	232	116	3	PTN	RUFY3	SHOX2					
GO_LENS_FIBER_CELL_DEVELOPMENT	0.029876378	45956	12	116	1	WNT5B							
GO_V_D_J_RECOMBINATION	0.039637174	45956	16	116	1	TCF7							
GO_ANTIGEN_PROCESSING_AND_PRESENTATION_OF_ENDOGENOUS_PEPTIDE_ANTIGEN	0.034769008	45956	14	116	1	TAPBP							
GO_REGULATION_OF_DNA_TEMPLATED_TRANSCRIPTION_ELONGATION	0.0055757	45956	44	116	2	RECQL5	SUPT6H						
GO_DNA_METABOLIC_PROCESS	0.04352712	45956	758	116	5	KDM2A	RECQL5	TCF7	TCF7L2	WRNIP1			
GO_MITOTIC_CHROMOSOME_CONDENSATION	0.034769008	45956	14	116	1	NCAPH							
GO_NEGATIVE_REGULATION_OF_SUBSTRATE_ADHESION_DEPENDENT_CELL_SPREADING	0.032325758	45956	13	116	1	RCC2							
GO_POSITIVE_REGULATION_OF_CYTOSOLIC_CALCIUM_ION_CONCENTRATION_INVOLVED_IN_PHOSPHOLIPASE_C_ACTIVATING_G_PROTEIN_COUPLED_SIGNALING_PATHWAY	0.034769008	45956	14	116	1	AGTR1							
GO_ESTROUS_CYCLE	0.046893821	45956	19	116	1	PTN							
GO_REGULATION_OF_CELL_MORPHOGENESIS	0.002849984	45956	552	116	6	PTN	RCC2	CCL7	RUFY3	TCF7L2	SHOX2		
GO_POSITIVE_REGULATION_OF_CELL_MORPHOGENESIS_INVOLVED_IN_DIFFERENTIATION	0.008147049	45956	162	116	3	RUFY3	SHOX2	TCF7L2					
GO_POSITIVE_REGULATION_OF_CELL_PROJECTION_ORGANIZATION	0.041729527	45956	303	116	3	PTN	RUFY3	SHOX2					
